# NIR‐II Fluorescent Protein Created by In Situ Albumin‐Tagging for Sensitive and Specific Imaging of Blood‐Brain Barrier Disruption

**DOI:** 10.1002/advs.202500443

**Published:** 2025-02-25

**Authors:** Jiajun Xu, Yijing Du, Ningning Zhu, Jia Li, Yuewei Zhang, Ding Zhou, Shoujun Zhu

**Affiliations:** ^1^ Joint Laboratory of Opto‐Functional Theranostics in Medicine and Chemistry The First Hospital of Jilin University Changchun 130021 P. R. China; ^2^ State Key Laboratory of Supramolecular Structure and Materials Center for Supramolecular Chemical Biology College of Chemistry Jilin University Changchun 130012 P. R. China; ^3^ Key Laboratory of Medicinal Chemistry and Molecular Diagnosis of the Ministry of Education Key Laboratory of Chemical Biology of Hebei Province College of Chemistry and Materials Science Hebei University Baoding 071002 P. R. China; ^4^ Jilin Provincial Key Laboratory of Tooth Development and Bone Remodeling School and Hospital of Stomatology Jilin University Changchun 130021 P. R. China

**Keywords:** blood‐brain barrier disruption imaging, covalent binding, cyanine dye, in situ albumin‐tagging, NIR‐II fluorescent protein

## Abstract

Imaging albumin in vivo is a reliable strategy to visualize blood‐brain barrier (BBB) disruption by detecting the dye‐labeled albumin leaking into brain parenchyma. Although Evans Blue (EB) and indocyanine green (ICG) dyes have been applied to assess BBB impairment, their naked‐eye observation or near‐infrared‐I (NIR‐I) imaging window limit the imaging sensitivity and contrast for this promising “albumin‐based” strategy. Herein, an albumin‐specific tagged near‐infrared‐II (NIR‐II) probe is engineered as a chromophore to construct fluorescent proteins (FPs) in situ for assessing BBB disruption in stroke. The optimized chromophore, C7‐1080, can covalently bind to albumin through nucleophilic substitution, forming FPs without adjuvant. Notably, the albumin effectively acts as a brightness enhancer and stability regulator for chromophores through the tight clamping effect. Theoretical simulation, proteomics, and protein mutation techniques are employed to investigate the binding behavior between albumin and chromophore. The in situ NIR‐II FPs construction strategy facilitates high‐precision dual‐channel imaging of BBB disruption and cerebral vessels during ischemic stroke when combined with the IR‐808Ac probe. Overall, the in situ albumin‐specific tag holds promise for diagnosing and monitoring strokes, presenting a tool for investigating the progression and therapeutic responses of related diseases.

## Graphical abstract

The breakdown of the blood‐brain barrier (BBB) after cerebral ischemia can cause brain injury and induce potentially fatal consequences. Currently, there exist few dependable and low‐cost imaging techniques for visualizing the BBB. This investigation effectively engineered an albumin‐specific covalently tagged near‐infrared‐II (NIR‐II) dye, serving as a chromophore, to construct fluorescent proteins in situ for evaluating BBB disruption during stroke.

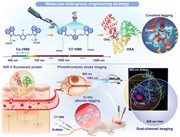

## 1 Introduction

The blood‐brain barrier (BBB) is a critical component of the central nervous system, its integrity is essential for maintaining brain homeostasis and preventing the entry of neurotoxic agents.^[^ ^1^, ^2^, ^3^ ^]^ However, under various pathological conditions, such as stroke and traumatic brain injury, the BBB can become disrupted, leading to the leakage of blood components into the brain parenchyma.^[^ ^4^, ^5^, ^6^ ^]^ Imaging BBB disruption is crucial for understanding the pathogenesis of these diseases and evaluating the effectiveness of therapeutic interventions.^[^ ^7^, ^8^ ^]^ In vivo imaging of BBB disruption has emerged as a robust tool to visualize and quantify the leakage of blood components in real time.^[^ ^9^, ^10^ ^]^ Among various imaging modalities, near‐infrared imaging (especially in the near‐infrared‐II (NIR‐II) window) has gained great attention due to its low tissue autofluorescence, deep tissue penetration, high imaging contrast, etc.^[^ ^11^, ^12^, ^13^, ^14^, ^15^, ^16^, ^17^, ^18^ ^]^

To achieve optimal targeting for in vivo imaging, NIR‐II fluorescent probes, whether organic dyes or inorganic nanomaterials require conjugation with targeting antibodies or peptides.^[^ ^19^, ^20^, ^21^, ^22^, ^23^ ^]^ By conjugating specific molecules or cells with these probes, researchers can non‐invasively observe biological processes in vivo with remarkable spatial and temporal precision. Fluorescent proteins, on the other hand, offer an alternative approach.^[^ ^24^, ^25^ ^]^ Infrared fluorescent proteins (iRFPs), in particular, have demonstrated superior in vivo imaging ability.^[^ ^26^, ^27^, ^28^ ^]^ These fluorophores are typically genetically encoded, enabling their expression within the target tissue or organism. However, a limitation of iRFPs is their slow and oxygen‐dependent maturation process.^[^ ^29^, ^30^ ^]^

Imaging albumin in vivo has the potential to directly visualize BBB disruption by detecting the extravasation of dye‐labeled albumin into the brain parenchyma.^[^ ^31^, ^32^ ^]^ Currently, Evans Blue (EB) dye and clinically‐available indocyanine green (ICG) have been successfully utilized to assess blood‐brain barrier impairment due to their potential serum albumin‐binding ability.^[^ ^32^, ^33^, ^34^, ^35^ ^]^ Despite their contributions to blood‐brain barrier research, challenges remain. The limited binding specificity between dye molecules and albumin often results in indiscriminate accumulation in vivo. This can lead to severe adverse effects such as pulmonary embolism or toxic metabolic encephalopathy, especially at higher concentrations.^[^ ^36^ ^]^ Moreover, current optical methods for BBB disruption detection typically involve in vitro naked‐eye observation or near‐infrared‐I (NIR‐I) imaging, making it challenging to achieve high‐precision lesion detection in vivo. Therefore, an ideal approach is to develop a NIR‐II dye that can bind albumin in situ to form a NIR‐II fluorescent protein with greatly enhanced brightness. Such endogenously formed NIR‐II fluorescent protein will combine the targeting specificity of albumin with the imaging advantages of NIR‐II fluorescence, resulting in an ideal imaging agent for BBB disruption studies.

**Scheme 1:**
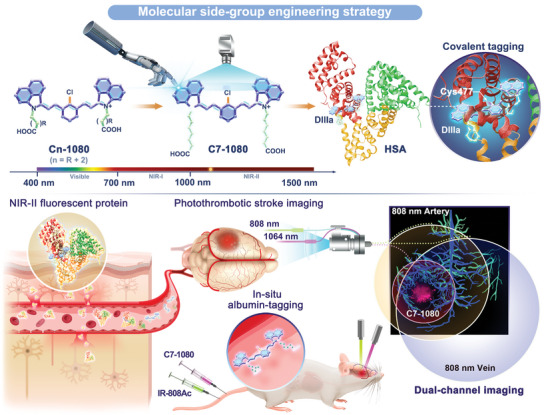
In situ formed NIR‐II fluorescent protein for sensitive and specific imaging of blood‐brain barrier disruption during photothrombotic stroke.

Our previous work has focused on the development of structural‐specific dyes to create biomimetic NIR‐II fluorescence proteins (FPs).^[^ ^37^, ^38^, ^39^ ^]^ However, the ability of existing NIR‐II dyes to bind specifically to albumin in situ (physiological conditions) has been limited. In this study, we rationally applied the molecular side‐group engineering strategies to further optimize the dye structure, endowing it with exceptional and selective albumin‐tagging properties. In vitro results confirmed that the role of NIR‐II albumin‐seeking dye (C7‐1080) as a chromophore, could undergo nucleophilic substitution reaction with cysteine in the hydrophobic cavity of human serum albumin (HSA) at room or physiological temperature without any catalyst to complete site‐specific covalently binding, thus forming NIR‐II FPs. Notably, the HSA as the outer shell improved the brightness and photostability of the chromophores through the tight clamping effect. More importantly, in situ NIR‐II FPs generated by the interaction of dyes with dependable albumin provided precise localization of BBB disruption in stroke (**Scheme** **1**). This work provides a proof‐of‐concept for the construction of in situ NIR‐II FPs from a biomimetic perspective, potentially offering a robust technical tool for the investigation of stroke‐related diseases.

## 2 Results and Discussion

### 2.1 Synthesis and Characterization of the Cn‐1080 Dyes as Albumin Tag

**Figure 1:**
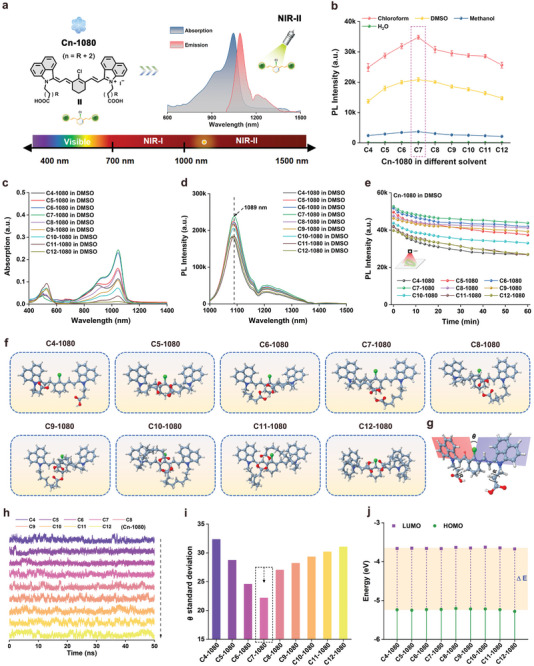
Screening and characterization of Cn‐1080 dyes with optimum NIR‐II brightness. a) Schematic structure of Cn‐1080 dyes. b) NIR‐II brightness of Cn‐1080 dyes in different solvents, including chloroform, DMSO, methanol, and H_2_O. c) UV absorption spectra, d) fluorescence spectra, and e) photostability of Cn‐1080 dyes in DMSO. f) Optimal molecular conformation of Cn‐1080 dyes with different side‐chain lengths. Schematic of the molecular torsion restriction of the side‐chain to Cn‐1080 dye. Simulation of h) molecular torsional amplitude and i) torsional angle variance of different Cn‐1080 dyes. j) HOMO and LUMO energy levels of different Cn‐1080 dyes based on density functional theory. The HOMO and LUMO energy levels were plotted based on the optimized S0 and S1 geometries using Gaussian (b31yp/6‐31g(d)).

Inspired by the structure of traditional fluorescent proteins,^[^ ^40^, ^41^ ^]^ we proposed a strategy for the in situ construction of NIR‐II FPs based on protein tagging from the biomimetic perspective. According to our previous studies on the binding ability between dye structure and exogenous albumin, the 1080 dye skeleton structure was selected as a chromophore candidate to construct in situ NIR‐II FPs (**Figure** **1**a; Figures S1 and S2, Supporting Information). Whereafter, the structure of Cn‐1080 dye was optimized by using a molecular side‐group engineering strategy in order to screen the optimal NIR‐II chromophore with excellent optical properties and highly efficient albumin‐tagging properties. As shown in Figure 1b–d and Figure S2 (Supporting Information), the NIR‐II brightness, UV absorption, and fluorescence data consistently confirmed that the length of the carboxyl side chain directly affected the optical properties of Cn‐1080 dyes. Furthermore, the C7‐1080 dye molecule exhibited the optimal NIR‐II brightness and photostability compared with other side‐chain dye molecules, which was far superior to clinically approved ICG probes (Figure 1e; Figure S3, Supporting Information).

To better understand the mechanism of luminescence regulation of Cn‐1080 dyes synthesized based on the molecular side‐group engineering strategy, we simulated the optimal conformations of different Cn‐1080 dyes and quantitatively analyzed their molecular vibration by molecular dynamics method. As shown in Figure 1f and Movie S1 (Supporting Information), the side chains of different Cn‐1080 dyes showed different optimal conformations and the two carboxyl groups tended to “hand in hand” to form intramolecular hydrogen bonds for containing the molecular oscillations. Especially, the C7‐1080 dye molecules with the most appropriate side‐chain length can minimize the end‐group swinging and reduce the occurrence of torsional intramolecular charge transfer (TICT), thus exhibiting more outstanding luminescence ability (Figure 1g–i). Meanwhile, we also calculated the highest occupied molecular orbital (HOMO) and the lowest unoccupied molecular orbital (LUMO) of different Cn‐1080 dyes using density functional theory (Figure 1j; Figure S4, Supporting Information). Gaussian calculation results showed that the side‐chain length had almost no effect on the bandgap of Cn‐1080 dyes, causing no apparent blue/red shift of the corresponding peak. The above results effectively confirmed that the mechanism of luminescence regulation of Cn‐1080 dye derived from the effective restriction of carboxyl side chain on molecular skeleton torsion. Collectively, the C7‐1080 dye was expected to be the optimum NIR‐II chromophore candidate for fluorescent protein construction.

### 2.2 Construction and Characterization of the NIR‐II Fluorescent Proteins In Vitro

**Figure 2:**
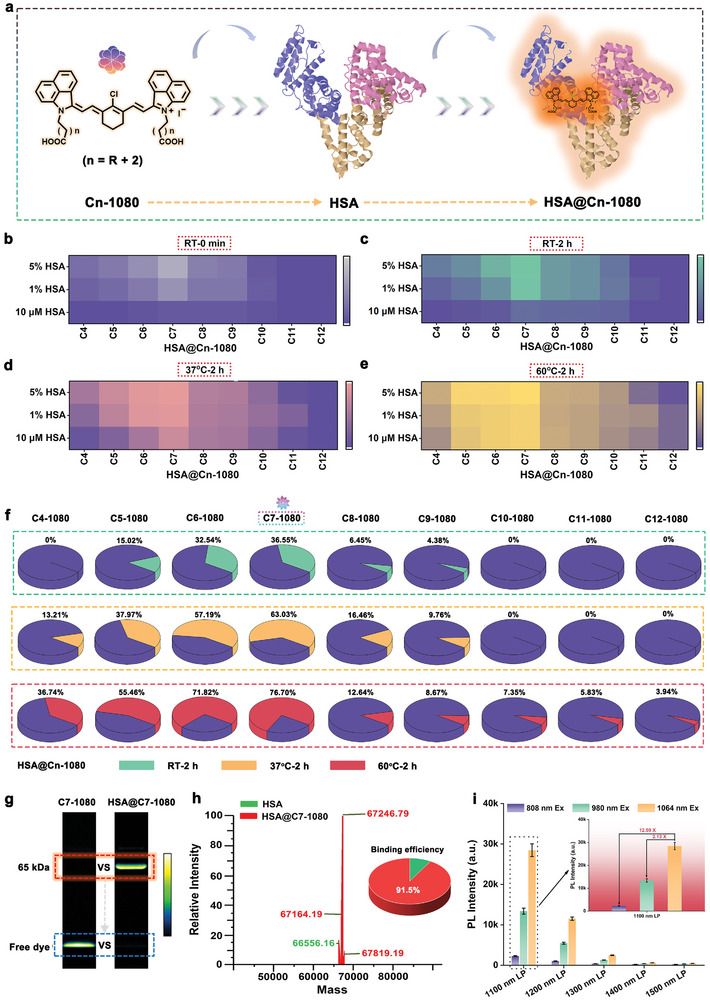
Construction and characterization of the NIR‐II fluorescent proteins based on albumin‐specific tag. a) Schematic of covalent binding of Cn‐1080 dye and HSA. Brightness optimization of Cn‐1080 dye and HSA with different concentrations under different reaction conditions, including b) RT‐0 min, c) RT‐2 h, d) 37 °C‐2 h, e) 60 °C‐2 h. f) Statistics of covalent binding efficiency of HSA and Cn‐1080 dye at different temperatures (RT, 37 °C, and 60 °C) for 2 h. g) SDS‐PAGE gel electrophoresis of C7‐1080 dye and HSA@C7‐1080 complex. h) High‐resolution mass spectrometry of HSA@C7‐1080 complex. i) Brightness comparison of HSA@C7‐1080 complex at different excitation wavelengths, including 808 nm Ex, 980 nm Ex, and 1064 nm Ex.

Indeed, our previous work has revealed that chlorinated cyanine dyes can effectively covalently embed into the hydrophobic pockets of albumin via nucleophilic substitution reactions to form bionic NIR fluorescent proteins.^[^ ^37^, ^42^, ^43^, ^44^ ^]^ Moreover, albumin as an outer shell can significantly suppress non‐radiation transitions caused by internal rotation/vibration, thereby greatly improving the brightness of the NIR dye molecules.^[^ ^43^, ^45^, ^46^ ^]^ However, the construction of these FPs often requires in vitro intervention, such as heating or adding catalysts, which are difficult to accomplish automatically in vivo. To address this challenge, we selected HSA solution as a simulated in vivo environment and tried to identify an efficient in situ albumin‐tagging dye from Cn‐1080 series dyes without external intervention, so as to realize the in vivo construction of fluorescent protein (**Figure** **2**a). According to the NIR‐II brightness and gel electrophoresis data after binding Cn‐1080 dye to HSA under various reaction conditions, we were surprised to find that C7‐1080 dyes exhibited optimal albumin‐binding capacity and fluorescence enhancement, particularly at physiological albumin concentration (5% HSA) (Figure 2b–e; Figures S5–S8, Supporting Information). Meanwhile, high‐resolution mass spectrometry (HRMS) data were employed to further analyze the covalent binding of Cn‐1080 dyes with different side‐chain lengths to HSA. Consistent with the above results, the C7‐1080 dye showed the most covalent binding affinity to albumin, surpassing other Cn‐1080 dyes (Figure 2f; Figure S9, Supporting Information).

Subsequently, the covalent binding behavior between C7‐1080 dye and HSA was analyzed in detail in vitro. For the HSA@C7‐1080 complex (at 60 °C for 2 h), almost no fluorescence band of free C7‐1080 dye was observed in gel electrophoresis data (Figure 2g). Furthermore, HRMS data confirmed that the covalent binding efficiency between HSA and C7‐1080 dyes reached up to 91.5% (Figure 2h; Figure S10, Supporting Information). Besides, the optical properties of the C7‐1080 dyes in different solutions, including DMSO, PBS, and HSA, were investigated in Figure 2i and Figures S11–S13 (Supporting Information). Results revealed that HSA could effectively endow C7‐1080 dye with brighter NIR‐II luminescence and superior photostability. The fluorescence retention (compared to dye in DMSO) and enhancement (compared to dye in PBS) of HSA@C7‐1080 complex were up to 51.11% and 346.69‐fold, respectively. Moreover, the HSA@C7‐1080 complex displayed relatively more favorable excitation properties under 1064 nm compared with 808 nm and 980 nm, with its NIR‐II luminescence extending to the 1500 nm imaging window. Taken together, these findings indicated that the C7‐1080 dye was the most ideal chromophore candidate for in situ constructing NIR‐II biomimetic FPs in vivo.

### 2.3 Analysis of Binding Behavior Between Cn‐1080 Dyes and HSA

**Figure 3:**
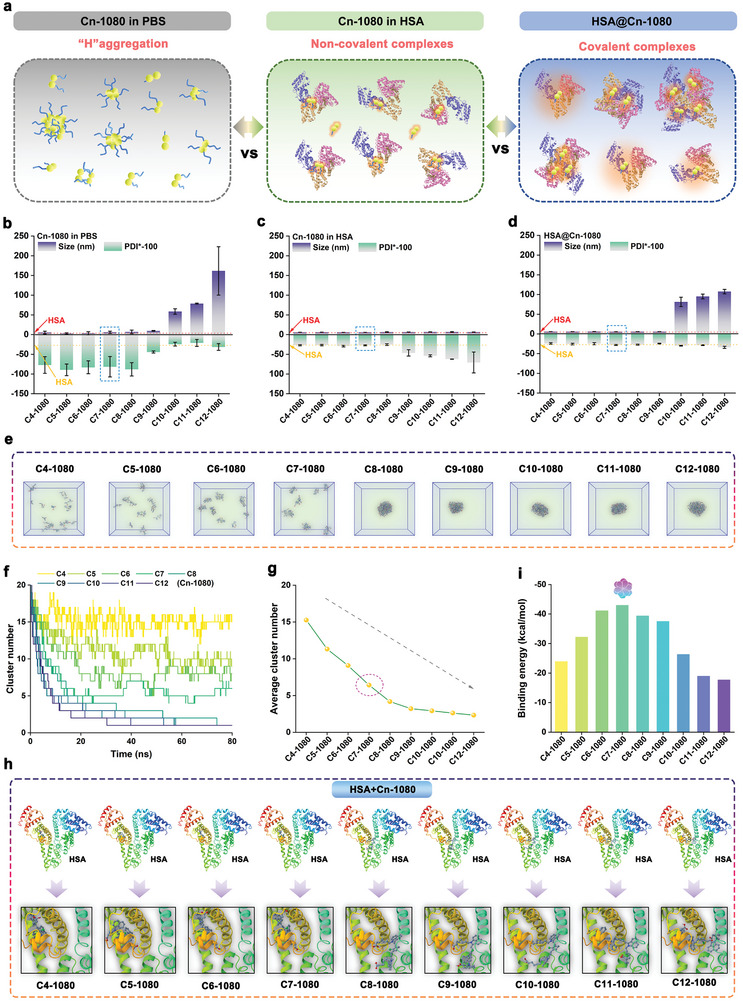
Analysis of binding behavior between different Cn‐1080 dyes and HSA. a) Solution dispersion schematic of Cn‐1080 in PBS, Cn‐1080 in HSA, and HSA@Cn‐1080. The particle size and polymer dispersity index (PDI) of b) Cn‐1080 in PBS, c) Cn‐1080 in HSA, and d) HSA@Cn‐1080. e) The molecular dynamics simulations process of different Cn‐1080 dyes in aqueous solution. f) Cluster number of different Cn‐1080 dyes during molecular dynamics simulation. g) Average cluster number statistics of different Cn‐1080 dyes during molecular dynamics simulation. h) Theoretical simulation of different Cn‐1080 dyes binding to HSA protein by gliding docking mode. i) Binding energy statistics between different Cn‐1080 dyes and HSA protein.

To clarify the mechanism of binding differences between Cn‐1080 dye molecules with different side chain lengths and HSA, DLS particle size analysis of Cn‐1080 dye under various environments was first performed (**Figure** **3**a–d; Figures S14–S16, Supporting Information). Results showed that the Cn‐1080 dye easily forms a heterogeneous system in the aqueous solution due to “H” aggregation‐induced behavior, with longer side‐chain variants showing a propensity to spontaneously assemble into large‐size nanoparticles. However, the introduction of HSA effectively regulated the aggregation behavior of pure dye molecules, with HSA rapidly forming non‐covalent complexes with short‐side‐chain Cn‐1080 dyes (n ≤ 8). Especially following a heating reaction, HSA and Cn‐1080 dyes tended to form covalent complexes. Notably, the entanglement effect of longer side chains further facilitated the formation of homogeneous and large‐size nanoparticles between the Cn‐1080 dye (n ≥ 10) and HSA after heating. This phenomenon effectively illustrates that the length of side chain plays a crucial role in regulating the binding behavior of albumin to dye molecules.

Furthermore, the differential binding mechanism between Cn‐1080 dyes with different side chain lengths and HSA protein was investigated by molecular dynamics simulation. As shown in Figure 3e–g and Movie S2 (Supporting Information), with the extension of the molecule side‐chain, Cn‐1080 dyes exhibited a greater tendency to aggregate and form clusters in the aqueous environment. This aggregation phenomenon also hindered the dye molecules from entering the protein's hydrophobic reaction pocket, thus greatly reducing their binding efficiency. Especially, Cn‐1080 dyes tended to form large clusters after 80 ns of molecular dynamics simulation when n ≥ 8. Moreover, we also conducted glide program‐based noncovalent molecular docking simulations to analyze the binding behavior between different Cn‐1080 dyes and HSA. As shown in Figure 3h and Figures S17 and S18 and Movie S3 (Supporting Information), the side‐chain length directly affected the binding position and morphology of Cn‐1080 in HSA. With increasing side chain length, Cn‐1080 gradually shifted away from the protein's hydrophobic cavity, particularly evident when n ≥ 8. Remarkably, quantitative binding analysis indicated that HSA exhibited the highest binding energy with C7‐1080 dye, facilitating the disruption of the dye cluster structure more effectively, and thereby enhancing covalent binding efficiency and NIR‐II fluorescence (Figure 3h). All in all, the C7‐1080 dye possessed optimal affinity behavior for albumin, making it a preferred choice as an albumin tag for in situ construction of NIR‐II fluorescent proteins.

### 2.4 Analysis of Specific Covalent Binding Mechanism Between C7‐1080 Dyes and HSA

**Figure 4:**
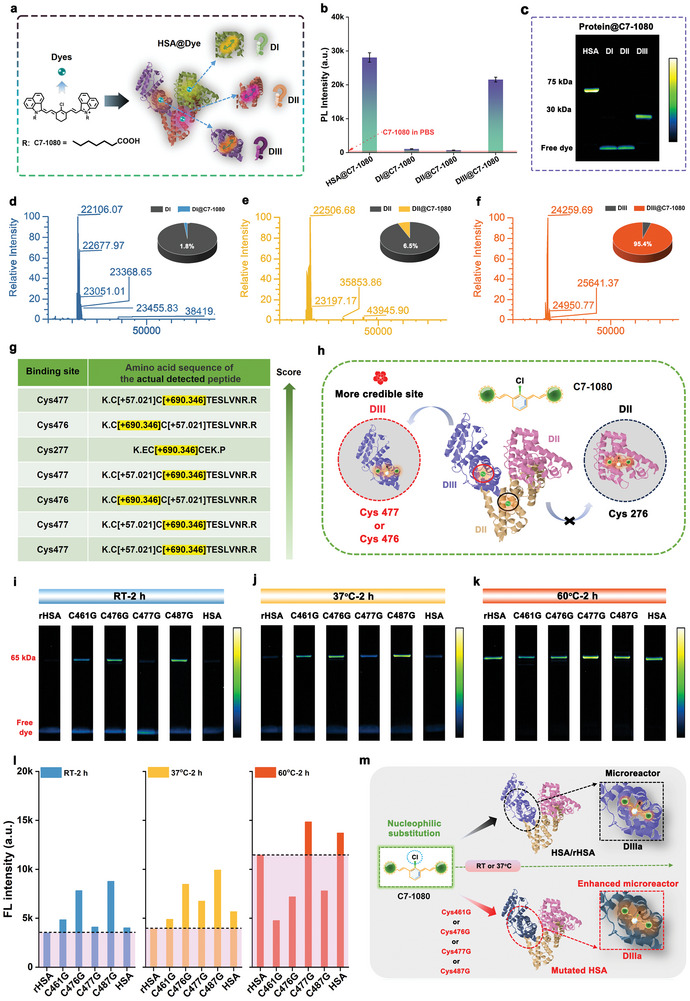
Analysis of specific binding mechanism between C7‐1080 dyes and HSA. a) Schematic illustration of the binding domain between C7‐1080 dye and HSA. b) Brightness and c) gel electrophoresis analysis of the binding ability between C7‐1080 and HSA, DI, DII, DIII. High‐resolution mass spectrometry of d) DI@C7‐1080, e) DII@C7‐1080, and f) DIII@C7‐1080. g) Peptide information and specific binding sites containing C7‐1080 dye obtained by proteomic analysis. h) Schematic of specific binding behavior between the C7‐1080 dye and HSA. Gel electrophoresis analysis of C7‐1080 dyes with different site mutations under different reaction conditions, including i) RT‐2 h, j) 37 °C‐2 h, and k) 60 °C‐2 h. l) Fluorescence band brightness analysis of recombinant HSA and C7‐1080 dyes with different site mutations under different reaction conditions, including RT‐2 h, 37 °C‐2 h, and 60 °C‐2 h. m) Reaction mechanism diagram of C7‐1080 with HSA/rHSA and mutated HSA.

Following the determination of the optimal chromophore protocol for in situ biomimetic NIR‐II FPs construction, we further explored in detail the specific binding domain between the HSA and C7‐1080 chromophore. HSA is known to consist of three domains, namely domain I (DI), domain II (DII), and domain III (DIII).^[^ ^47^, ^48^ ^]^ Therefore, to identify the specific reaction domain with HSA, recombinant DI, DII, and DIII proteins were reacted with C7‐1080 chromophore (**Figure** **4**a). NIR‐II brightness, gel electrophoresis, and HRMS data consistently confirmed that C7‐1080 dye mainly covalently bound to the DIII domain of HSA, showing optimal fluorescence enhancement and covalently binding efficiency of up to 95.4% (Figure 4b–f; Figure S19, Supporting Information). Conversely, DI and DII exhibited minimal fluorescence enhancement and covalent binding capacity, with binding efficiencies of 1.8% and 6.8%, respectively.

To identify specific covalent binding sites between C7‐1080 chromophore and HSA, as well as to better understand the reaction mechanism, we performed an unbiased shotgun proteomics analysis for the HSA@C7‐1080 complex (Figure S20a, Supporting Information). The above complexes were first digested by chymotrypsin and trypsin, cutting specific cleavage sites during the process (Figure S21, Supporting Information). Then the resulting peptides were analyzed by ultra‐performance liquid chromatography with tandem mass spectrometry (Nano LC‐MS/MS; Figures S22–S24, Supporting Information). As shown in Figure 4g and Figures S20b and S25 (Supporting Information), the detailed proteomic analysis identified seven distinct peptide sequences with staining markers corresponding to HSA protein residues Cys277, Cys476, and Cys477. Combining this information with previous covalent binding domain validation results, Cys277 binding sites could be effectively excluded. Taken together, the potentially credible covalent binding sites between HSA and C7‐1080 dye could be Cys467 and/or Cys477 residues in the DIIIa subdomain (Figure 4h). Among these, the peptide containing the staining marker for Cys477 residue was considered a more likely binding site due to its higher score.

Based on the proteomic identification results, we carried out a secondary verification of the binding site between C7‐1080 and HSA using rational protein mutation techniques. Herein, we first selected Cys476 and Cys477 and their corresponding disulfide (‐S‐S‐) binding sites Cys487 and Cys461 as mutation sites, and successfully synthesized a series of site‐mutated proteins (Figures S26 and S27, Supporting Information). Afterward, the obtained site‐mutated HSA were reacted with C7‐1080 dye under different conditions. As shown in Figure 4i–l and Figure S28 (Supporting Information), gel electrophoresis and NIR‐II brightness data confirmed that the Cys477 site‐mutated HSA severely impeded the binding between protein and dye compared with other site mutations, especially at room temperature (RT) or 37 °C. Besides, the careful comparison revealed that site‐mutated HSA could act as an enhanced microreactor in comparison to wild‐type HSA or recombinant HSA (rHSA), effectively improving the covalent binding efficiency and fluorescence amplification effect between C7‐1080 dyes and proteins (Figure 4m). We speculate that the disruption of disulfide bonds caused by mutations enhances the reactivity between the dye and the corresponding cysteine residues. Collectively, the above results reaffirmed that the preferred binding site between C7‐1080 and HSA was Cys477. Unexpectedly, the effective implementation feedback of protein mutation technology in this work also provided a new idea for the subsequent design of protein shells that better accommodate the target chromophore.

Furthermore, after successful validation of covalent binding sites between HSA and C7‐1080, we sought to visualize their interactions and binding poses through covalent docking simulations. Indeed, the simple manual‐screened molecular covalent docking conformations were usually unstable. Therefore, we subsequently performed a molecular dynamics (MD) simulation analysis for the above‐screened covalent docking results (0 ns) using the Desmond module to optimize the eventually stable HSA@C7‐1080 conformation (Figure S29a; Movie S4, Supporting Information). The RMSD results indicated that HSA and C7‐1080 essentially reached a stable state after 100 ns (Figure S29b,c, Supporting Information). After comprehensive consideration, MD simulation results of 120 ns were selected as the final covalent‐binding conformation. Combined with the previous non‐covalent simulation results, the non‐covalent docking conformation and the steady‐state binding conformation of covalent docking between HSA and C7‐1080 were also carefully analyzed in Figure S30 (Supporting Information). Results showed that the covalently bound C7‐1080 dye exhibited more amino acid interactions with the HSA skeleton and higher binding energy compared to non‐covalent binding, thus more effectively limiting the self‐torsion of dye molecules. Overall, the binding process between C7‐1080 dyes and HSA could be divided into three successive stages: In Stage I, the C7‐1080 dye was inserted into the hydrophobic pocket of HSA, binding to the pocket through supramolecular interactions, and effectively enhancing its NIR‐II luminescence. In Stage II, the Cl‐C group of C7‐1080 dye and the ‐SH group of Cys477 on the HSA protein skeleton formed covalent bonds through nucleophilic substitution reaction. In Stage III, the covalent conformation between the C7‐1080 dye and HSA was fine‐tuned to eventually form a relatively more steady‐state bright‐emitting NIR‐II FPs, with its NIR‐II luminescent ability further amplified. Among them, it is noteworthy that Stage III might occurred simultaneously with Stage II.

### 2.5 In Vivo Albumin‐Tagging Imaging of Cn‐1080 Dye

**Figure 5:**
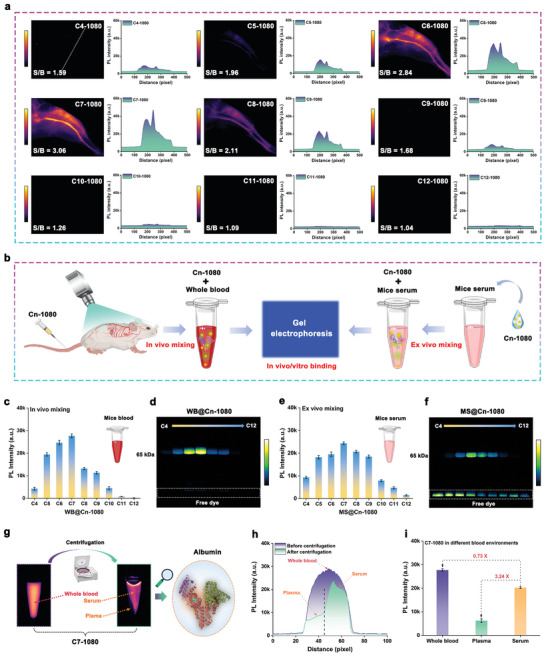
In vivo albumin‐tagging imaging behavior of Cn‐1080 dye. a) Comparison of vascular imaging ability of Cn‐1080 dyes with different side chain lengths. b) Schematic diagram of in vitro/vivo binding behavior of Cn ‐1080 dyes with different side chain lengths. c) Brightness, d) gel electrophoresis analysis of mouse blood after tail vein injection of Cn ‐1080 dye. e) Brightness and f) gel electrophoresis analysis of Cn‐1080 dye mixed with mouse serum in vitro. g) Schematic of the binding behavior of C7‐1080 to mouse blood. h) Fluorescence signal analysis and i) brightness comparison of each component of C7‐1080 mixed with mouse blood.

Considering their potential clinical application, it was necessary to evaluate the biosafety of C7‐1080 dyes. As shown in Figure S31 (Supporting Information), the blood routine, liver function, and renal function data from mice injected with C7‐1080 dye confirmed that the C7‐1080 dye possessed reasonable biosafety in vivo. Based on the above in vitro albumin‐tagging and biosafety results, we proceeded to investigate the in vivo albumin‐tagging behavior of Cn‐1080 dye in detail. As shown in **Figure** **5**a and Figures S32–S34 (Supporting Information), both angiography and in vivo metabolic imaging demonstrated that C7‐1080 dye exhibited a more prominent fluorescence signal as well as a signal‐to‐noise ratio after tail‐vein injection compared to other Cn‐1080 series dyes. Meanwhile, the metabolic data also confirmed that Cn‐1080 series dyes possessed excellent in vivo excretion capacity, with almost no fluorescence signal residue 72 h after tail vein injection.

To understand the reasons for the differences in in vivo imaging, we further analyzed the binding behavior between Cn‐1080 and whole blood (WB) as well as mouse serum (MS) (Figure 5b). As shown in Figure 4c–f, similar to our in vitro results, both NIR‐II brightness and gel electrophoresis data of WB@Cn‐1080 and MS@Cn‐1080 complexes indicated that C7‐1080 displayed the optimal albumin fluorescence tagging ability in vivo. In addition, as shown in Figure 5g, the centrifugation analysis of whole blood from mice injected with C7‐1080 dye revealed that it preferentially covalently bound to albumin in serum rather than plasma, thus forming NIR‐II fluorescent proteins in situ (Figure 5h,i). Overall, these investigations demonstrated that C7‐1080 could be preferred as a fluorescent tag of albumin for in situ construction of fluorescent proteins.

### 2.6 NIR‐II Targeted Imaging of Blood‐Brain Barrier Disruption by In Situ NIR‐II Fluorescent Proteins

The blood‐brain barrier (BBB) is characterized by a highly selective permeability between blood and brain parenchyma. Normally, albumin is scarce in brain tissue. However, after a stroke/trauma event, blood can penetrate the brain parenchyma through the damaged BBB, resulting in a surge in local albumin levels.^[^ ^49^, ^50^ ^]^ Currently, there have been relevant reports using endogenous albumin in the brain serves as a biomarker of BBB injury, and attempts to visually assess the BBB integrity by optical imaging techniques.^[^ ^51^, ^52^ ^]^ Therefore, the development of NIR‐II probes with specific in situ binding to endogenous albumin holds promise for providing better imaging tools for monitoring BBB disruption.

**Figure 6:**
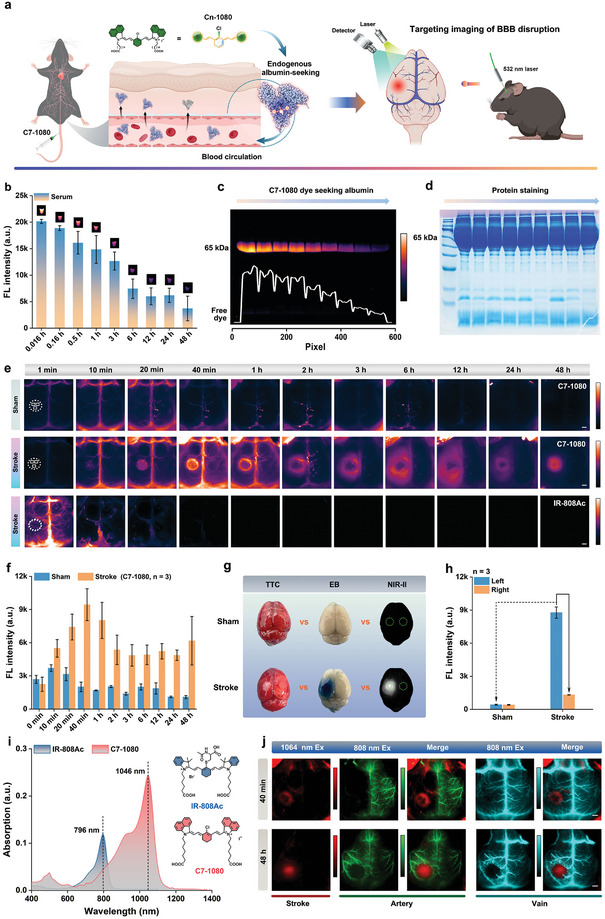
Targeted imaging of blood‐brain barrier (BBB) disruption using the in situ albumin‐tagging dye. a) Schematic of BBB disruption imaging based on C7‐1080 dye. b) Blood brightness, c) gel electrophoresis, and d) protein staining analysis at different time points after tail vein injection of C7‐1080 dye. e) Dynamic imaging of sham ^−1^stroke mice (532 nm irradiation for 10 min) at different time points after tail vein injection of C7‐1080 and IR‐808Ac dyes (*n* = 3 independent mice). f) Statistical analysis of fluorescence signals in ROI regions of sham ^−1^stroke mice at different time points after intravenous injection of C7‐1080. g) 2,3,5‐triphenyl tetrazolium chloride (TTC) staining (48 h after intravenous injection of C7‐1080), Evans blue (EB) staining (3 h after intravenous injection of C7‐1080), and NIR‐II fluorescence imaging (3 h after intravenous injection of C7‐1080) of the ex‐vivo whole brain of sham and stroke mice. h) Statistical analysis of fluorescence signals in left and right brain ROI regions of sham and stroke mice (*n* = 3 independent mice, 3 h after intravenous injection of C7‐1080). i) UV absorption spectra and structural formula of IR‐808Ac and C7‐1080 dyes. j) Dual‐channel images of the BBB disruption and cerebral vascular network based on the IR‐808Ac and C7‐1080 dyes (*n* = 3 independent mice). Scale bar = 1 mm.

Thanks to the established reliable interaction between the C7‐1080 dye and albumin, it became feasible not only to directly visualize albumin leakage, but also potentially to pinpoint BBB disruption during stroke. As shown in **Figure** **6**a, we successfully established a photothrombotic stroke (PTS) model with a similar infarct area in the left cerebral cortex of mice.^[^ ^53^, ^54^, ^55^, ^56^, ^57^ ^]^ Before model imaging, blood was first collected from mice intravenously injected with C7‐1080 dye to validate the formation and pharmacokinetics of NIR‐II FPs in vivo. As shown in Figure 6b–d, the blood brightness, gel electrophoresis, and protein staining effectively confirmed the formation of fluorescent protein in vivo, with a sustained in vivo circulation period, thereby laying a preliminary foundation for targeted imaging of BBB disruption.

Subsequently, we applied C7‐1080 as the in situ albumin‐tagging dye to conduct BBB disruption imaging studies. As shown in Figure 6e,f, fluorescence signals gradually accumulated in the stroke area, with leakage detected as early as 10 min compared with the sham group, indicating their infiltration into the brain parenchyma through the impaired BBB. Conversely, the albumin‐escaping NIR probes (IR‐808Ac) failed to image BBB disruption in stroke due to its rapid hepatic clearance and short circulation time without covalent binding with albumin (Figure 6e).^[^ ^58^ ^]^ In addition, we modeled different degrees of stroke by modulating the photothrombotic time to assess the sensitivity of C7‐1080 dye for monitoring BBB disruption (Figure S35a,b, Supporting Information). Encouragingly, the C7‐1080 dyes still appeared excellent BBB disruption detection in mild‐symptom stroke models (photothrombotic for 1 min). We also compared the NIR‐II BBB‐disruption imaging technique with commonly used PTS model characterization methods such as 2,3,5‐triphenyl tetrazolium chloride (TTC, brain injury assessment probe) staining and Evans blue (EB, BBB leakage assessment probe) staining. As shown in Figure 6g,h and Figures S35c, S36–S38 (Supporting Information), the TTC and EB staining region was basically consistent with the NIR‐II fluorescence imaging region. These results indicated that cerebral ischemic stroke based on the PTS method caused BBB injury/disruption, resulting in extravasation of plasma components into the brain parenchyma. Notably, we also found that the in‐vitro constructed fluorescent protein (HSA@C7‐1080) did not display ideal BBB disruption imaging capability after tail‐vein injection (Figures S35d and S39, Supporting Information, the reason needs further investigation). The above phenomena effectively indicated that the prominent in situ albumin‐tagging ability of C7‐1080 dye was the direct reason for its realization of sensitive and specific imaging of BBB disruption.

To further understand the changes in blood pathways around the BBB‐damaged area, we selected another albumin‐escaping NIR probe (IR‐808Ac) with the optimal excitation at 808 nm for cerebrovascular visualization in stroke mice (note: we can use tail emission of IR‐808Ac for high‐quality NIR‐II imaging; Figure 6i and Figures S40 and S44a, Supporting Information).^[^ ^42^, ^58^ ^]^ We subsequently captured NIR‐II images of BBB leakage (1064 nm excitation, C7‐1080) and dynamic changes in the cerebrovascular system (808 nm excitation, IR‐808Ac) under two different imaging channels, respectively (Figure 6j). Results showed that IR‐808Ac could not only clearly and dynamically delineate the changes and recovery of the blood routes during ischemic stroke, but also distinguish the arteriovenous vessels in real‐time (Figure 6j; Figures S41–S43, Supporting Information). Notably, dual‐channel imaging revealed that almost no blood vessels were illuminated in the BBB‐damaged area. The real‐time angiography after stroke showed a transient loss of blood supply near the injury site followed by recovery, and the ischemic area was gradually localized to the injury area over time (Figures S42 and S43, Supporting Information). Meanwhile, the dual‐channel imaging also effectively indicated that the essence of ischemic stroke imaging was achieved by the tagged‐albumin flowing through the BBB disruption region, leaking from the blood vessels to the brain parenchyma (Figure S44b, Supporting Information).

## 3 Conclusion 

In summary, based on the molecular side group engineering strategy, we conceptually validated and proposed a class of dye molecule (Cn‐1080) with bright NIR‐II luminescence and albumin‐specific covalent labeling capabilities as NIR‐II chromophore for in situ construction of fluorescent proteins. In vitro/vivo experiments demonstrated that the C7‐1080 chromophore could achieve site‐specific covalent binding with albumin (such as HSA) through nucleophilic substitution reaction without any additional assistance to form NIR‐II FPs in situ. Notably, as a fluorescent protein shell, the HSA acts as a brightness amplifier and stability regulator for chromophores through the tight clamping effect. Theoretical simulation, proteomics, and protein mutation techniques were employed to thoroughly investigate the binding behavior between albumin and chromophore, and the specific binding site was identified as Cys 477 on the DIIIa domain. More importantly, in situ NIR‐II FPs generated by the reliable interaction between the chromophore and albumin not only provided a direct visualization of albumin leakage but also enabled high‐precision imaging of BBB disruption during ischemic stroke. The real‐time dual‐channel imaging of BBB disruption and cerebral vessels was also realized through the effective combination with the albumin‐escaping IR‐800Ac probe, offering insights into the mechanisms of ischemic stroke. Overall, the proposed in situ fluorescent protein construction strategy is expected to address important gaps in the current limitations of NIR‐II FPs and open up new opportunities for the study of stroke‐related diseases.

## Conflict of Interest

The authors declare no conflict of interest.

## Ethical Statement

All animal experiments in this work were conducted under institutional guidelines and were approved by the Animal Ethical Committee of The First Hospital of Jilin University (Protocol number: 20 210 642).

## Supporting information



Supporting Information

Supplemental Movie 1

Supplemental Movie 2

Supplemental Movie 3

Supplemental Movie 4

## Data Availability

The data that support the findings of this study are available from the corresponding author upon reasonable request.
