# Supplementary material for: NIR‐II Fluorescent Protein Created by In Situ Albumin‐Tagging for Sensitive and Specific Imaging of Blood‐Brain Barrier Disruption
Source: Adv Sci (Weinh). 2025 Feb 25;12(16):2500443. doi: 10.1002/advs.202500443 (PMC12021084; doi:10.1002/advs.202500443)
Supplement: Supplementary file 1 — Supporting Information [file ADVS-12-2500443-s002.docx]

**Supporting Information**

**NIR-II Fluorescent Protein Created by In-situ Albumin-tagging for Sensitive and Specific Imaging of Blood-brain Barrier Disruption**

*Jiajun Xu,* Yijing Du, Ningning Zhu, Jia Li,* Yuewei Zhang, Ding Zhou,* Shoujun Zhu**

**Methods**

**Materials**

Human serum albumin (HSA, ≥ 98%) was purchased from Sigma-Aldrich. 2,3,5-Triphenyl tetrazolium chloride (TTC, ≥ 98.0%) and Evans Blue (EB, ≥ 85%) were purchased from Beijing Solarbio Science & Technology Co. Ltd. ICG (modified) for human injection was purchased from Dandong Yichuang Pharmaceutical Co. Ltd. Recombinant human albumin domain I (DI, ≥ 95%), recombinant human albumin domain II (DII, ≥ 95%), and recombinant human albumin domain III (DIII, ≥ 95%) were purchased from Albumin Therapeutics LLC. Benzo[*cd*]indol-2(1*H*)-one, phosphorus pentasulfide, pyridine, sodium hydroxide (NaOH), methyl iodide, acetone, methanol, 2,2-dimethyl-1,3-dioxane-4,6-dione, ethanol, triethylamine, ethyl iodide, potassium carbonate (K_2_CO_3_), concentrated hydrochloric acid (HCl), potassium iodide, acetic acid, acetic anhydride, 1-iodopropane, 1,4-butylenesulfone, N-methylpyrrolidone (NMP), 2,6-di-tert-butyl-4-methyl pyridine (DTBMP), 1-butanol, and ethyl n-bromohexanoate (n = 4~12) were purchased from Energy Chemical. Unless otherwise noted, all reagents were obtained from commercial resources and used without further purification. Tetrahydrofuran (THF), toluene, and dimethyl formamide (DMF) used for reactions were purified by fully stirring with Na and using benzophenone as the indicator and then evaporated for use. All air and moisture-sensitive reactions were carried out in flame-dried glassware under a nitrogen atmosphere.

**Characterization**

UV absorption spectra of different probes were measured by a LAMBDA 1050+ spectrophotometer. Fluorescence spectra of different probes were measured using Edinburgh instrument FLS920 fluorescence spectrophotometer. Sodium dodecyl sulfate-polyacrylamide gel electrophoresis (SDS-PAGE) of different probes was carried out using an American BIO-RAD electrophoresis system. The covalent binding behavior of protein to different dyes was characterized by Orbitrap Eclipse high-resolution mass spectrometer. The particle sizes of different samples were measured by a Malvern Zetasizer Nano ZS size analyzer. All ^1^H spectra and ^13^C spectra were performed on Bruker AVANCE III 400 MHz and AVANCE III 600 MHz NMR spectrometers. Chemical shifts were reported in ppm relative to tetramethylsilane (TMS) peak or the residual solvent peak (CDCl_3_: ^1^H, 7.26).

**Synthesis of Cn-1080 dyes**

1080-series cyanine dyes with different side chain lengths (named Cn-1080) were synthesized according to our previously reported methods.^[1]^ The specific synthesis steps were shown in **Figure S1**.


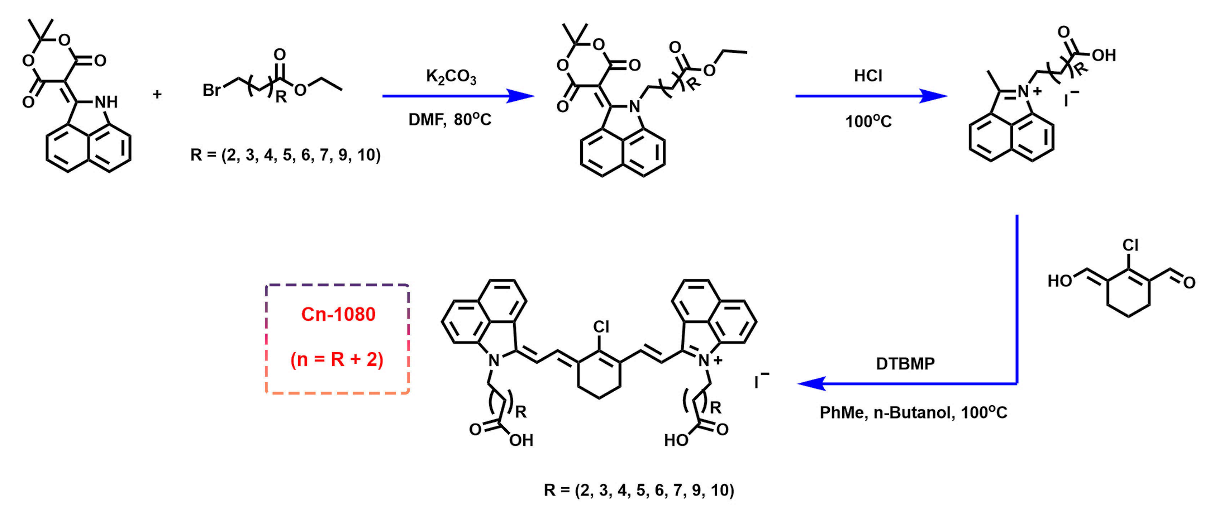


**Figure S1.** Synthesis route schematic of Cn-1080 dye.

**C4-1080:** ^1^H-NMR (400 MHz, DMSO-d6, ppm) *δ* 8.38 (d, *J* = 13.7 Hz, 2H), 8.08 (d, *J* = 7.7 Hz, 2H), 8.00 (d, *J* = 8.0 Hz, 2H), 7.81 (t, *J* = 7.7 Hz, 2H), 7.54 – 7.29 (m, 6H), 6.66 (d, *J* = 14.0 Hz, 2H), 4.24 – 3.97 (m, 4H), 2.95 – 2.69 (m, 4H), 2.43 (t, *J* = 6.6 Hz, 4H), 2.02 – 1.73 (m, 6H). LC-HRMS (ESI-TOF): calcd. For C_40_H_36_ClN_2_O_4_^+^ [M]^+^ 643.2358; found 643.2338.

^13^C NMR (100 MHz, DMSO-d6) δ 174.58, 152.95, 147.60, 141.25, 140.48, 131.75, 131.55, 130.31, 129.58, 128.95, 128.00, 124.50, 123.19, 110.53, 108.08, 43.19, 30.65, 26.29, 24.21, 20.88.

**C5-1080:** ^1^H-NMR (400 MHz, DMSO-d6, ppm) *δ* 8.53 (d, *J* = 13.9 Hz, 2H), 8.24 (d, *J* = 7.6 Hz, 2H), 8.13 (d, *J* = 8.1 Hz, 2H), 7.88 (t, *J* = 7.7 Hz, 2H), 7.61 (d, *J* = 7.9 Hz, 2H), 7.58 – 7.45 (m, 4H), 6.71 (d, *J* = 14.1 Hz, 2H), 4.23 – 4.20 (m, 4H), 2.90 – 2.78 (m, 4H), 2.30 (t, *J* = 7.2 Hz, 5H), 2.00 – 1.70 (s, 2H), 1.84 –1.71 (m, 4H), 1.69 – 1.57 (m, 4H). LC-HRMS (ESI-TOF): calcd. For C_42_H_40_ClN_2_O_4_^+^ [M]^+^ 671.2671; found 671.2588.

^13^C NMR (100 MHz, DMSO-d6) δ 174.77, 153.09, 147.51, 141.30, 140.80, 131.91, 131.70, 130.39, 129.71, 129.19, 127.99, 124.57, 123.43, 110.91, 108.29, 43.81, 33.68, 28.57, 26.39, 22.30, 20.91.

**C6-1080:** ^1^H-NMR (400 MHz, DMSO-d6, ppm) *δ* 12.03 (s, 1H), 8.55 (d, *J* = 13.9 Hz, 2H), 8.26 (d, *J* = 7.7 Hz, 2H), 8.15 (d, *J* = 8.1 Hz, 2H), 7.89 (t, *J* = 7.7 Hz, 2H), 7.64 (d, *J* = 8.1 Hz, 2H), 7.55 (t, *J* = 7.7 Hz, 2H), 7.49 (d, *J* = 7.3 Hz, 2H), 6.71 (d, *J* = 14.2 Hz, 2H), 4.38 – 4.12 (m, 4H), 2.89 – 2.77 (m, 4H), 2.21 (t, *J* = 7.3 Hz, 4H), 2.00 – 1.88 (m, 2H) 1.79 – 1.71 (m, 4H), 1.57 (p, *J* = 7.3 Hz, 4H), 1.46 – 1.38 (m, 4H). LC-HRMS (ESI-TOF): calcd. For C_44_H_44_ClN_2_O_4_^+^ [M]^+^ 699.2984; found 699.3014.

^13^C NMR (100 MHz, DMSO-d6) δ 174.86, 153.02, 147.44, 141.20, 140.76, 131.86, 131.69, 130.36, 129.72, 129.19, 127.93, 124.56, 123.46, 110.90, 108.28, 43.93, 34.05, 28.88, 26.36, 26.28, 24.67, 20.86.

**C7-1080:** ^1^H-NMR (400 MHz, DMSO-d6, ppm) *δ* 12.07 (s, 1H), 8.53 (d, *J* = 13.9 Hz, 2H), 8.25 (d, *J* = 7.6 Hz, 2H), 8.14 (d, *J* = 8.1 Hz, 2H), 7.88 (t, *J* = 7.7 Hz, 2H), 7.63 (d, *J* = 8.1 Hz, 2H), 7.54 (t, *J* = 7.7 Hz, 2H), 7.48 (d, *J* = 7.3 Hz, 2H), 6.69 (d, *J* = 14.1 Hz, 2H), 4.21 (t, *J* = 7.3 Hz 4H), 2.90 – 2.72 (m, 4H), 2.20 (t, *J* = 7.3 Hz, 4H), 2.02 – 1.87 (m, 2H), 1.80 – 1.64 (s, 4H), 1.52 – 1.45 (m, 4H), 1.43 – 1.27 (m, 8H). LC-HRMS (ESI-TOF): calcd. For C_46_H_48_ClN_2_O_4_^+^ [M]^+^ 727.3297; found 727.3298.

^13^C NMR (100 MHz, DMSO-d6) δ 174.95, 153.20, 147.45, 141.31, 140.92, 131.97, 131.83, 130.48, 129.85, 129.36, 128.06, 124.65, 123.59, 111.04, 108.39, 44.02, 34.03, 28.92, 28.67, 26.43, 24.84, 20.88.

**C8-1080:** ^1^H-NMR (400 MHz, DMSO-d6, ppm) *δ* 12.01 (s, 1H), 8.57 (d, *J* = 13.0 Hz, 2H), 8.29 (d, *J* = 6.9 Hz, 2H), 8.17 (d, *J* = 7.4 Hz, 2H), 7.89 (t, *J* = 7.6 Hz, 2H), 7.66 (d, *J* = 8.0 Hz, 2H), 7.56 (d, *J* = 8.4 Hz, 2H), 7.52 (d, *J* = 7.2 Hz, 2H), 6.73 (d, *J* = 13.7 Hz, 2H), 4.37 – 4.14 (m, 4H), 2.97 – 2.73 (m, 4H), 2.30 – 2.23 (m, 2H), 2.22 – 2.10 (m, 4H), 2.08 – 1.85 (m, 4H), 1.84 – 1.63 (m, 4H), 1.60 – 1.42 (m, 8H). LC-HRMS (ESI-TOF): calcd. For C_48_H_52_ClN_2_O_4_^+^ [M]^+^ 755.3610; found 755.3525.

^13^C NMR (155 MHz, DMSO-d6) δ 174.94, 153.14, 147.41, 141.25, 140.86, 131.88, 131.77, 130.44, 129.79, 129.30, 128.03, 124.61, 123.54, 111.00, 108.33, 43.99, 34.06, 29.01, 28.94, 28.87, 26.54, 26.40, 24.90, 20.83.

**C9-1080:** ^1^H-NMR (400 MHz, DMSO-d6, ppm) *δ* 8.65 (d, *J* = 14.2 Hz, 2H), 8.38 (d, *J* = 7.5 Hz, 2H), 8.24 (d, *J* = 8.2 Hz, 2H), 7.94 (t, *J* = 7.6 Hz, 2H), 7.76 (d, *J* = 8.1 Hz, 2H), 7.64 (t, *J* = 7.7 Hz, 2H), 7.58 (d, *J* = 7.3 Hz, 2H), 6.82 (d, *J* = 14.5 Hz, 2H), 4.38 – 4.29 (m, 4H), 2.92 – 2.83 (m, 4H), 2.17 (t, *J* = 7.3 Hz, 4H), 2.06 – 1.87 (m, 4H), 1.85 – 1.67 (m, 4H), 1.59 – 1.43 (m, 8H), 1.42 – 1.35 (m, 4H), 1.36 – 1.29 (m, 6H). LC-HRMS (ESI-TOF):calcd. for C_50_H_56_ClN_2_O_4_^+^ [M]^+^ 783.3923; found 782.9720.

^13^C NMR (100 MHz, DMSO-d6) δ 174.98, 153.34, 141.40, 141.03, 131.91, 130.56, 129.92, 129.47, 128.17, 124.72, 123.70, 111.17, 108.53, 44.11, 34.17, 29.20, 29.07, 26.68, 24.97.

**C10-1080:** ^1^H-NMR (400 MHz, DMSO-d6, ppm) *δ* 8.58 (d, *J* = 13.9 Hz, 2H), 8.31 (d, *J* = 7.3 Hz, 2H), 8.18 (d, *J* = 8.0 Hz, 2H), 7.90 (t, *J* = 7.6 Hz, 2H), 7.68 (d, *J* = 8.1 Hz, 2H), 7.63 – 7.49 (m, 5H), 6.74 (d, *J* = 14.1 Hz, 2H), 4.40 – 4.16 (m, 4H), 2.93 – 2.75 (m, 4H), 2.17 (t, *J* = 7.3 Hz, 4H), 2.00 – 1.87 (m, 2H), 1.83 – 1.65 (m, 4H), 1.59 – 1.42 (m, 8H), 1.40 – 1.27 (m, 16H). LC-HRMS (ESI-TOF): calcd. For C_52_H_60_ClN_2_O_4_^+^ [M]^+^ 811.4236; found 811.4227.

^13^C NMR (100 MHz, DMSO-d6) δ 174.98, 153.10, 147.44, 141.23, 140.84, 131.77, 130.42, 129.78, 129.26, 128.01, 124.60, 123.54, 110.99, 108.26, 44.04, 34.16, 29.36, 29.24, 29.13, 29.05, 26.67, 26.41, 25.00, 20.88.

**C11-1080:** ^1^H-NMR (400 MHz, DMSO-d6, ppm) *δ* 12.08 (s, 1H), 8.56 (d, *J* = 13.8 Hz, 2H), 8.29 (d, *J* = 7.6 Hz, 2H), 8.16 (d, *J* = 8.1 Hz, 2H), 7.89 (t, *J* = 7.6 Hz, 2H), 7.66 (d, *J* = 8.0 Hz, 2H), 7.57 (t, *J* = 7.6 Hz, 2H), 7.52 (d, *J* = 7.3 Hz, 2H), 6.73 (d, *J* = 14.0 Hz, 2H), 4.32 – 4.21 (m, 4H), 2.95 – 2.72 (m, 4H), 2.17 (t, *J* = 7.3 Hz, 4H), 2.02 – 1.86 (m, 2H)), 1.82 – 1.64 (m, 4H), 1.56 – 1.41 (m, 8H), 1.40 – 1.25 (m, 20H). LC-HRMS (ESI-TOF): calcd. for C_54_H_64_ClN_2_O_4_^+^ [M]^+^ 839.4549; found 839.0310.

^13^C NMR (100 MHz, DMSO-d6) δ 174.96, 153.31, 147.36, 141.36, 140.99, 132.00, 131.90, 130.52, 129.89, 129.41, 128.12, 124.70, 123.65, 111.16, 108.50, 44.07, 34.17, 30.23, 29.43, 29.36, 29.26, 29.15, 29.08, 28.96, 26.67, 26.47, 24.99.

**C12-1080:** ^1^H-NMR (400 MHz, DMSO-d6, ppm) *δ* 11.93 (s, 1H), 8.61 (d, *J* = 13.9 Hz, 2H), 8.34 (d, *J* = 7.5 Hz, 2H), 8.21 (d, *J* = 8.1 Hz, 2H), 7.92 (t, *J* = 7.7 Hz, 2H), 7.72 (d, *J* = 8.0 Hz, 2H), 7.62 (t, *J* = 7.7 Hz, 2H), 7.56 (d, *J* = 7.3 Hz, 2H), 6.79 (d, *J* = 14.1 Hz, 2H), 4.39 – 4.21 (m, 4H), 2.91 – 2.77 (m, 4H), 2.15 (t, *J* = 7.3 Hz, 4H), 2.03 – 1.88 (m, 2H), 1.84 – 1.67 (m, 4H), 1.52 – 1.41 (m, 8H), 1.40 – 1.23 (m, 24H). LC-HRMS (ESI-TOF): calcd. for C_56_H_68_ClN_2_O_4_^+^ [M]^+^ 867.4863; found 867.1360.

**Synthesis of protein@Cn-1080** **complexes**

In order to investigate the in-situ albumin labeling ability of Cn-1080 dyes, a series of protein@dye complexes were constructed in vitro. All protein@dye complexes were synthesized using a similar protocol, with the HSA@C7-1080 probe being taken as an example here. First, 2 mM C7-1080 and 10 μM HSA were prepared in anhydrous dimethyl sulfoxide (DMSO) and PBS solutions, respectively. Subsequently, 5 μL of 2 mM C7-1080 dye was added into 1 mL of 10 μM HSA solution, and the mixture was thoroughly mixed by rapid vortices (maintaining a 1:1 molar ratio of protein and dyes). Finally, the mixture was then reacted at a set temperature (RT, 37^o^C, and 60^o^C) in a shaker for a period of time to obtain the HSA@C7-1080 complex probe. Notably, the above-obtained HSA@C7-1080 probe should be concentrated to the desired concentration for in vivo applications using the ultrafiltration concentration strategy.

**Mass spectrometry of Cn-1080 dyes**

The liquid chromatography-high resolution mass spectrometry (LC-HRMS) was operated under the specific conditions (ESI^+^ spray voltage, 4.5 kV, or ESI-spray voltage, -3.5 kV; nebulizer gas, 1.5 L/min; drying gas, 100 kPa; heat block temperature, 200^o^C; CDL temperature, 200^o^C; IT Area Vacuum, 1.0×10^-2^ Pa; TOF Area Vacuum, 5×10^-4^ Pa). The ion accumulation time was set to 10 ms, and the detector voltage was fixed at 1.6 kV. The mass number calibration (ion trap and TOF analyzer) was completed using a solution of trifluoroacetic acid (TFA) and sodium hydrate. Data acquisition and analysis were performed using the LCMS Solution version 3.0 software.

**Analysis of** **protein binding sites**

The pure protein or protein@dye samples were first subjected to SDS-PAGE gel electrophoresis, followed by slicing the corresponding gel bands and digesting with trypsin and chymotrypsin. Subsequently, the processed samples were analyzed by liquid chromatography-mass spectrometry (LC-MS/MS) and the raw files of the original results were obtained. Finally, the identification results of protein binding sites were obtained by analyzing the above-obtained raw files using Byonic software and searching the target protein database for labeling as a variable modification (C_44_H_44_N_2_O_4_, mass ≈ 690.3609 m/z).

**Experimental animals**

All animal experiments were conducted according to the protocols approved by the Animal Ethical Committee of The First Hospital of Jilin University (Procedure Number: 20210642). Balb/c and C57 mice (female, 6-8 weeks) were purchased from Liaoning Changsheng Biotechnology Co., Ltd. Bedding, nesting material, food, and water were provided ad libitum, and changed and replenished as required. The feeding environment was 20-22°C, 35-45% humidity, 12 h light-dark alternation.

**Mouse stroke model**

According to previous reports, the focal cerebral ischemia was induced by photothrombotic stroke mode.^[2–4]^ The operation is as follows: C57 mice were selected as the study object and were first intravenously injected with Bengal rose (0.4 mg diluted in 0.2 mL PBS) followed by 532 nm laser (diameter 2 mm, power density 1.59 W/cm^2^) irradiating the intact skull for 10 min. The laser was vertically centered 1 mm posterior and 2 mm lateral from bregma. Meanwhile, the mild, moderate, and severe degrees of stroke injury were simulated by adjusting the laser irradiation time, corresponding to 1 min, 5 min, and 10 min. Notably, C57 mice were anesthetized with isoflurane (4% induction; 2% maintenance) and body temperature was maintained at 37 ± 0.5°C throughout the modeling process. In addition, the sham group underwent an operation without the laser induction.

**TTC staining**

For the mouse stroke model, the whole brain was immersed in a 2% 2,3,5-Triphenyl tetrazolium chloride staining (TTC) solution for 20 min at 37°C.

**Evans Blue staining**

Evans Blue (EB, 2% in normal saline, 4 mL/kg) was injected via the tail vein into the mouse 10 min after the stroke and 3 h before sacrifice. The whole brain was then removed to reveal blood-brain barrier disruption in vitro.

**NIR-II** **fluorescence** **imaging**

NIR-II imaging set-up was built in-house and consisted of a camera (Princeton Instruments, NIRvana-640), laser (Artemis Intelligent Imaging), and off-the-shelf optics (Thorlabs, Edmund optics, etc.). The excitation light was generated by an 808 nm, 980 nm, or 1064 nm fiber-coupled diode laser with adjustable power density. Different long-pass (LP) filters were combined to collect different waveband images in the NIR-II window. All mice were shaved with depilatory cream and anesthetized with chloral hydrate or isoflurane before the experiment. During imaging experiments, at least 3 mice were used as parallel controls. All fluorescence images were processed and analyzed by Image J software.

**Statistical Analyses**

Data points were collected and compiled in Microsoft Excel, and statistical analyses were performed using GraphPad Prism and Origin Pro software. Statistical significance was determined by a two-tailed Student’s t-test, with a p-value of less than 0.05 considered significant. For the continuous variables, the data were presented as mean ± standard deviation.


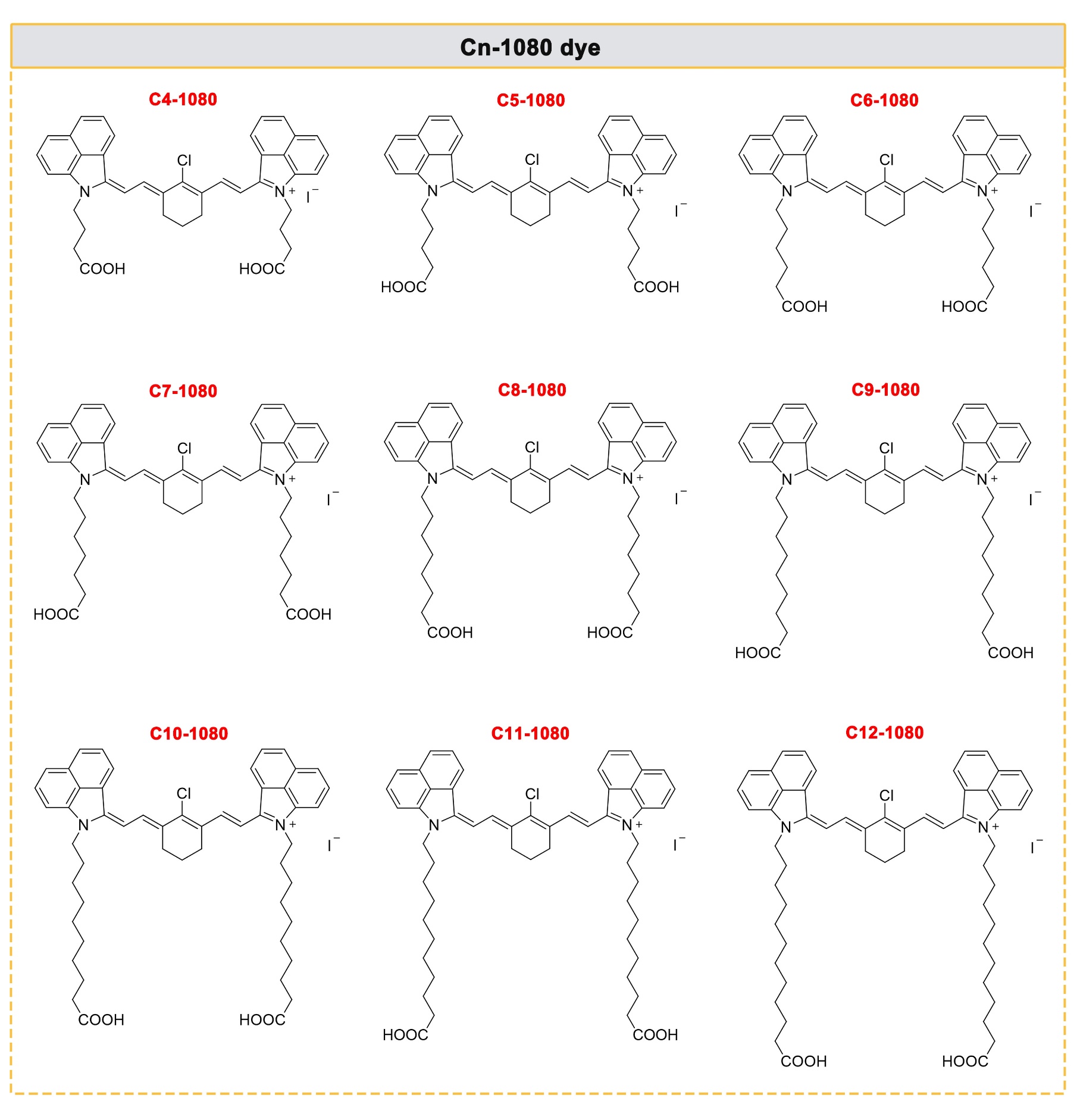


**Figure S2.** Molecular structural formula of Cn-1080 dyes with different side chain lengths.


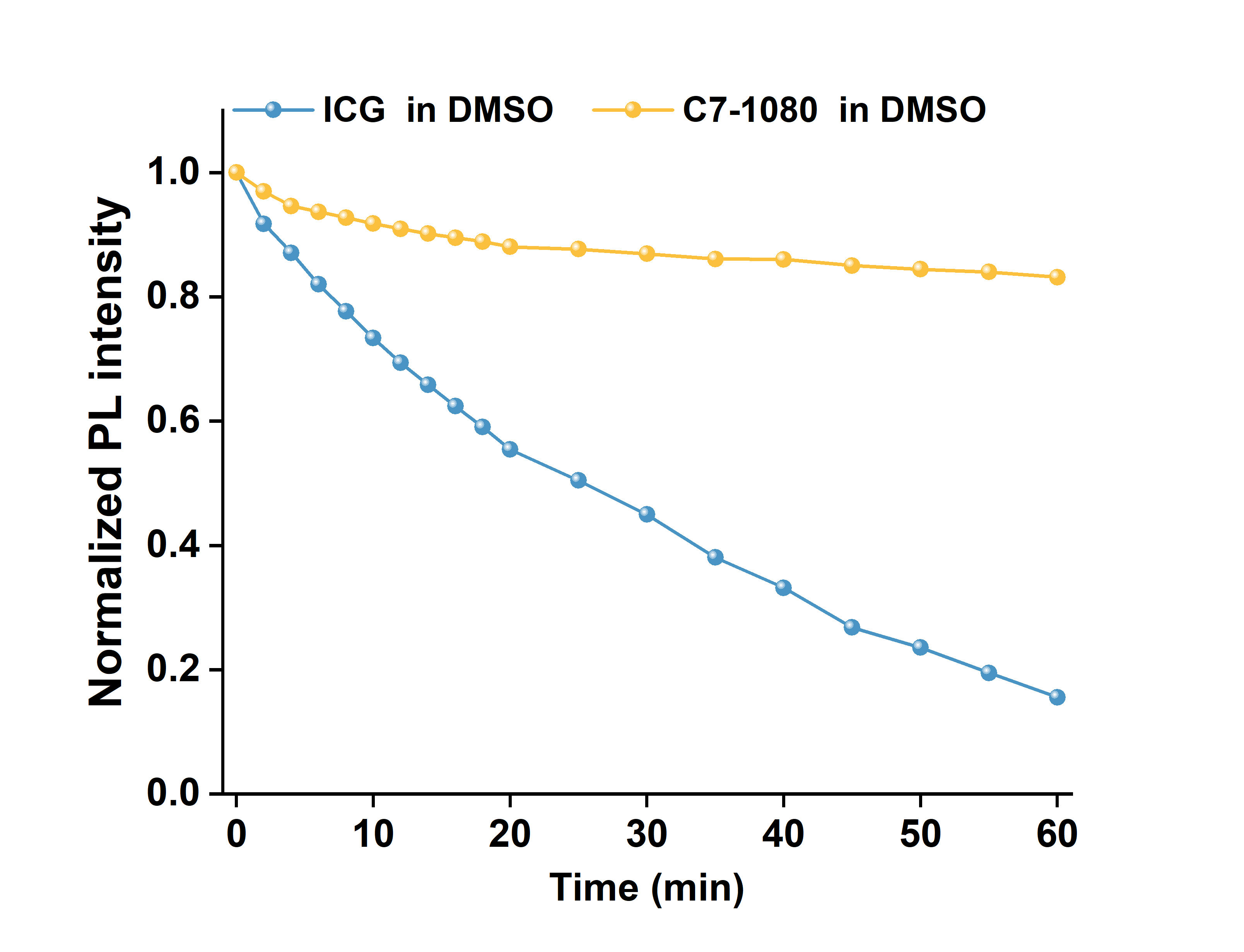


**Figure S3**. Photostability of ICG and C7-1080 in DMSO

**
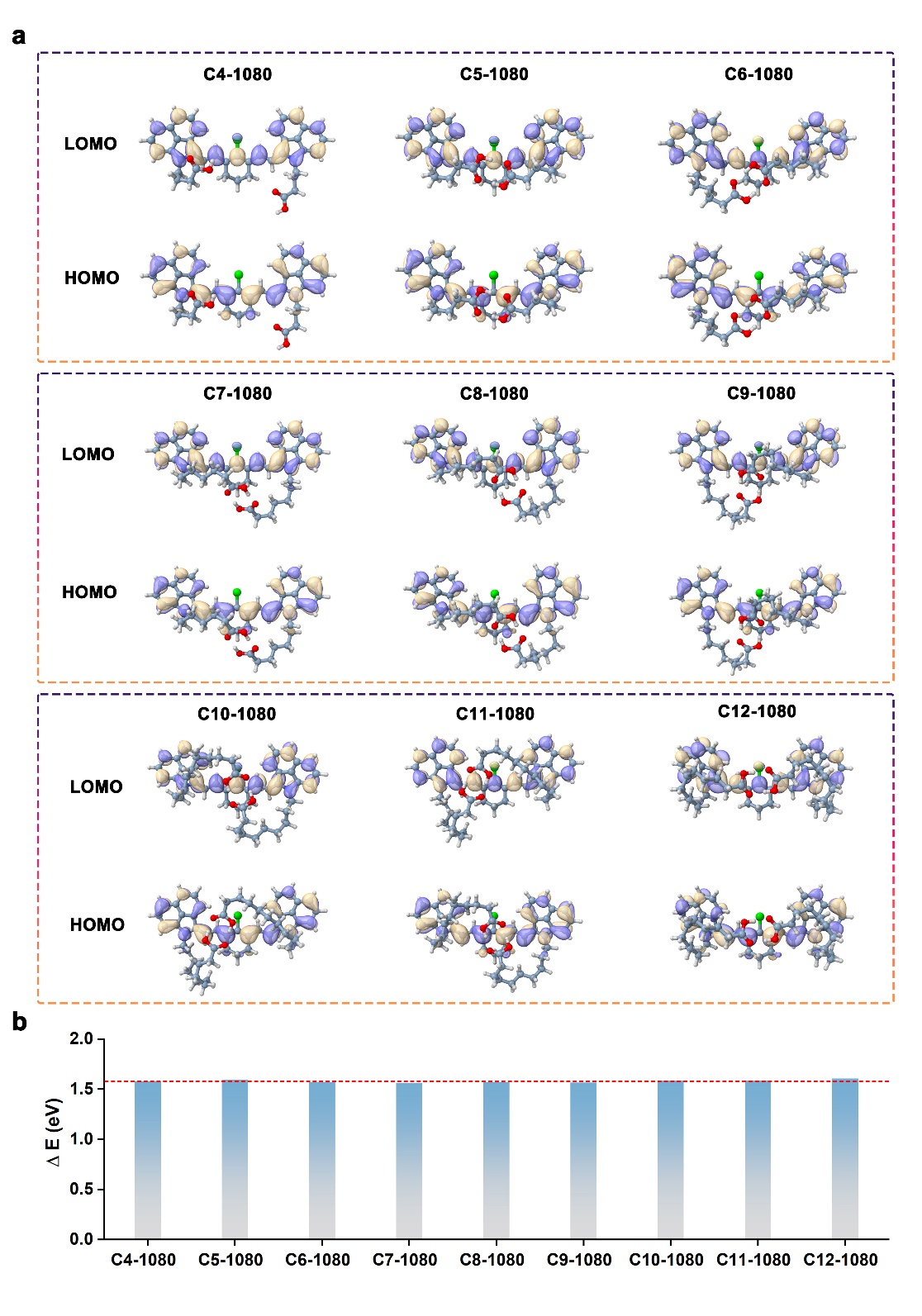
**

**Figure S4.** a) Schematic of the HOMO and LUMO energy levels and b) the corresponding bandgap for different Cn-1080 dyes. The HOMO and LUMO energy levels were plotted based on the optimized S0 and S1 geometries using Gaussian (b31yp/6-31g(d)).

**
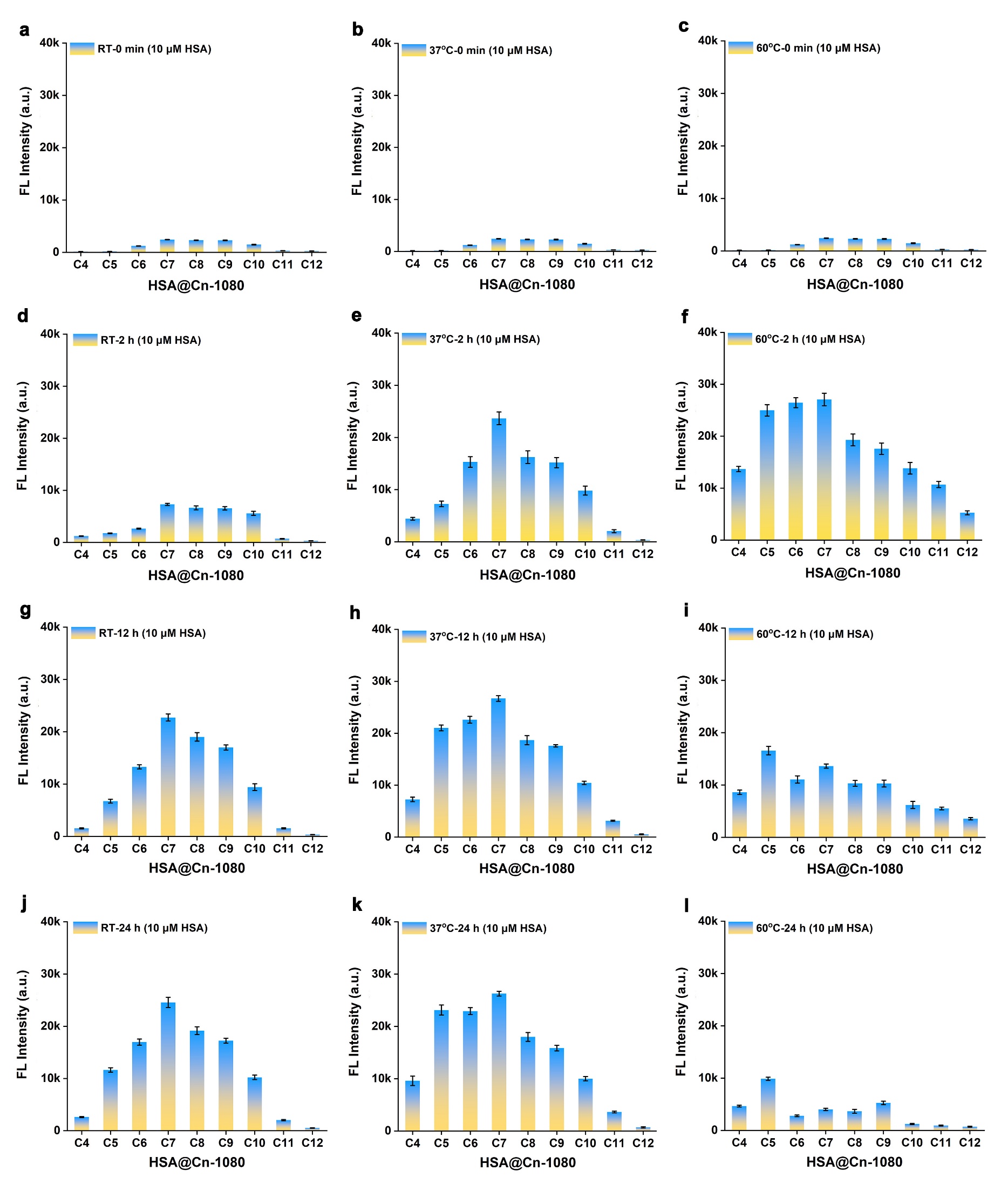
**

**Figure S5.** Fluorescence intensity of HSA and Cn-1080 dyes mixed under different reaction conditions, including a) RT-0 min, b) 37^o^C-0 min, c) 60^o^C-0 min, d) RT-2 h, e) 37^o^C-2 h, f) 60^o^C-2 h, g) RT-12 h, h) 37^o^C-12 h, i) 60^o^C-12 h, j) RT-24 h, k) 37^o^C-24 h, and l) 60^o^C-24 h.

**
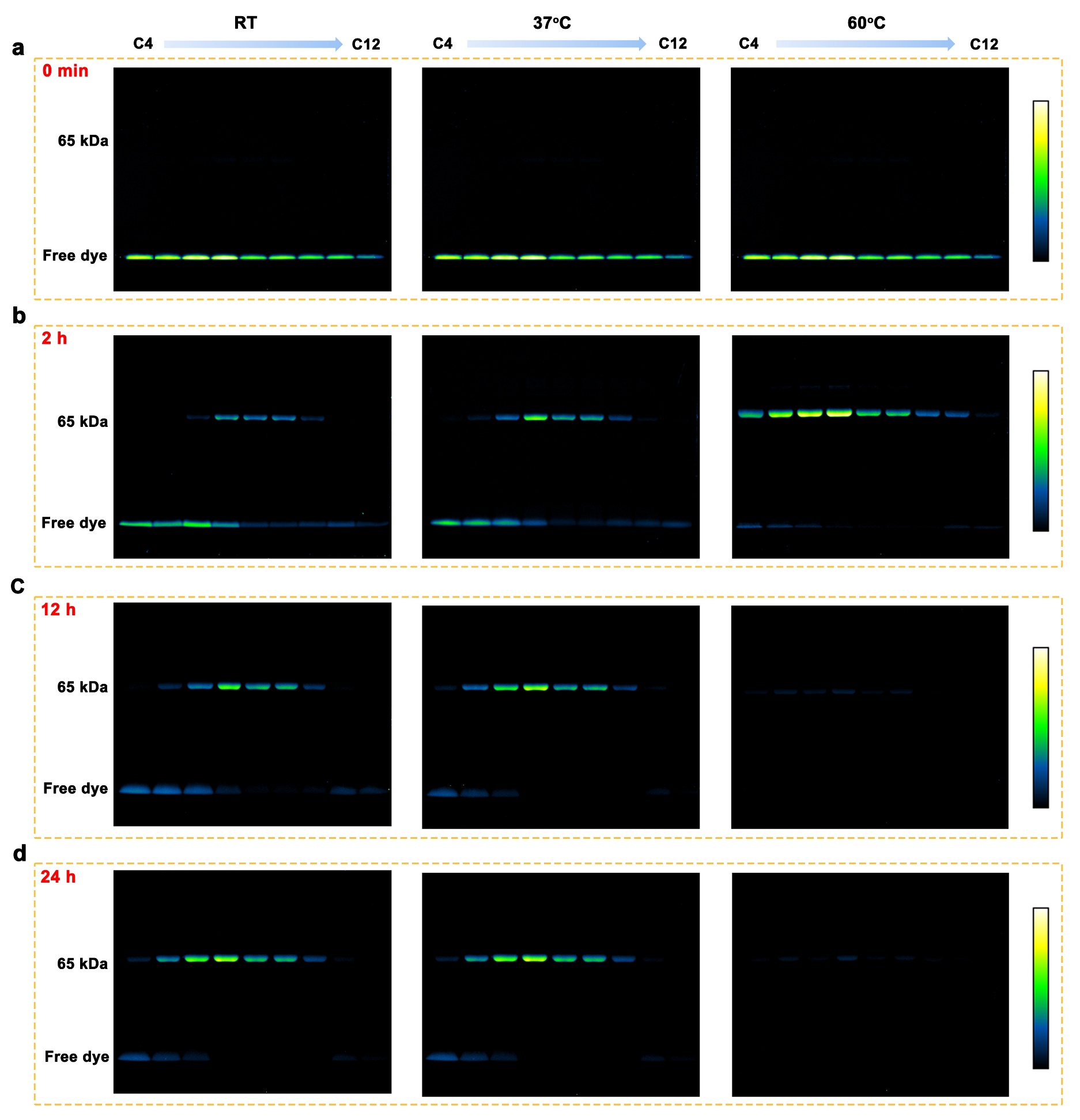
**

**Figure S6.** Gel electrophoresis analysis of HSA and Cn-1080 dyes after reacting at RT, 37^o^C, and 60^o^C for different times, including a) 0 min, b) 2 h, c) 12 h, d) 24 h.

**
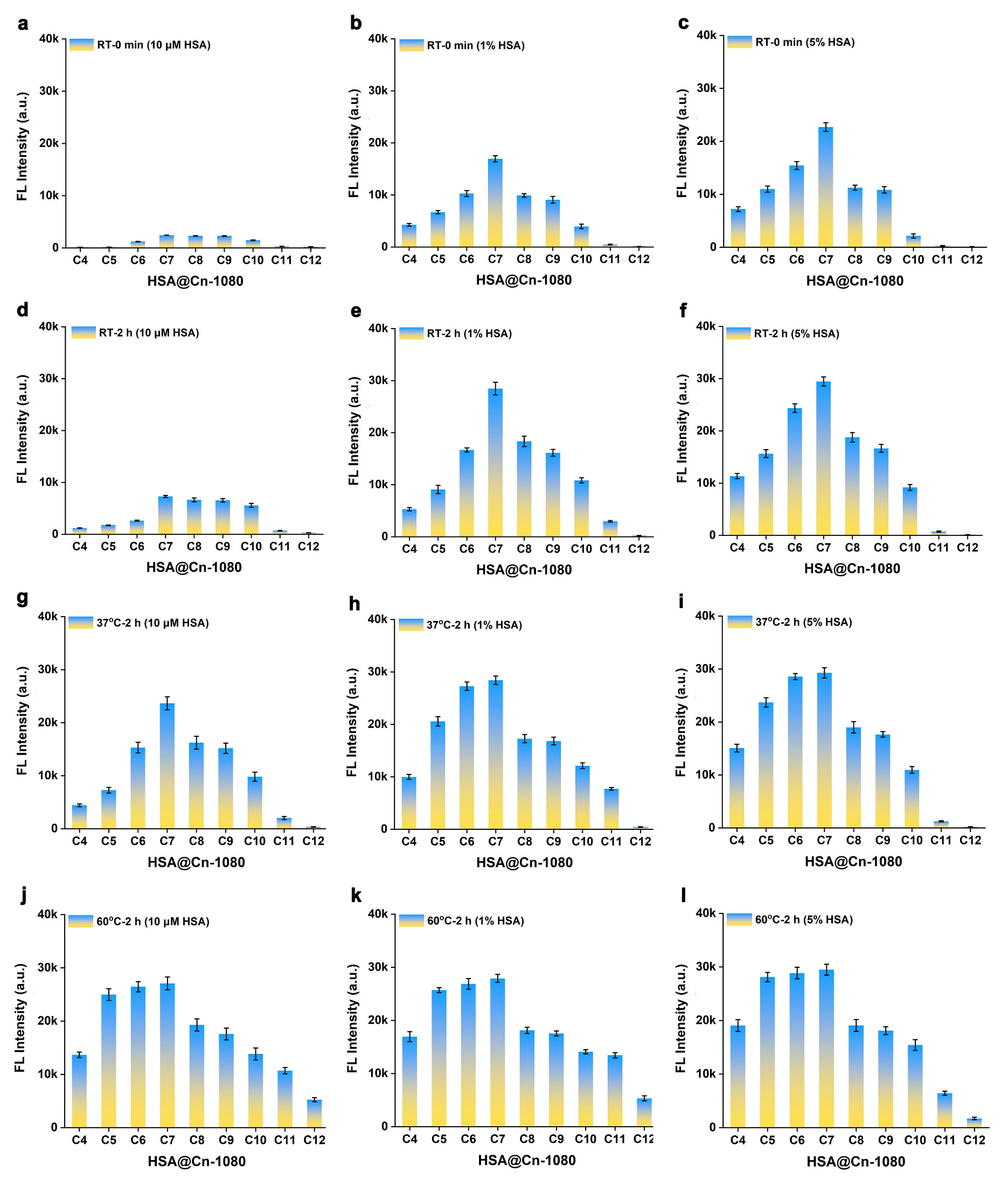
**

**Figure S7.** Fluorescence intensity of Cn-1080 dye after reaction with 10 μM, 1%, 5% concentration of HSA at different temperatures, including a) RT-0 min (10 μM HSA), b) RT-0 min (1% HSA), c) RT-0 min (5% HSA), d) RT-2 h (10 μM HSA), e) RT-2 h (1% HSA), f) RT-2 h (5% HSA), g) 37^o^C -2 h (10 μM HSA), h) 37^o^C-2 h (1% HSA), i) 37^o^C-2 h (5% HSA), j) 60^o^C -2 h (10 μM HSA), k) 60-2 h (1% HSA), and l) 60^o^C-2 h (5% HSA).


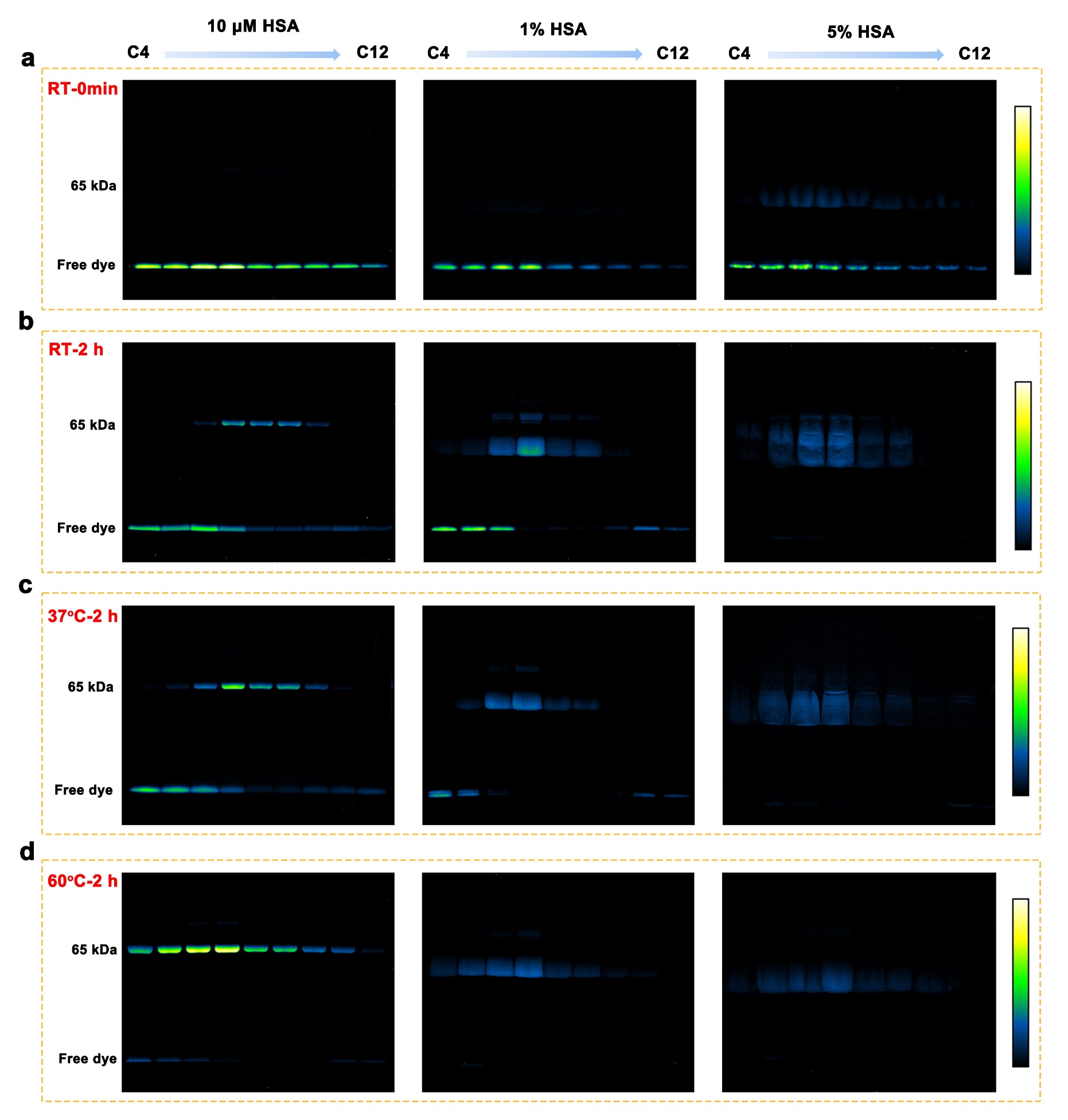


**Figure S8.** Gel electrophoresis analysis of Cn-1080 dye after reaction with 10 μM, 1%, and 5% concentration of HSA at different temperatures, including a) RT-0 min, b) RT-2 h, c) 37^o^C-2 h, and d) 60^o^C-2 h.

**
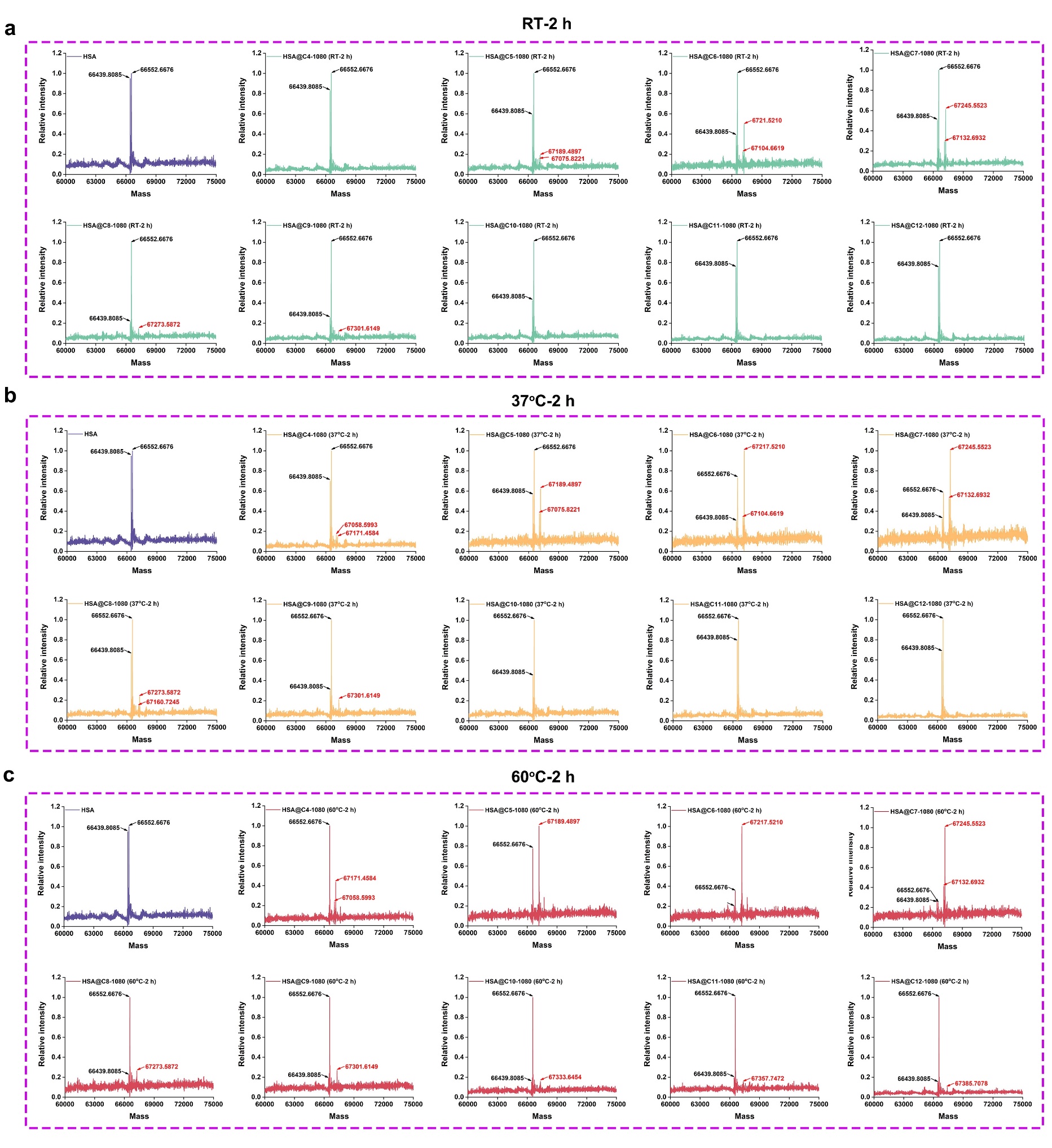
**

**Figure S9.** High-resolution mass spectrometry of HSA and Cn-1080 dyes after reacting at different temperatures for 2 h, including a) RT, b) 37^o^C, and c) 60^o^C.

**
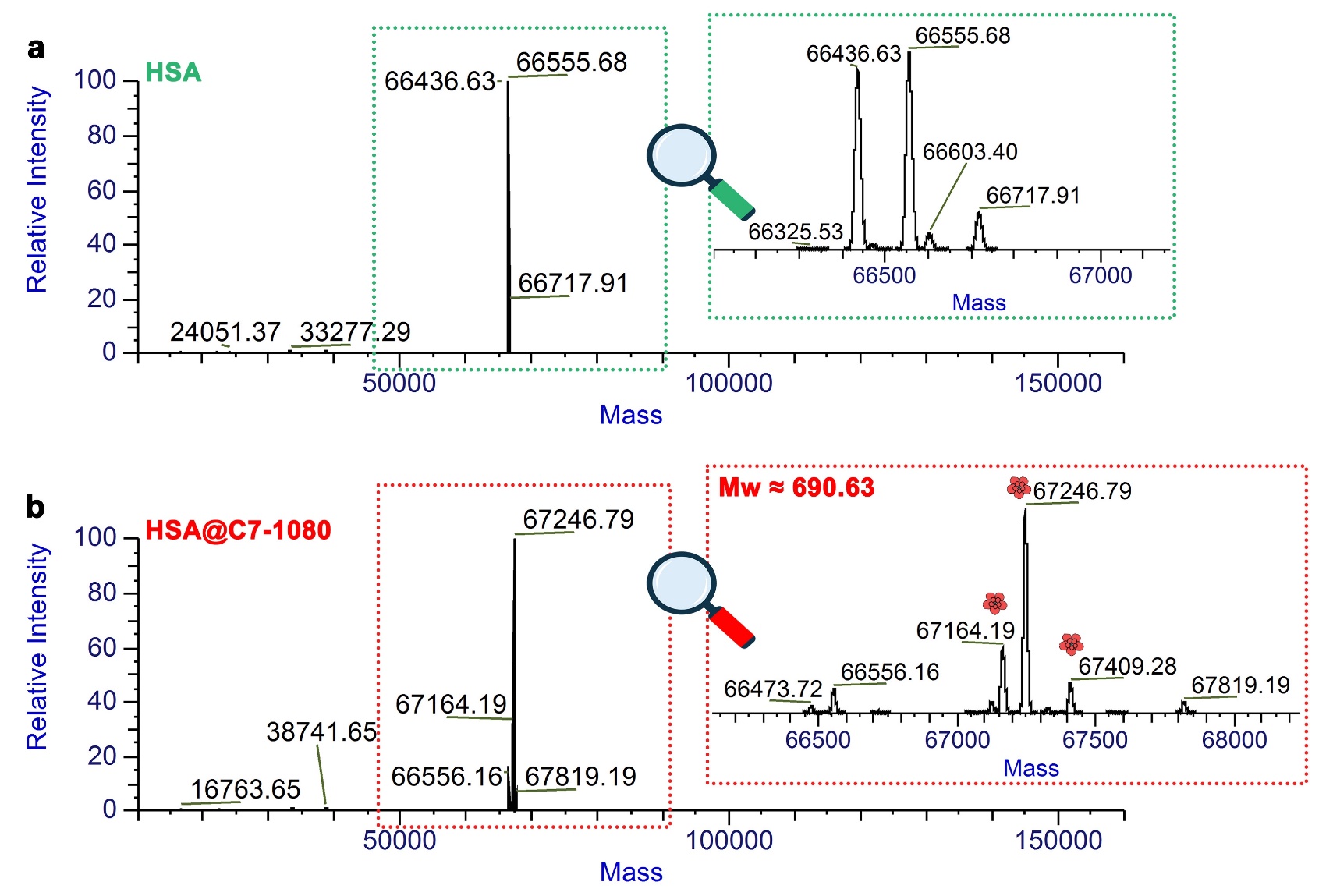
**

**Figure S10.** High-resolution mass spectrometry of a) HSA and b) HSA@C7-1080.


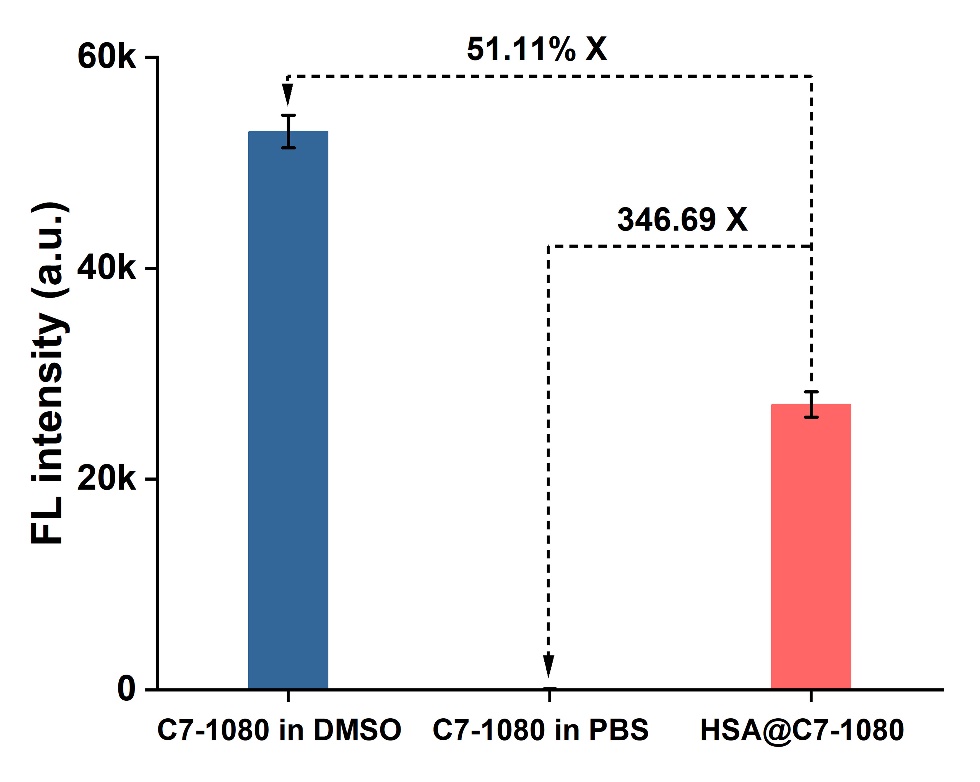


**Figure S11.** NIR-II brightness of C7-1080 in DMSO, C7-1080 in PBS, and HSA@C7-1080.


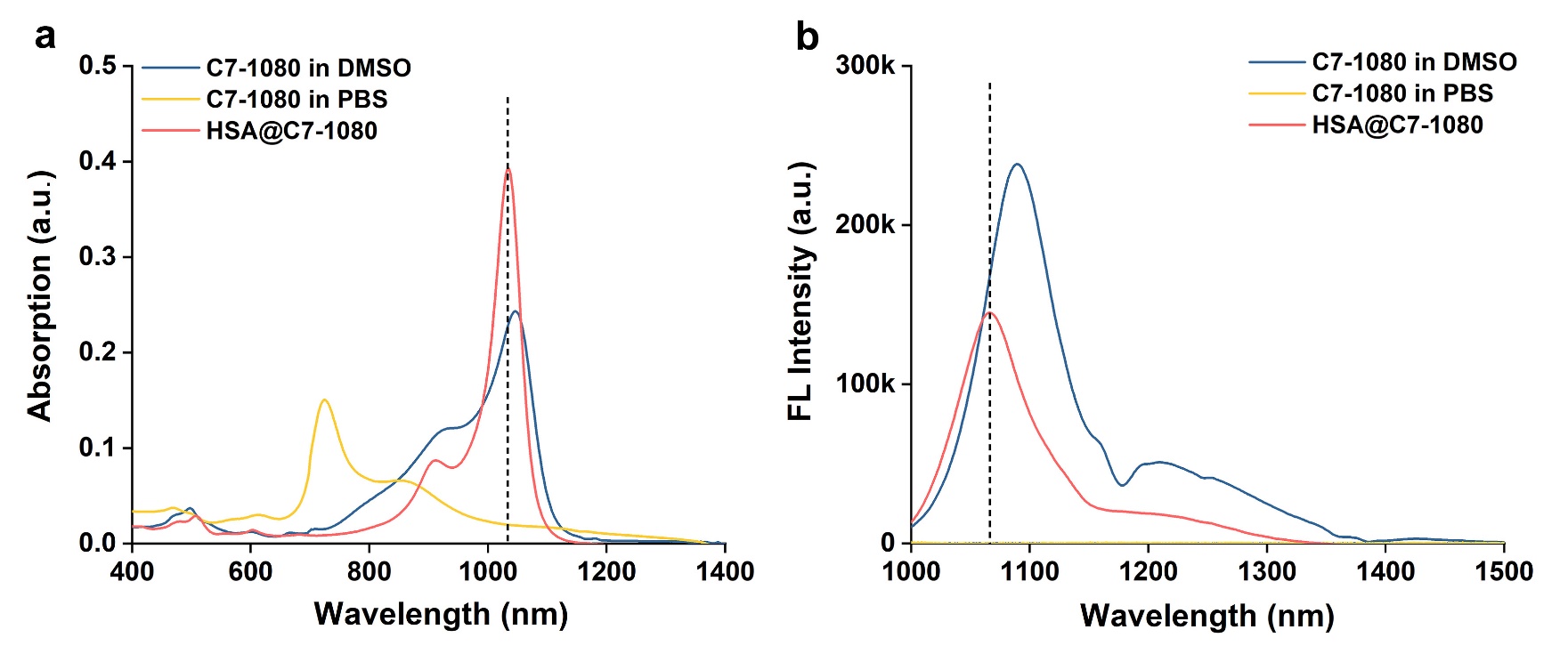


**Figure S12.** a) UV absorption spectra and b) fluorescence spectra of C7-1080 in DMSO, C7-1080 in PBS, and HSA@C7-1080.


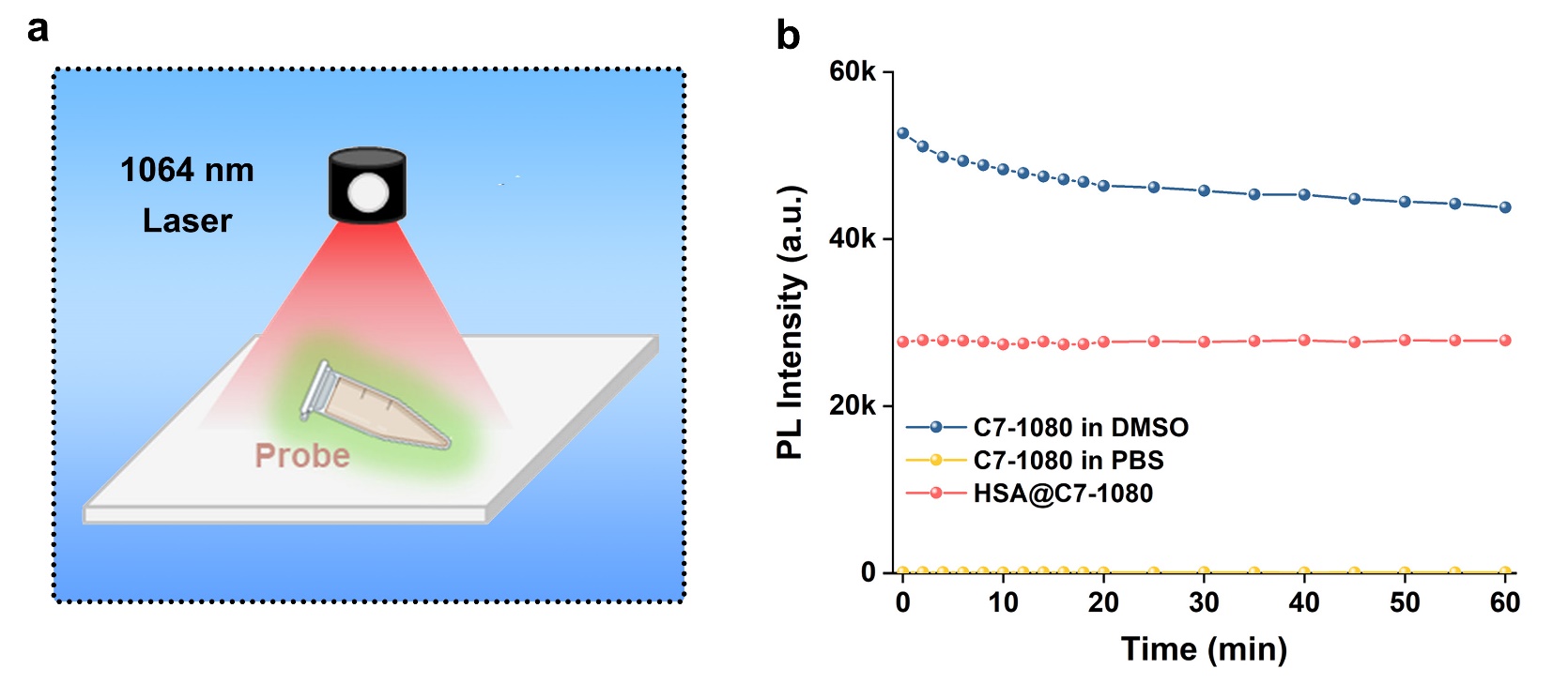


**Figure S13.** a) Schematic of photostability test for different probes. b) Photostability of C7-1080 in DMSO, C7-1080 in PBS, and HSA@C7-1080 probes under 1064 nm excitation.

**
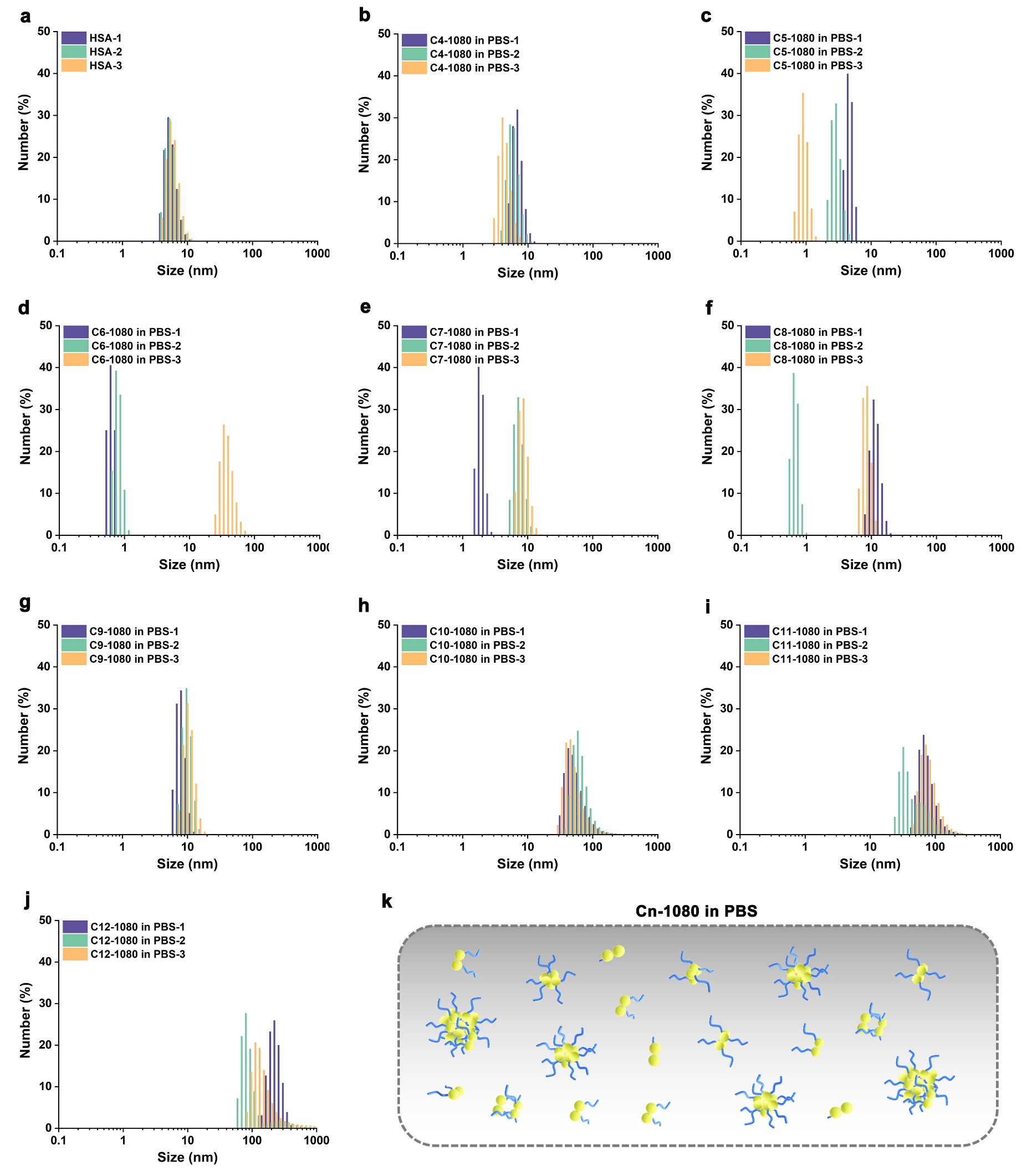
**

**Figure S14.** DLS analysis of a) HSA, b) C4-1080 in PBS, c) C5-1080 in PBS, d) C7-1080 in PBS, e) C8-1080 in PBS, f) C9-1080 in PBS, h) C10-1080 in PBS, i) C11-1080 in PBS, j) C12-1080 in PBS. k) Schematic of the morphology of Cn-1080 dyes in PBS solution.


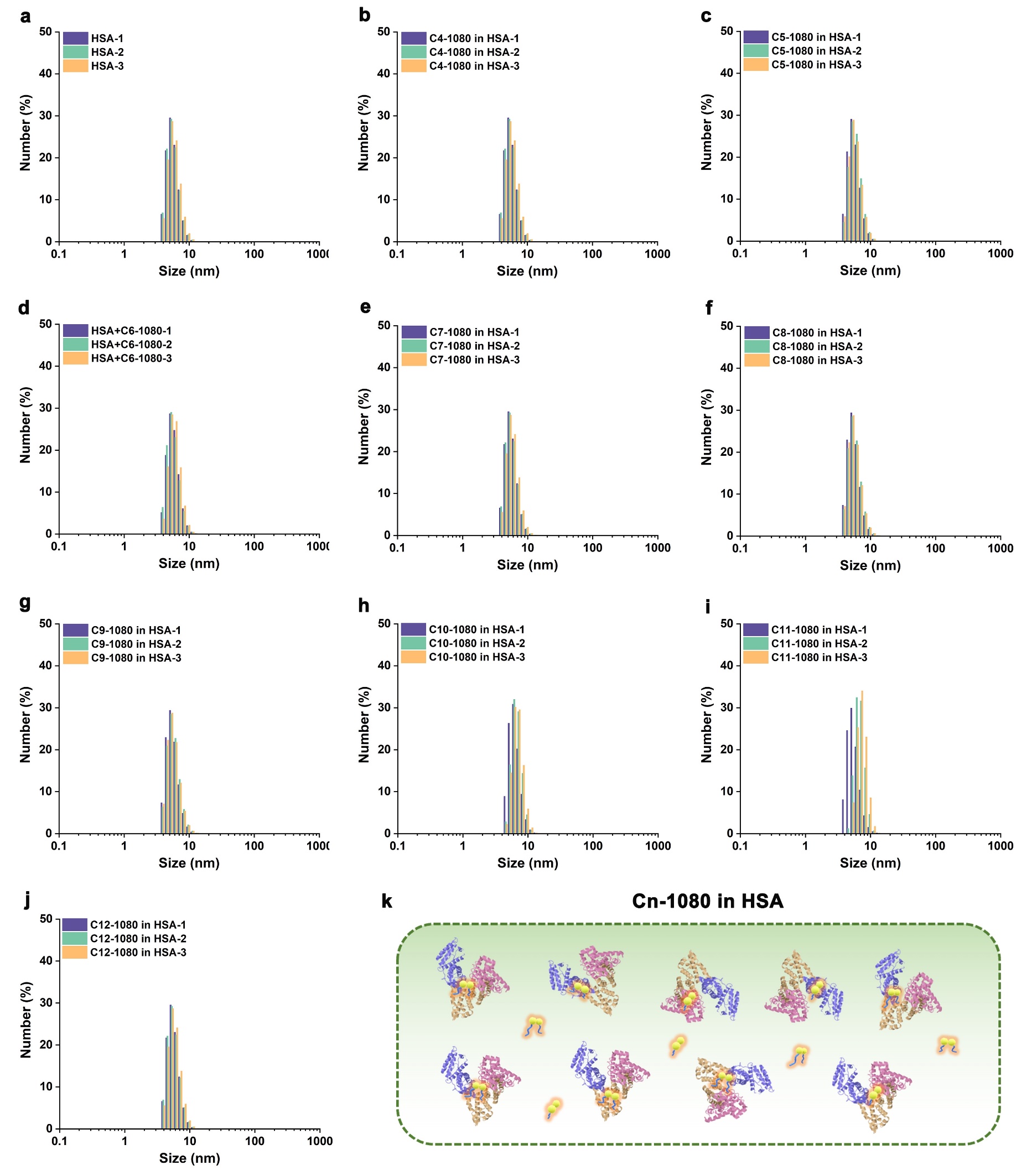


**Figure S15.** DLS analysis of a) HSA, b) C4-1080 in HSA, c) C5-1080 in HSA, d) C7-1080 in HSA, e) C8-1080 in HSA, f) C9-1080 in HSA, h) C10-1080 in HSA, i) C11-1080 in HSA, j) C12-1080 in HSA. k) Schematic of the morphology of Cn-1080 dyes in HSA solution.

**
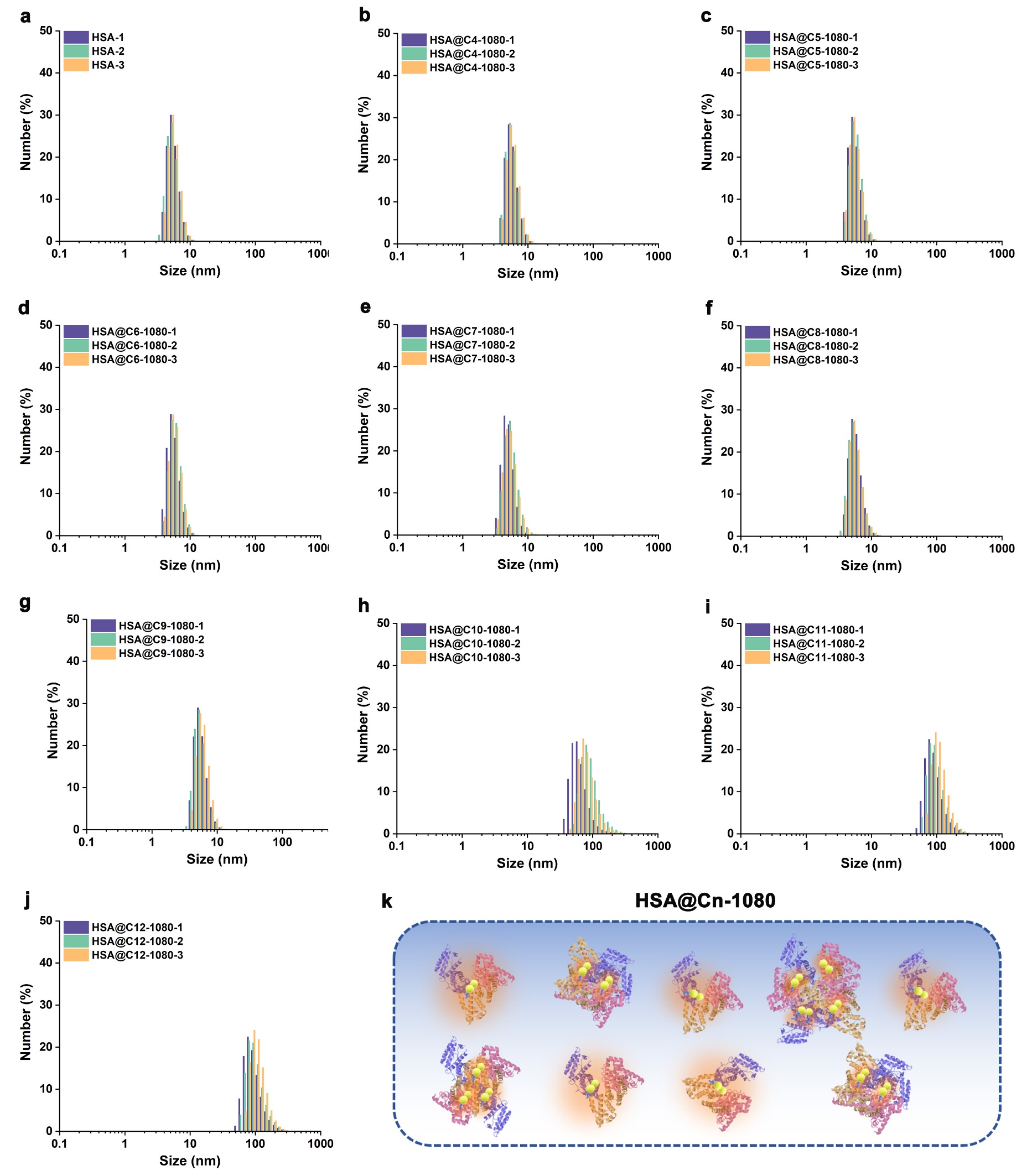
**

**Figure S16.** DLS analysis of a) HSA, b) HSA@C4-1080, c) HSA@C5-1080, d) HSA@C6-1080, e) HSA@C7-1080, f) HSA@C8-1080, g) HSA@C9-1080, h) HSA@C10-1080, i) HSA@C11-1080, j) HSA@C12-1080. k) Schematic of the morphology of HSA@Cn-1080 dyes.

**
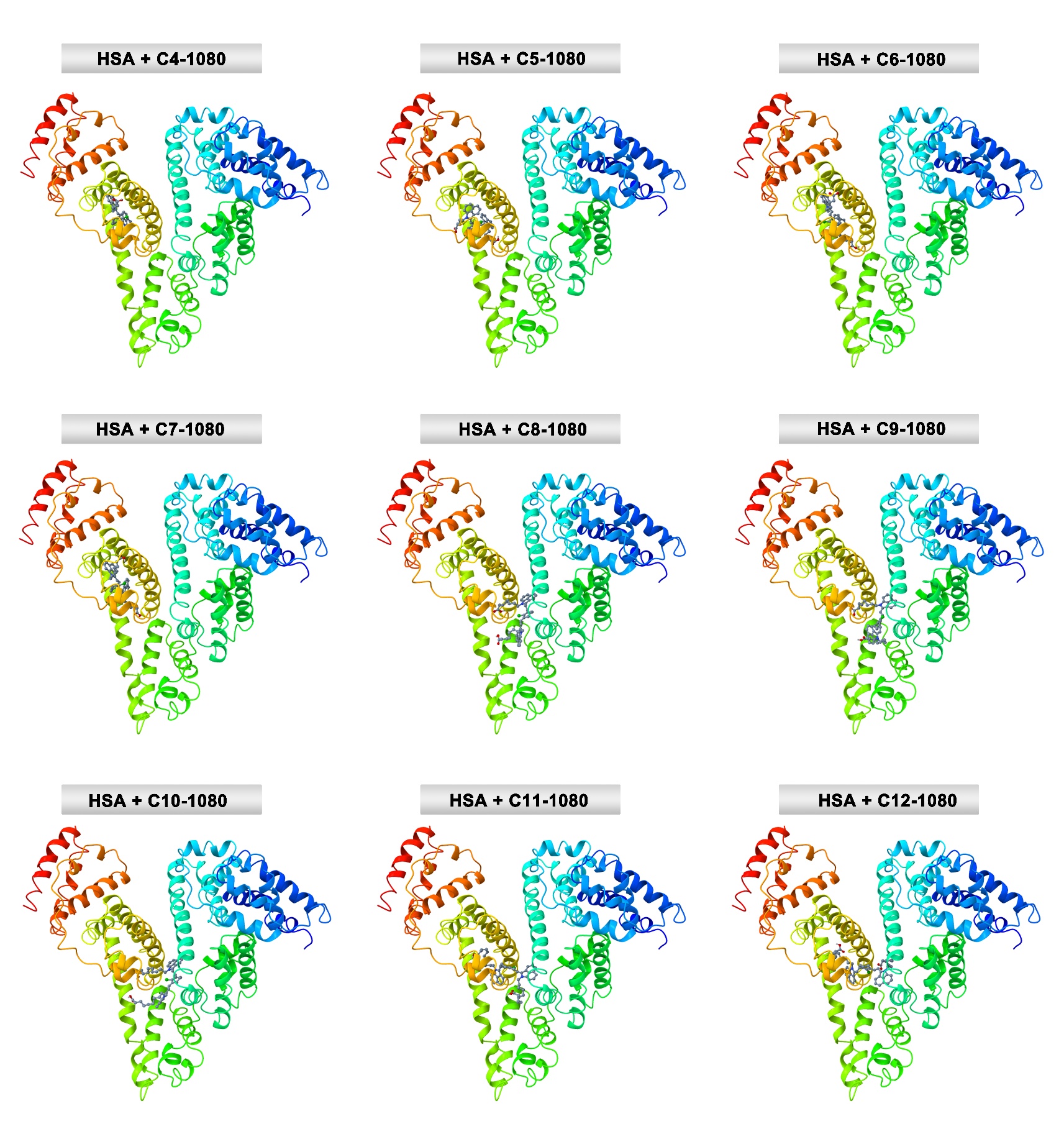
**

**Figure S17.** Theoretical simulation of different Cn-1080 dyes binding to HSA proteins by gliding docking mode.

**
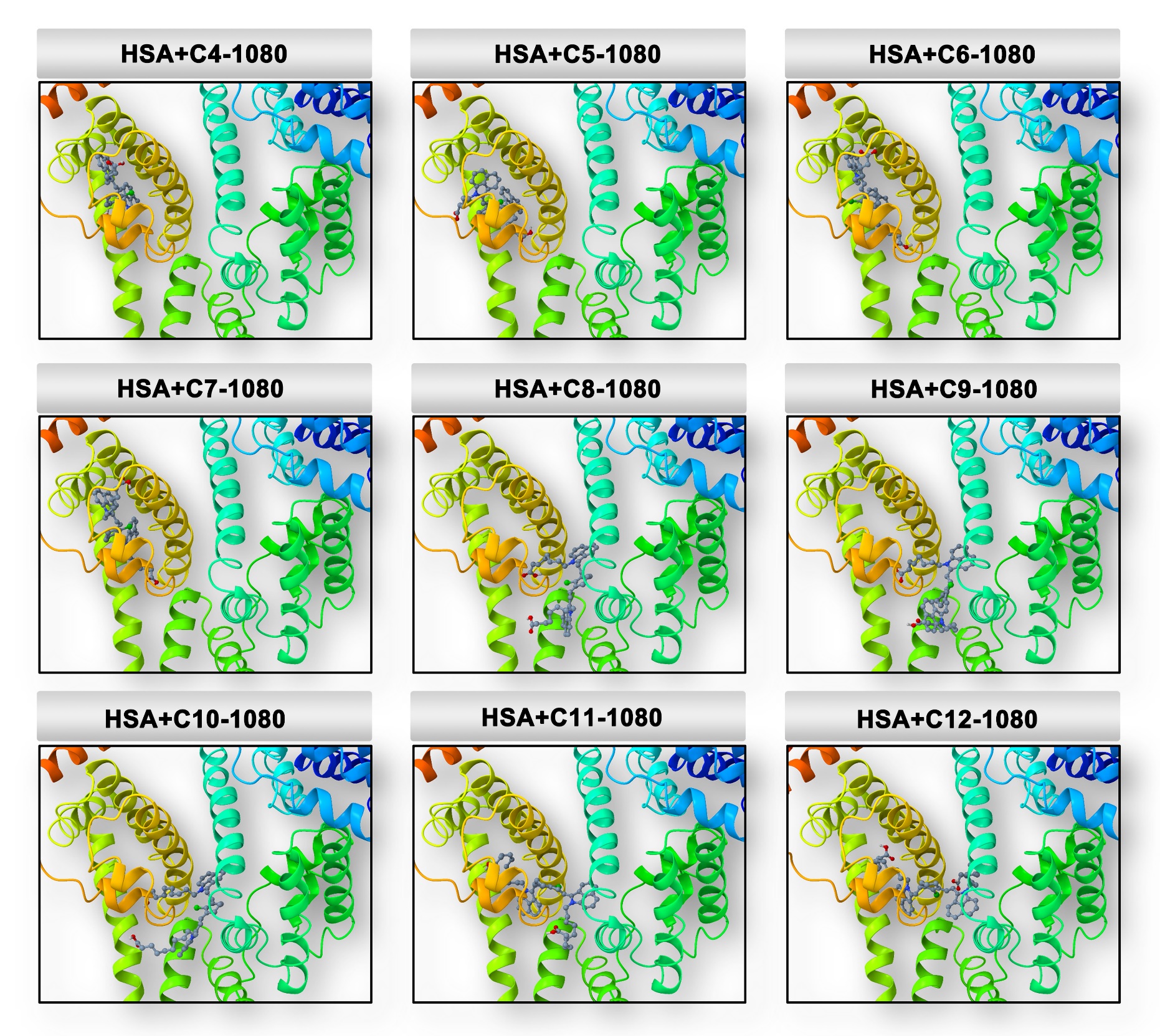
**

**Figure S18.** A detailed magnification of the docking simulation of different Cn-1080 dyes with HSA protein.

**
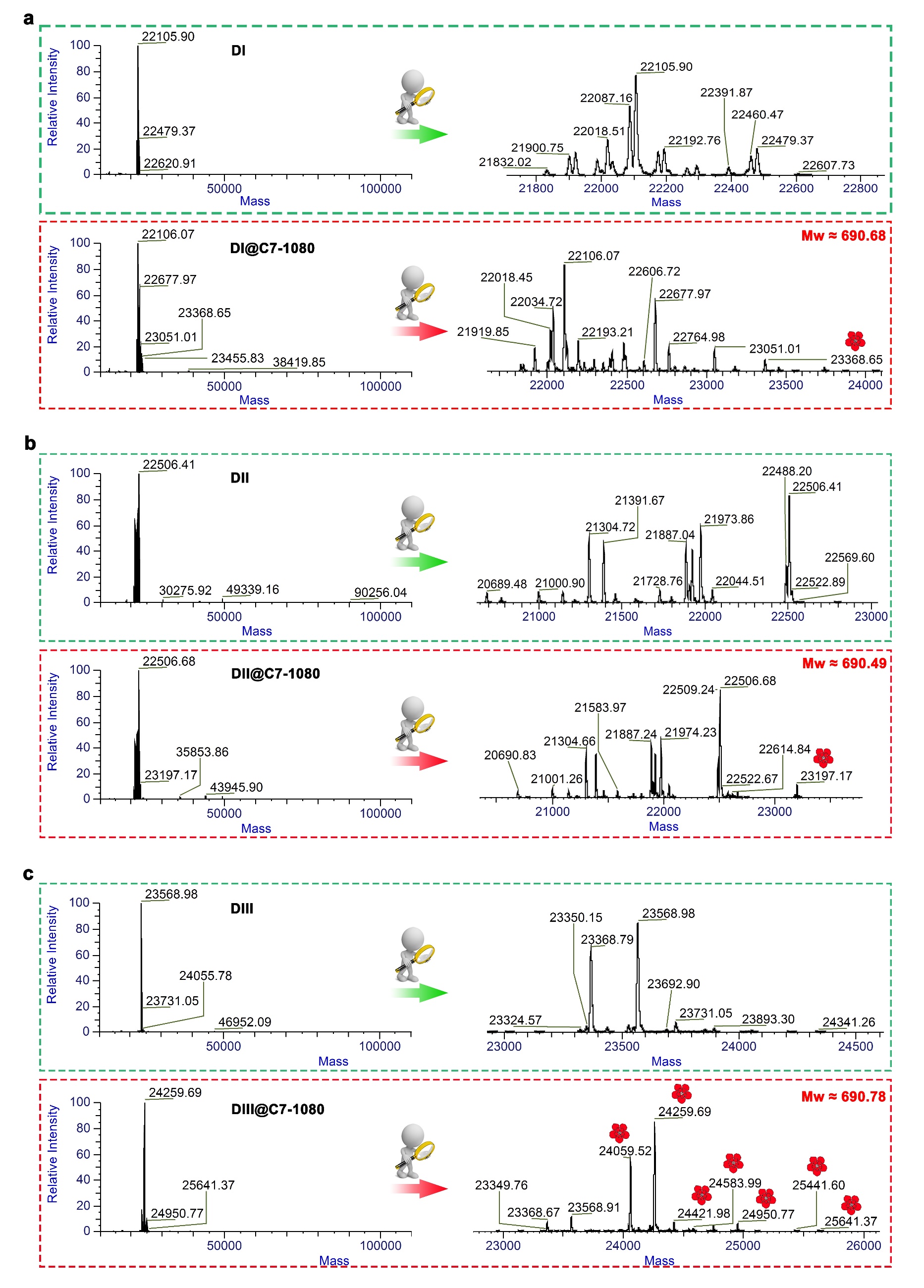
**

**Figure S19.** a) High-resolution mass spectrometry of DI and DI@C7-1080. b) High-resolution mass spectrometry of DII and DII@C7-1080. c) High-resolution mass spectrometry of DIII and DIII@C7-1080.

**
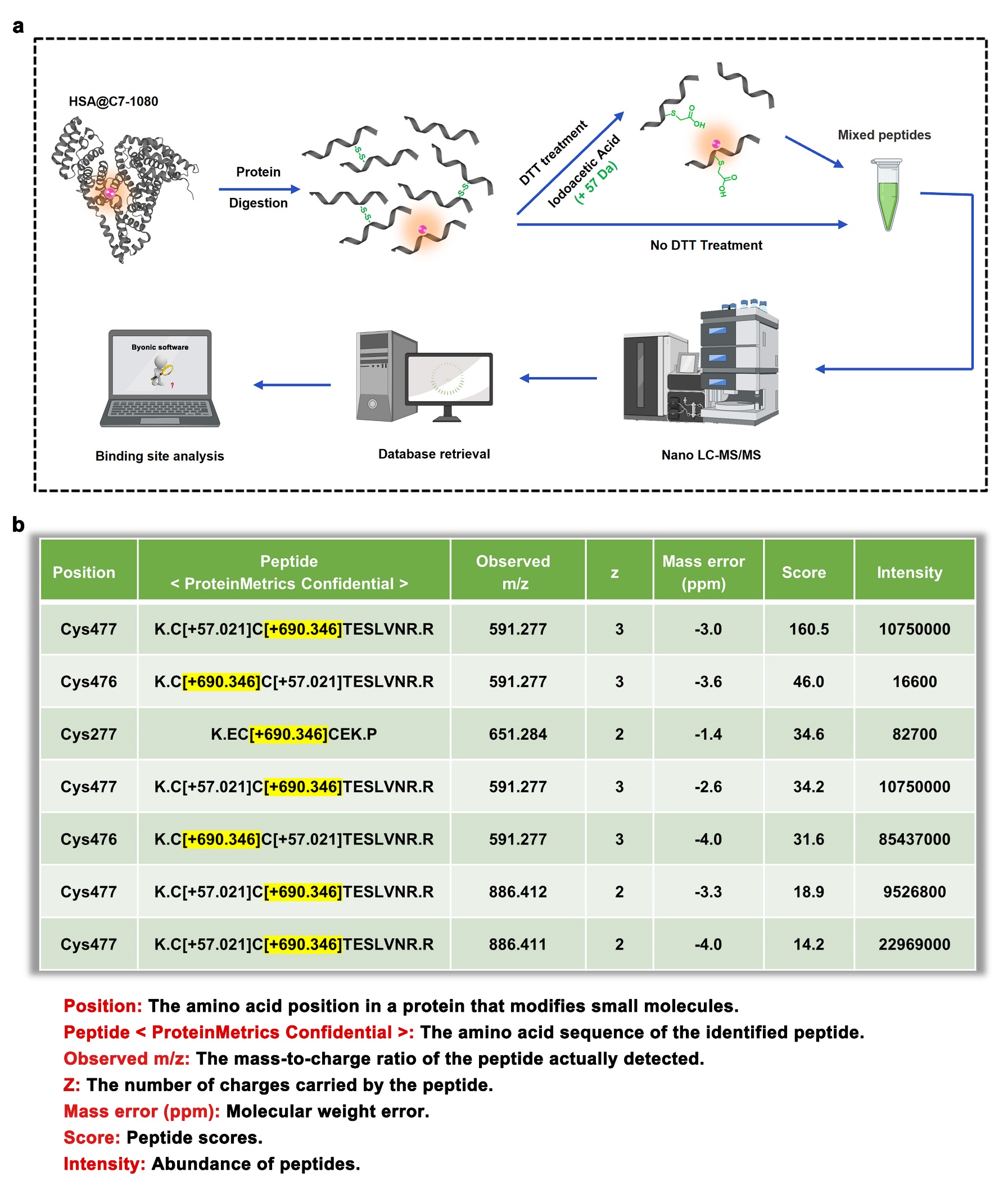
**

**Figure S20.** a) Proteomics analysis flowchart of HSA@C7-1080. b) Peptide information and specific binding sites containing the C7-1080 dyes obtained by proteomic analysis.

**
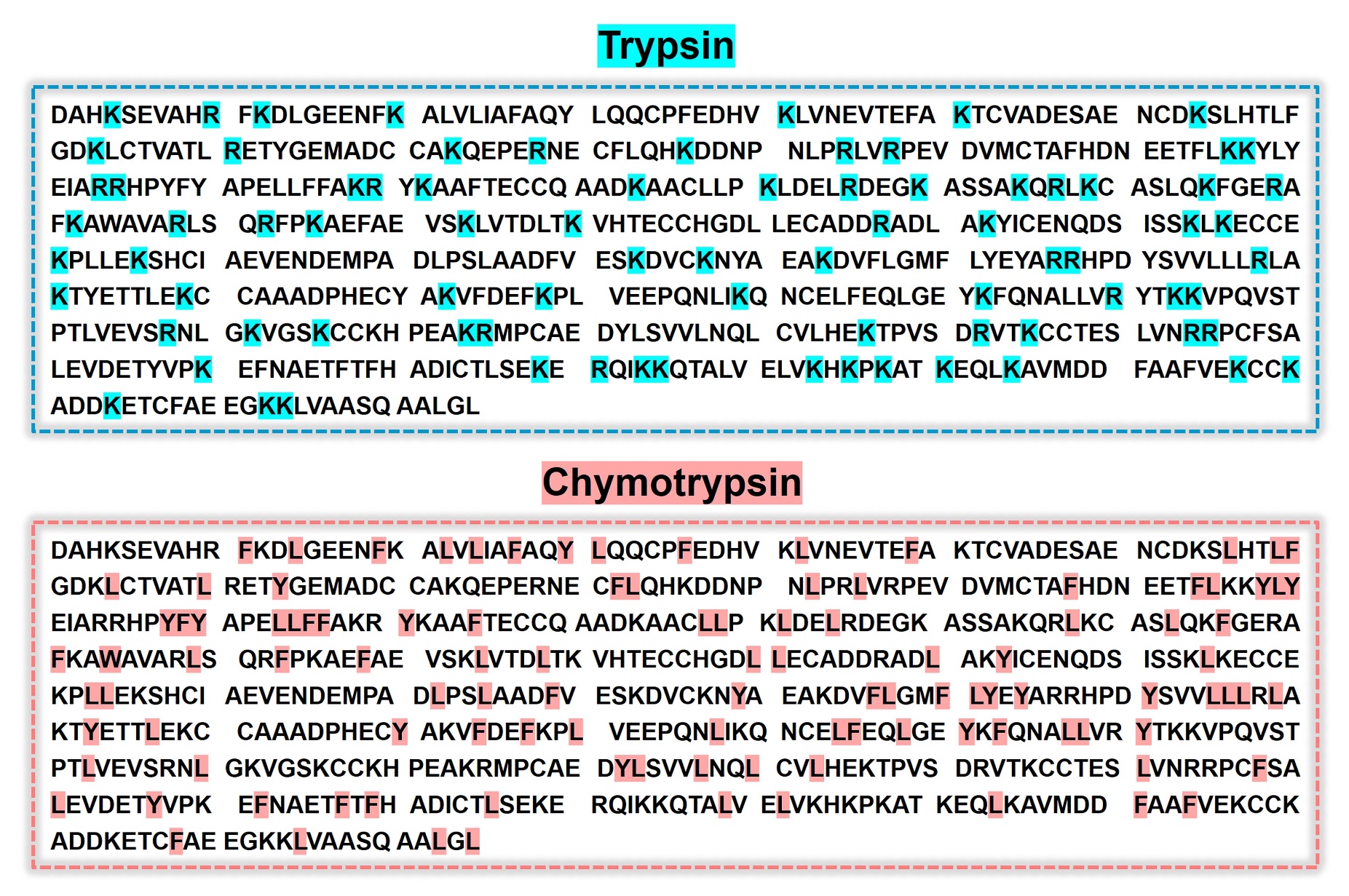
**

**Figure. S21.** Digestion enzymes and their targeting cleavage sites on HSA.

**
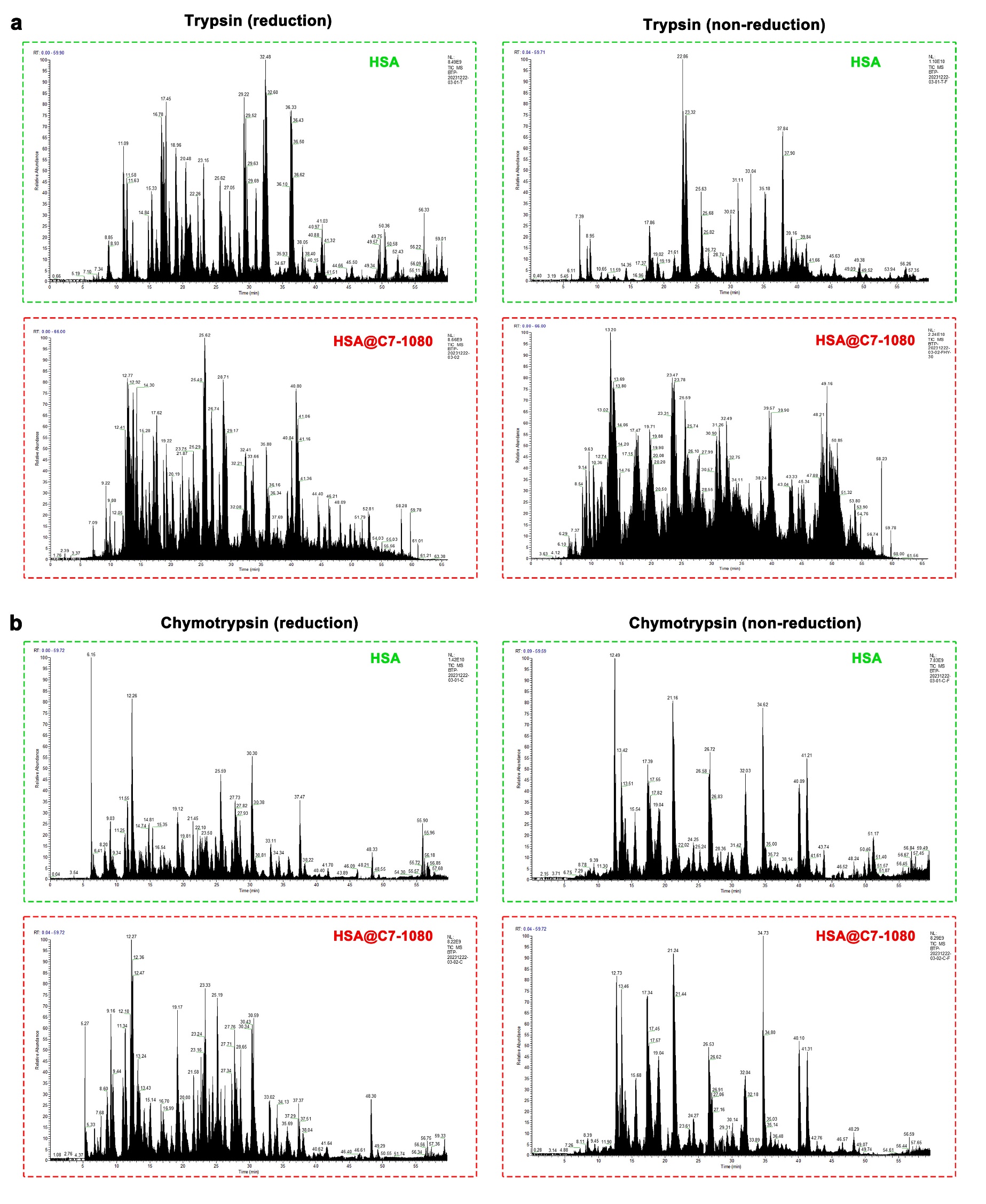
**

**Figure S22.** a) Total ion flow chromatogram after trypsin enzyme digestion of HSA and HSA@C7-1080. b) Total ion flow chromatogram after chymotrypsin enzyme digestion of HSA and HSA@C7-1080.

**
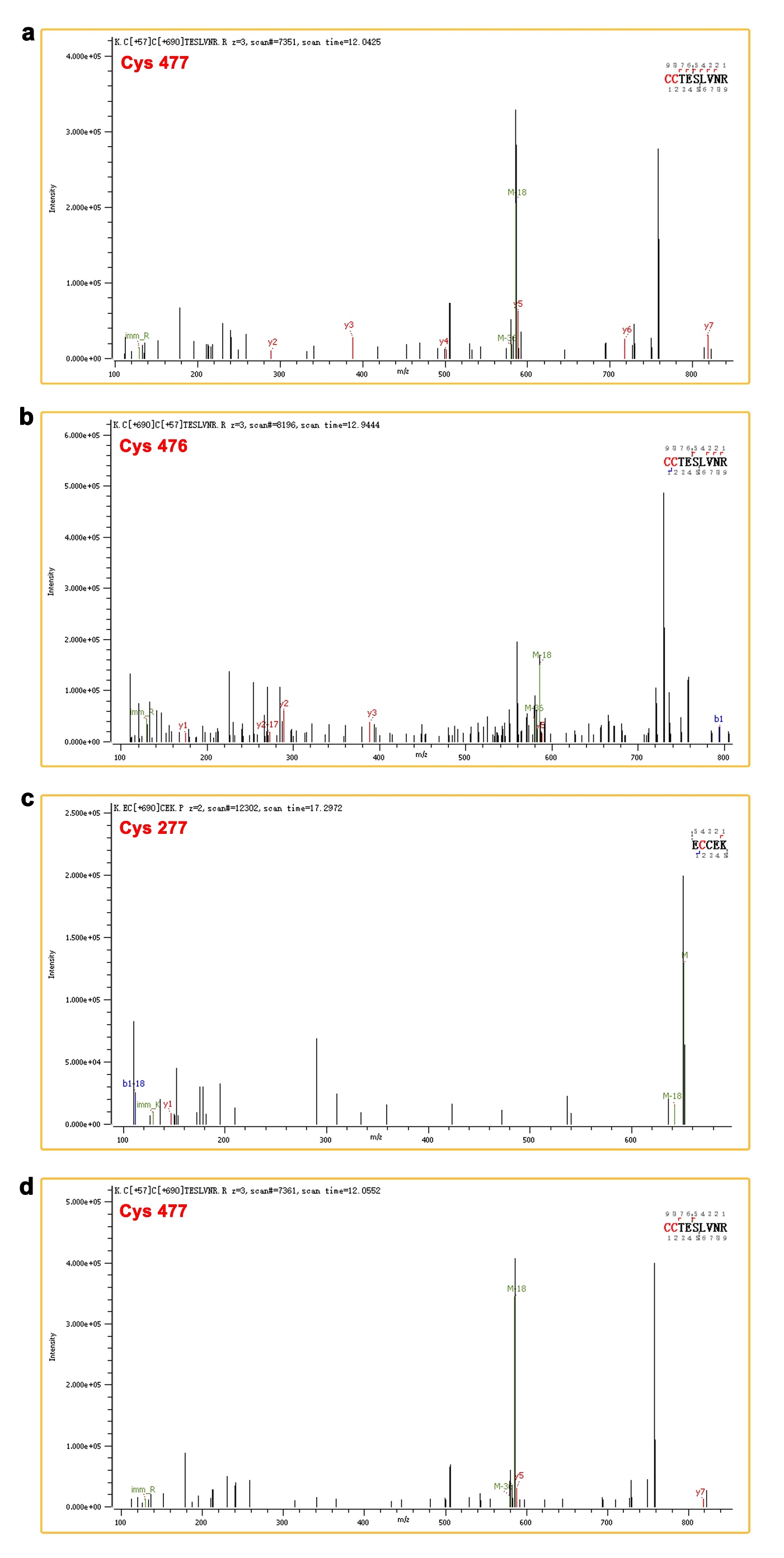
**

**Figure S23.** a) The secondary mass spectra of the sequence of CCTESLVNR (Cys477). b) The secondary mass spectra of the sequence of CCTESLVNR (Cys476). c) The secondary mass spectra of the sequence of ECCEK (Cys277). d) The secondary mass spectra of the sequence of CCTESLVNR (Cys477). The measurement accuracy for the corresponding fragments was < 0.02 Da (y ion series; y1–y8).

**
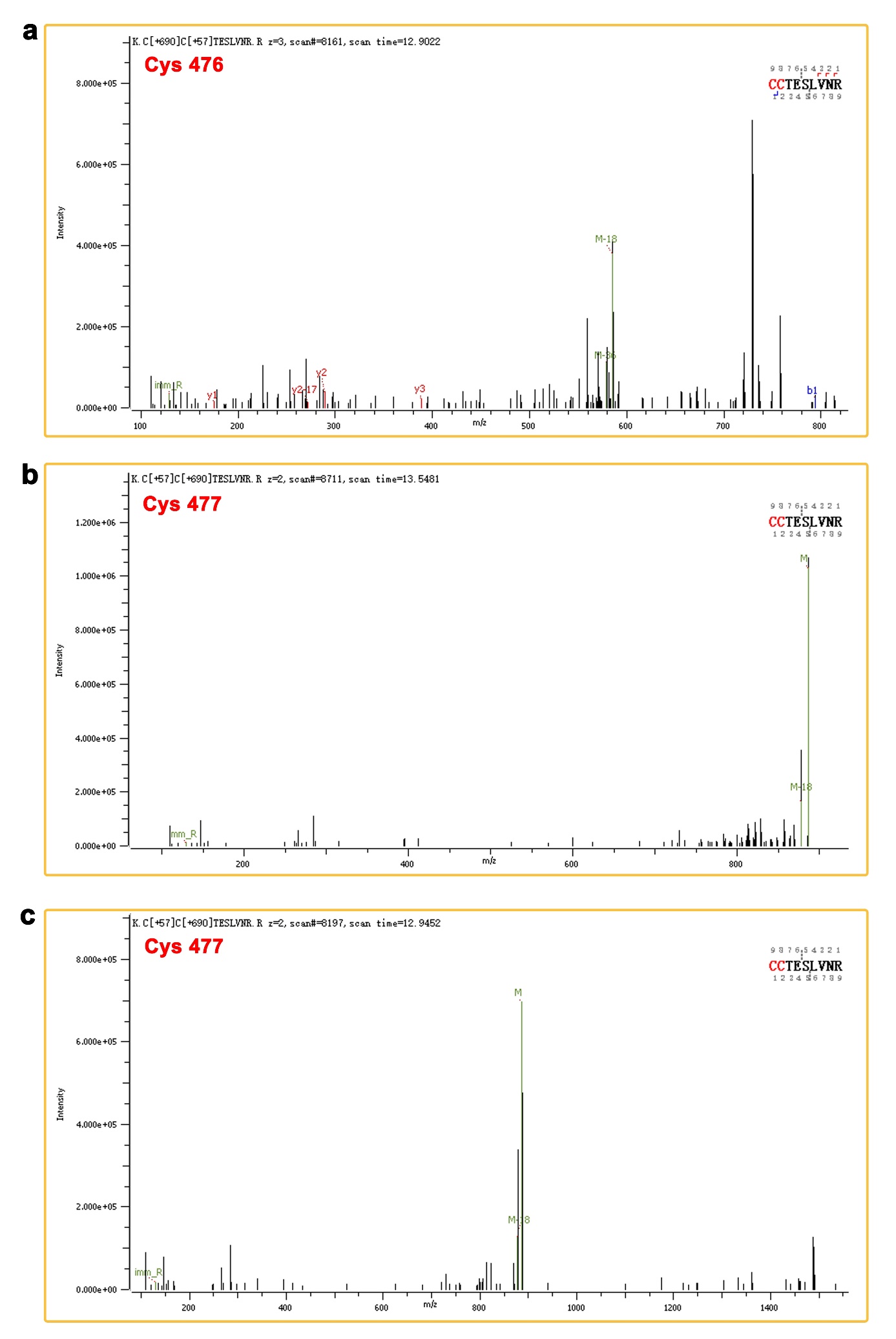
**

**Figure S24.** a) The secondary mass spectra of the sequence of CCTESLVNR (Cys476). b) The secondary mass spectra of the sequence of CCTESLVNR (Cys477). c) The secondary mass spectra of the sequence of CCTESLVNR (Cys477). The measurement accuracy for the corresponding fragments was < 0.02 Da (y ion series; y1–y8).

**
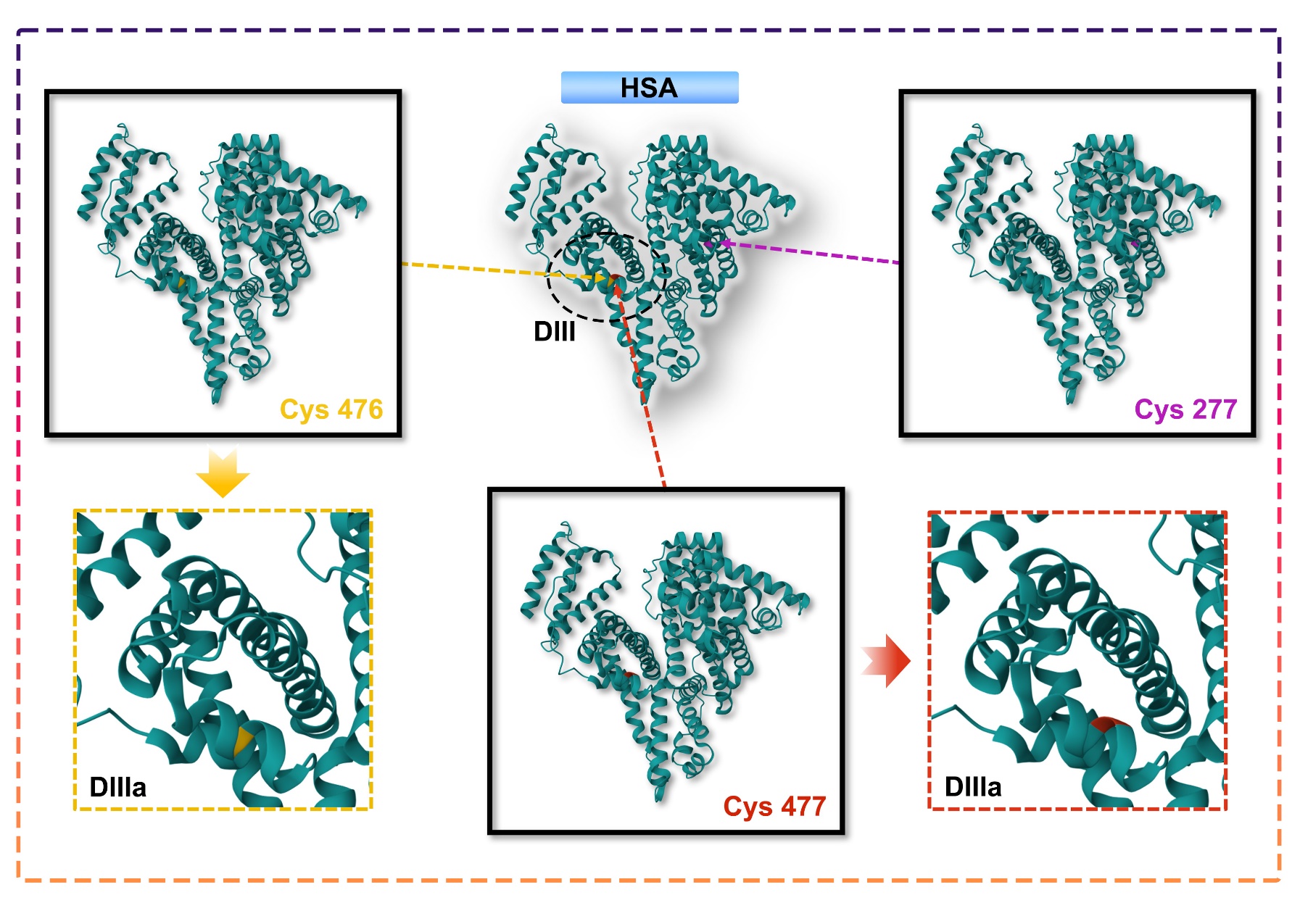
**

**Figure S25.** Distribution of cysteine sites recognized by proteomics. Protein structures were generated by the Protein Data Bank (PDB).


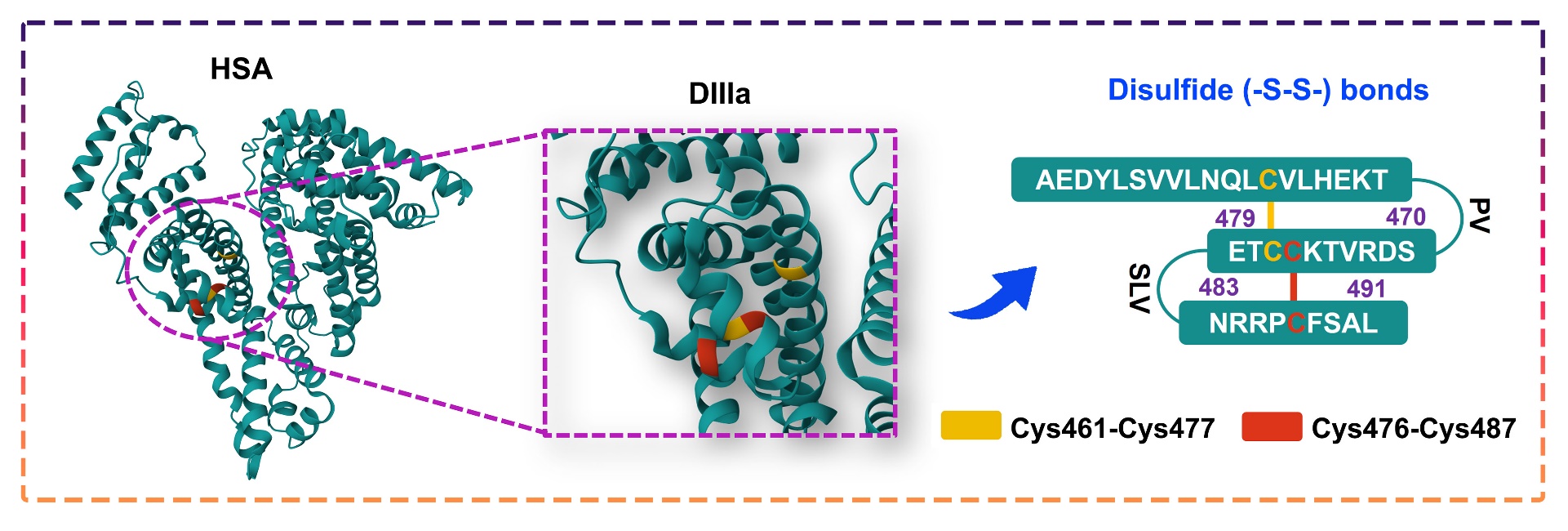


**Figure S26.** Distribution of credible cysteine sites and their corresponding disulfide bonds recognized by proteomics. Protein structures were generated by the Protein Data Bank (PDB).

**
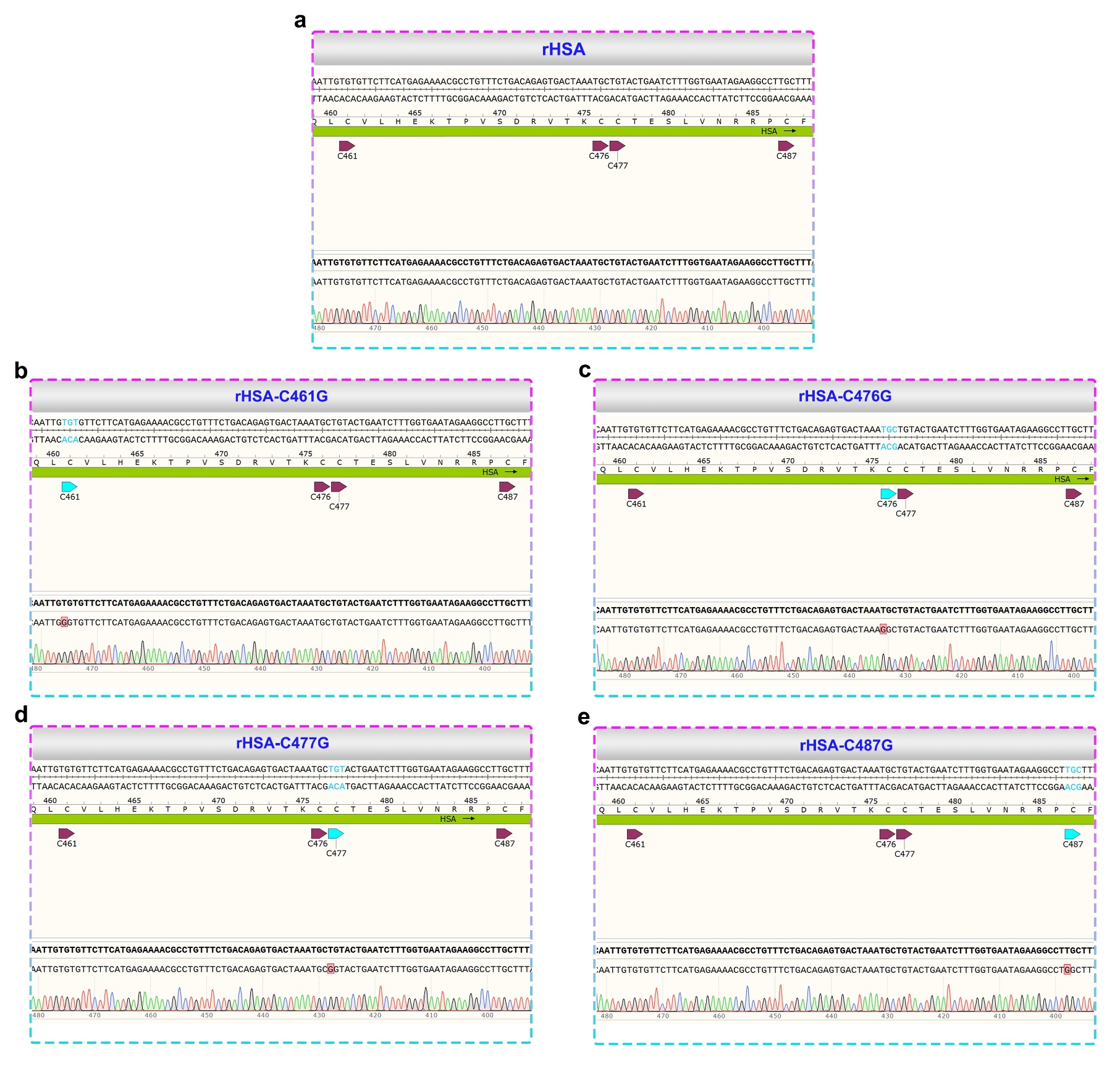
**

**Figure S27.** DNA sequencing results of different mutant proteins, including a) rHSA, b) rHSA-C461G, c) rHSA-C476G, d) rHSA-C477G, and e) rHSA-C487G.

**
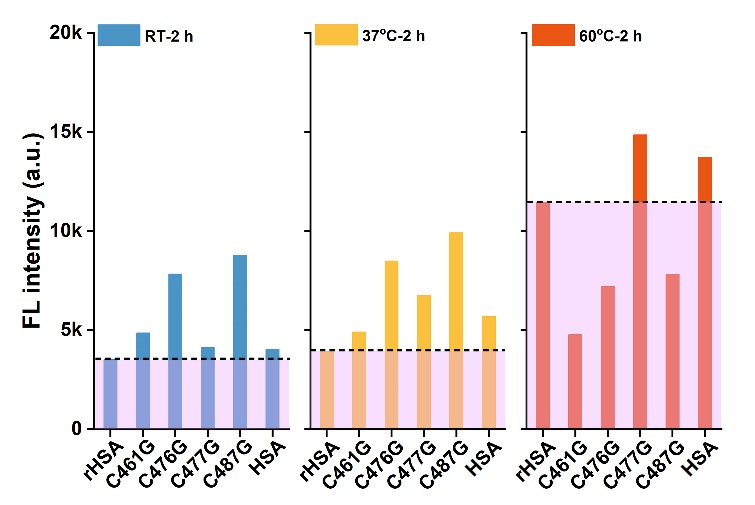
**

**Figure S28.** NIR-II brightness analysis of recombinant HSA and C7-1080 dyes with different site mutations under different reaction conditions, including RT-2 h, 37^o^C-2 h, and 60^o^C-2 h.


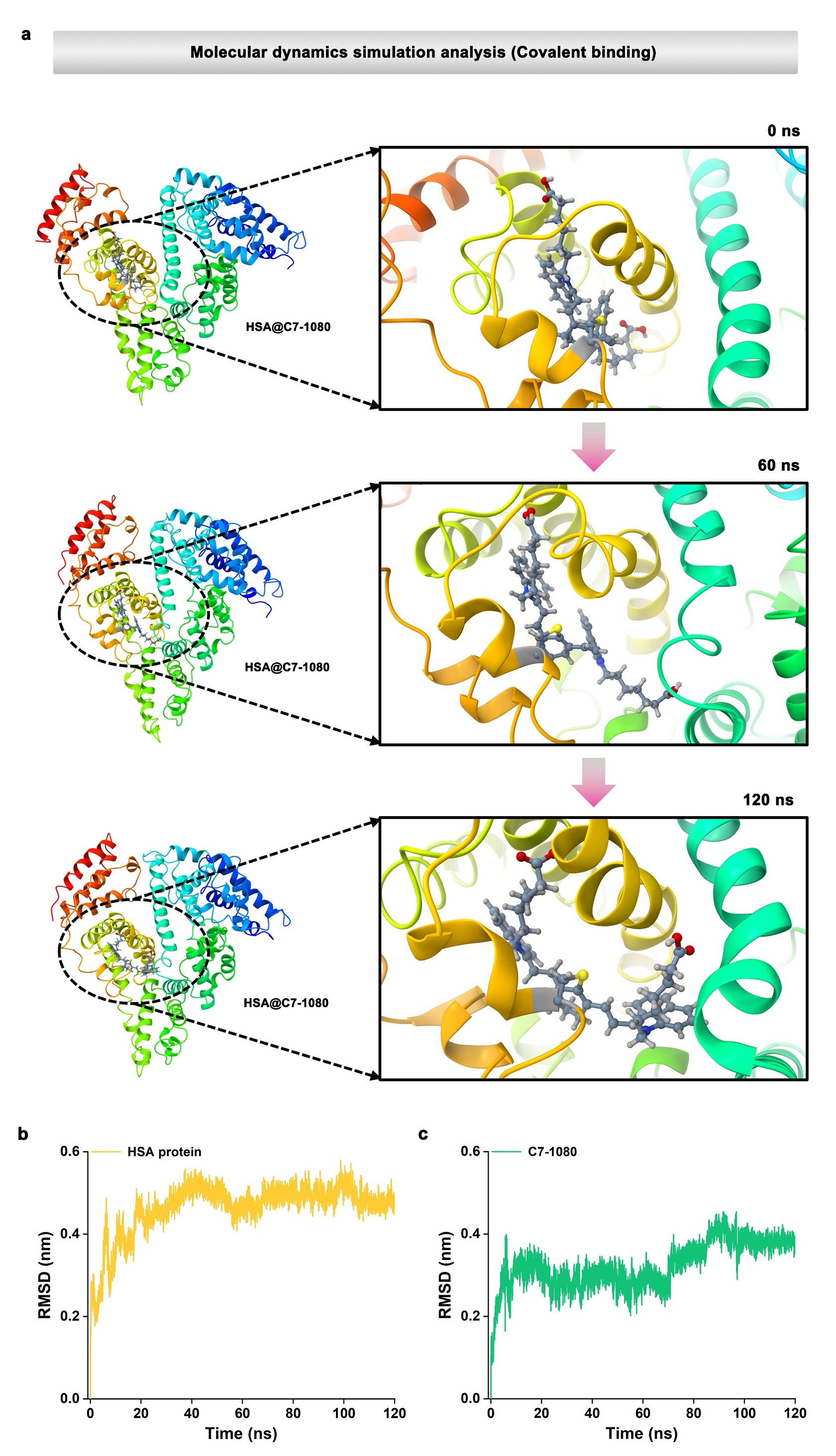


**Figure S29.** a) Molecular dynamics simulation of covalently bound HSA@C7-1080 at different specific times. RMSD of b) HSA protein and C7-1080 dye during the molecular dynamic simulation as a function of time.

**
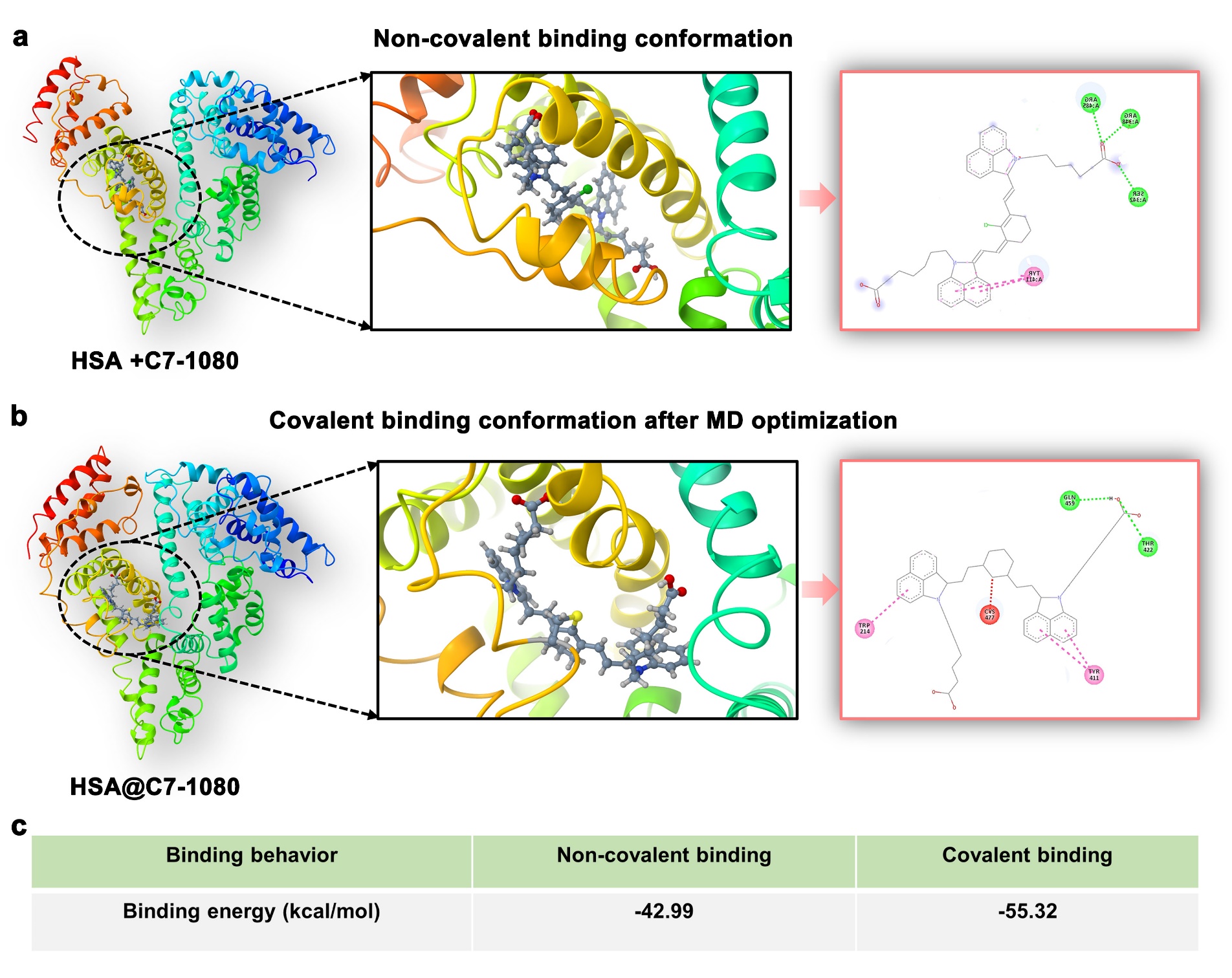
**

**Figure S30.** a) Non-covalent binding conformation of HSA and C7-1080. b) Covalent binding conformation after molecular dynamics (MD) optimization of HSA and C7-1080 (120 ns). c) Statistics of non-covalent binding energy and covalent binding energy between HSA and C7-1080.


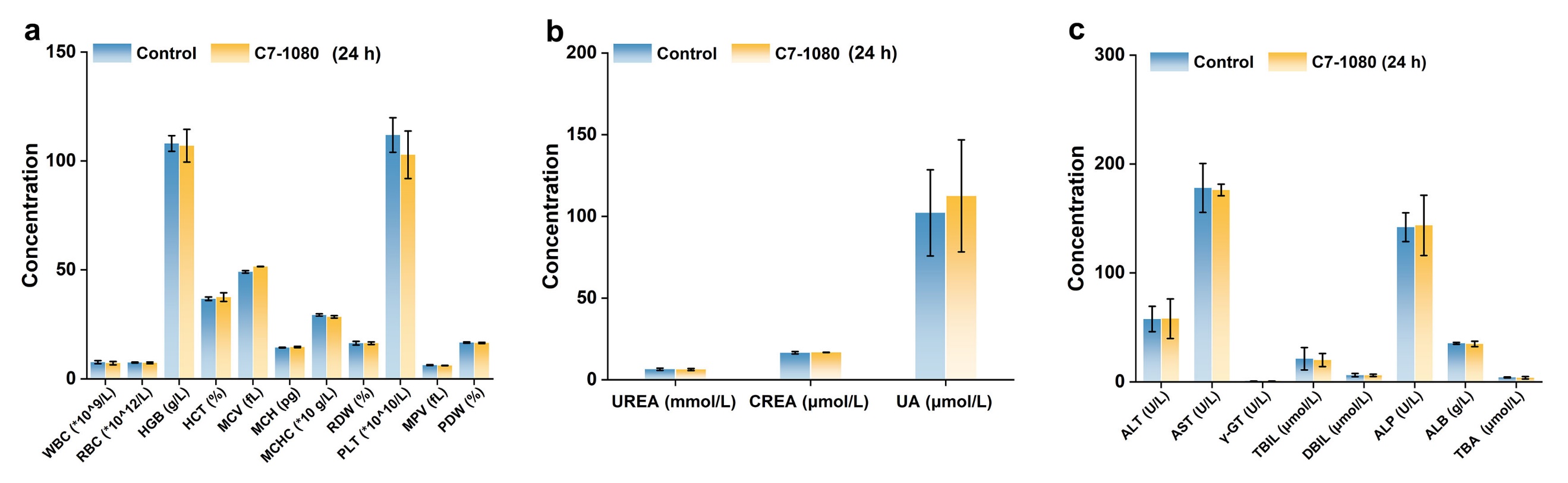


**Figure S31.** a) Blood routine indexes of mice after tail vein injection of the C7-1080 probes for 24 h. b) Hepatic and c) renal function indexes of mice after tail vein injection of the C7-1080 probes for 24 h.

**
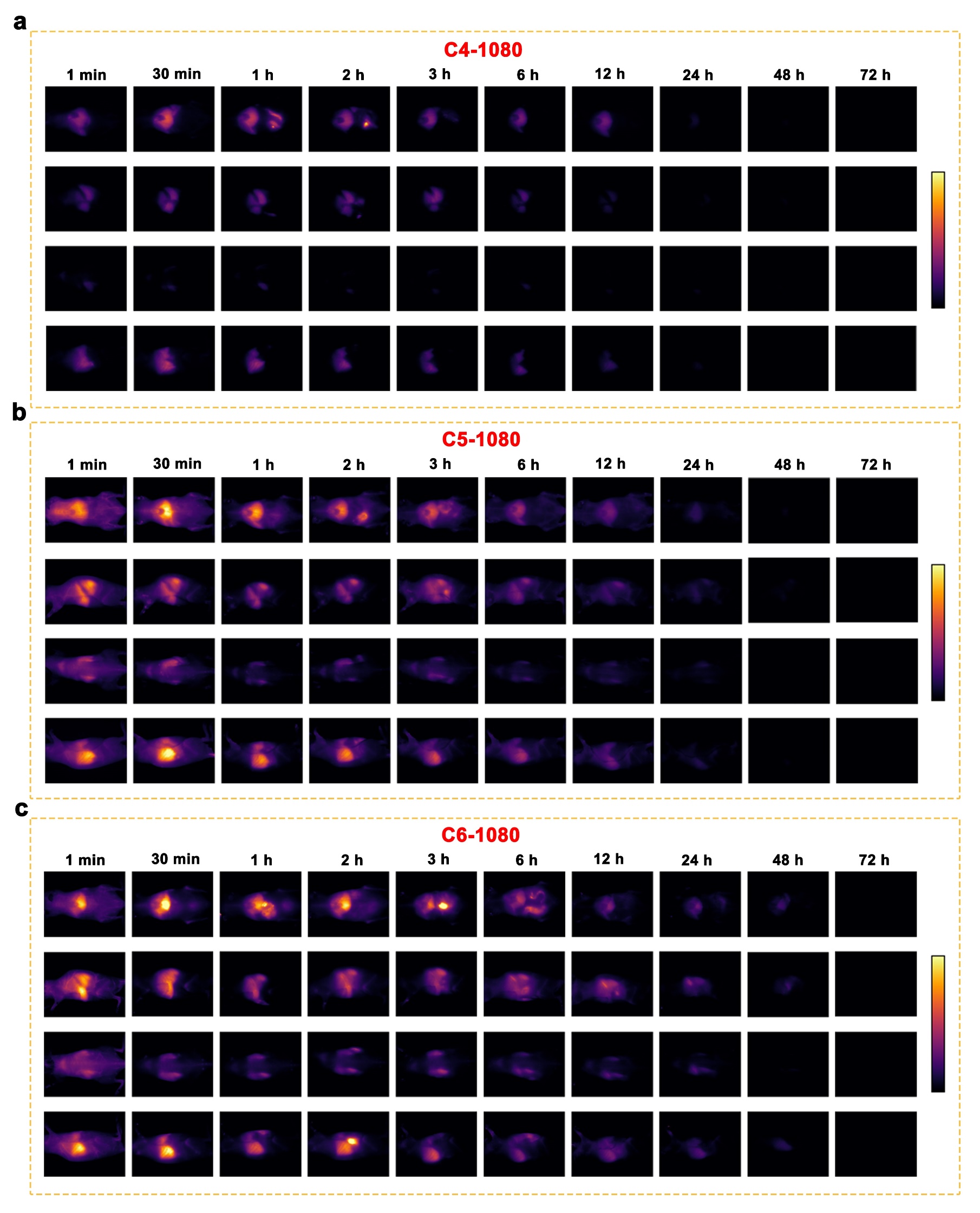
**

**Figure S32.** Metabolic behavior of mice at different time points after tail vein injection of a) C4-1080, b) C5-1080, c) C6-1080.

**
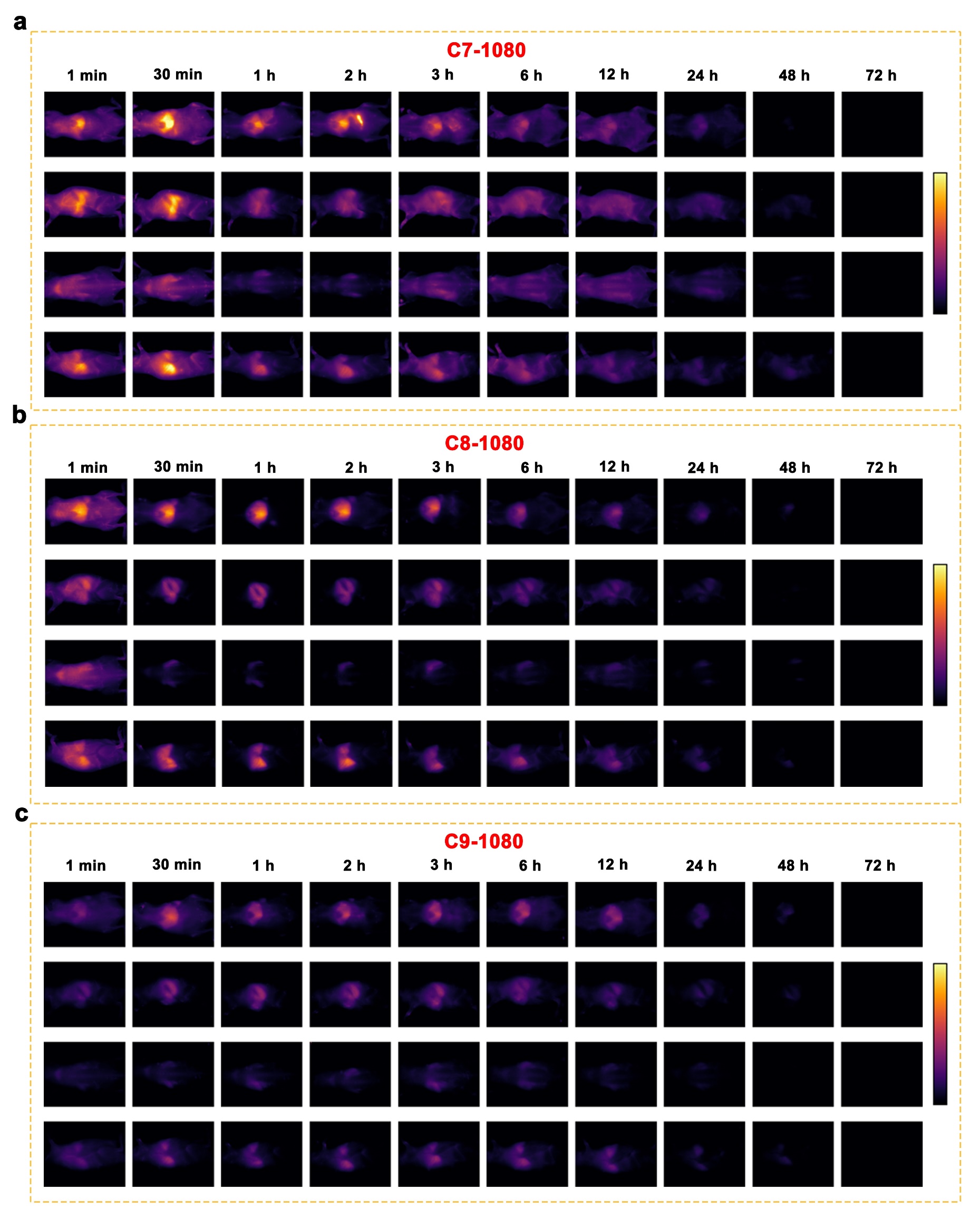
**

**Figure S33.** Metabolic behavior of mice at different time points after tail vein injection of a) C7-1080, b) C8-1080, c) C9-1080.

**
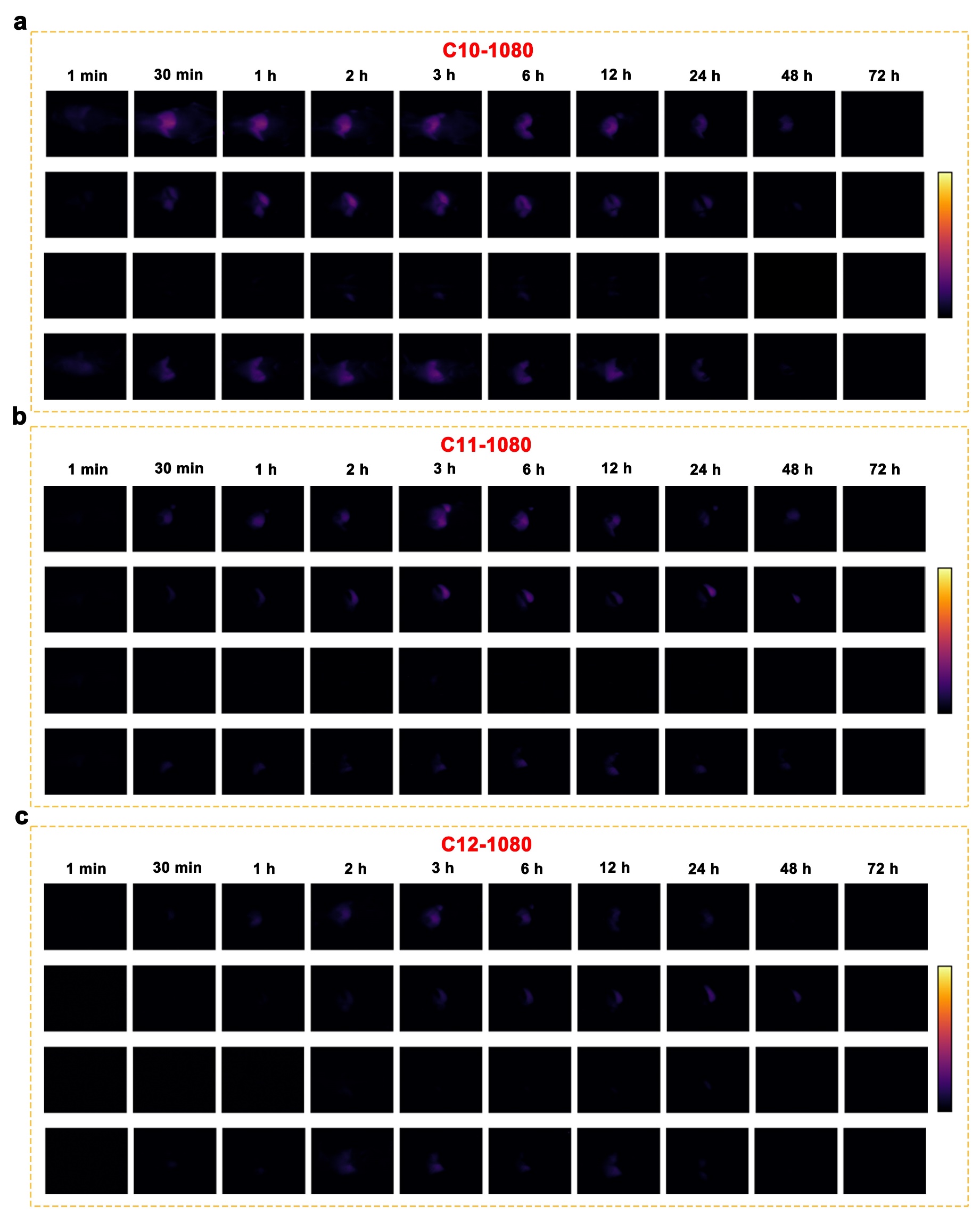
**

**Figure S34.** Metabolic behavior of mice at different time points after tail vein injection of a) C10-1080, b) C11-1080, c) C12-1080.


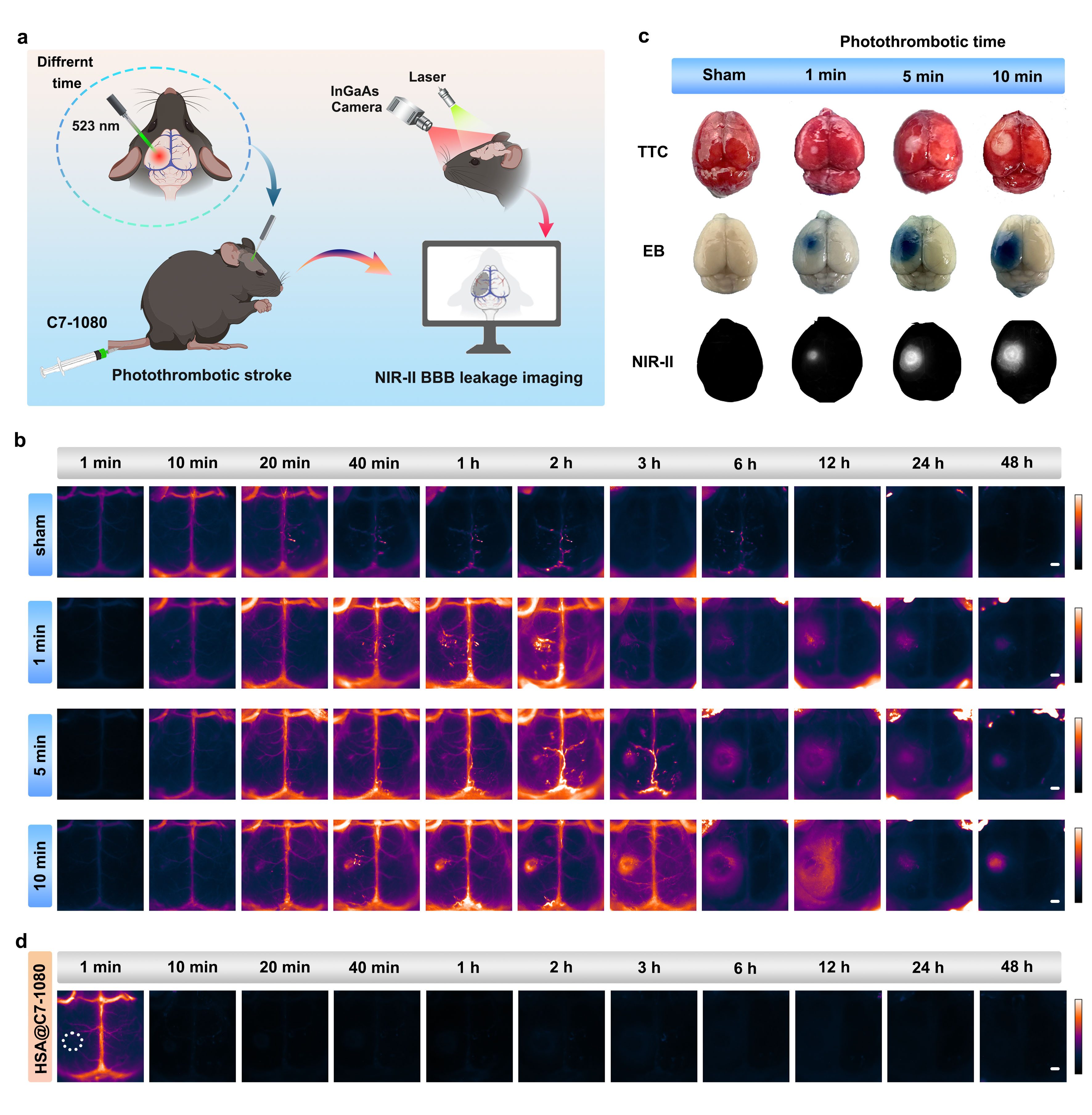


**Figure S35.** a) Schematic of targeted imaging of blood-brain barrier (BBB) disruption based on C7-1080 dye. b) Dynamic BBB disruption imaging of sham/stroke mice with different photothrombotic time after tail vein injection of C7-1080 dyes. c) 2,3,5-triphenyl tetrazolium chloride (TTC) staining, Evans blue (EB) staining, and NIR-II fluorescence imaging of the ex-vivo whole brain of sham/stroke mice under different photothrombotic time. d) Dynamic BBB disruption imaging of stroke mice after tail vein injection of HSA@C7-1080 dyes. Scale bar = 1 mm.

**Note:** according to the difference of photothrombotic time and the degree of brain injury, the stroke models were divided into four groups: sham group (photothrombotic for 0 min); mild stroke group (photothrombotic for 1 min), moderate stroke group (photothrombotic for 5 min); severe stroke group (photothrombotic for 10 min).

**
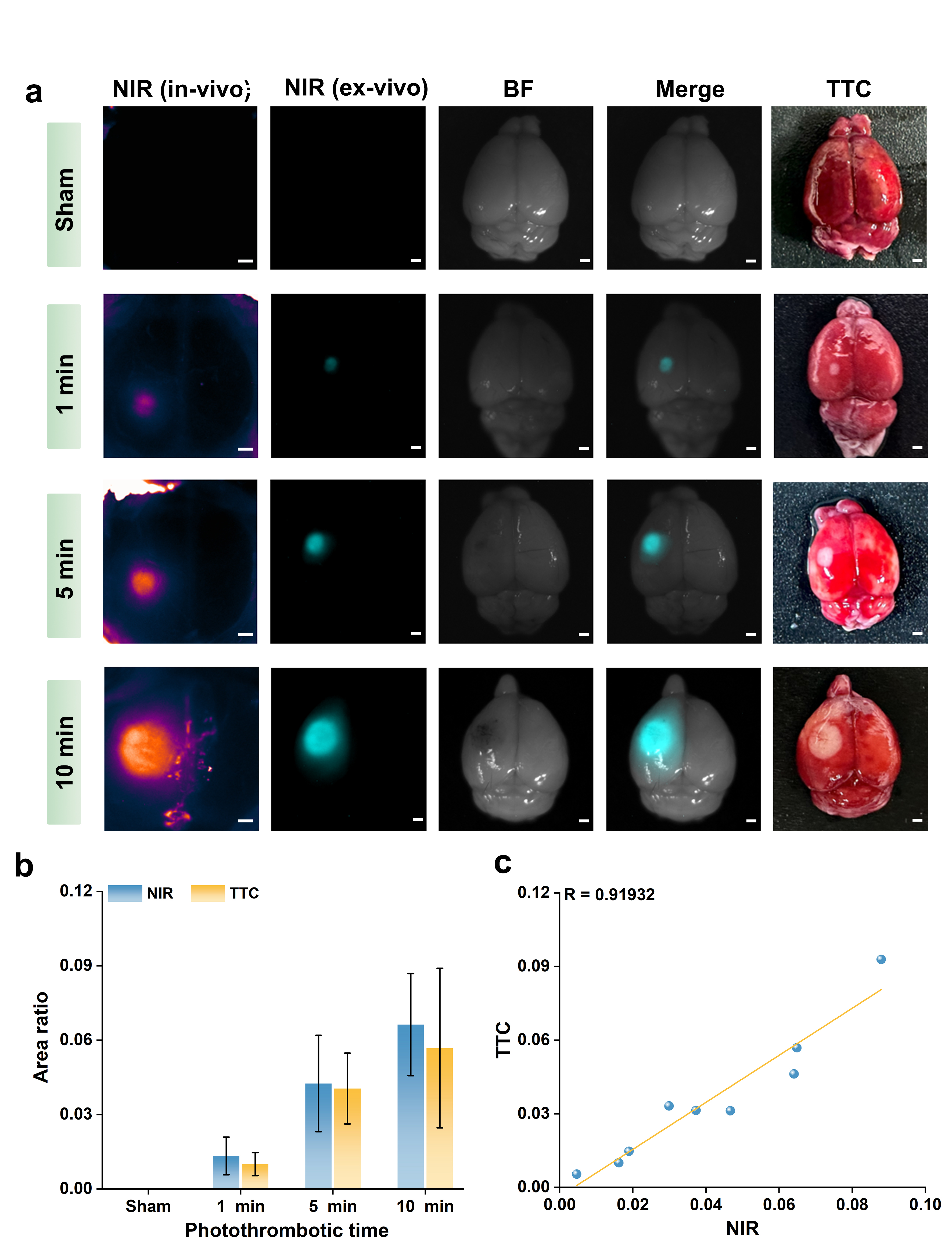
**

**Figure S36.** a) TTC staining and in-vivo/vitro NIR imaging of mouse brain tissue with different degrees of stroke. b) TTC staining area ratio and NIR fluorescence labeling area ratio of brain tissue of mice with different stroke degree. c) Correlation analysis of TTC staining and NIR fluorescence imaging in brain tissue of mice with different degrees of stroke (BF: bright field imaging; TTC: 2,3,5-triphenyl tetrazolium chloride). Scale bar = 1 mm.

**Note:** TTC staining and in-vivo/vitro NIR imaging of mouse brain tissue with different degrees of stroke were performed after 48 h tail vein injection of C7-1080 dyes immediately after modeling. Specific TTC staining procedures were detailed in the "Methods" section of the Supporting Information. Besides, the correlation between TTC staining and NIR fluorescence labeling was statistically calculated based on the ratio of TTC staining area to the whole brain area and the ratio of NIR fluorescence labeling to the whole brain area in mice with different degrees of stroke. For the TTC staining area, the target contour was manually circled according to the naked eye observation, and then calculated using Image J software. For the NIR fluorescence labeling area, the cutoff value of ex-vivo NIR fluorescence labeling was 1500~66535, and then the target area was calculated using Image J software.


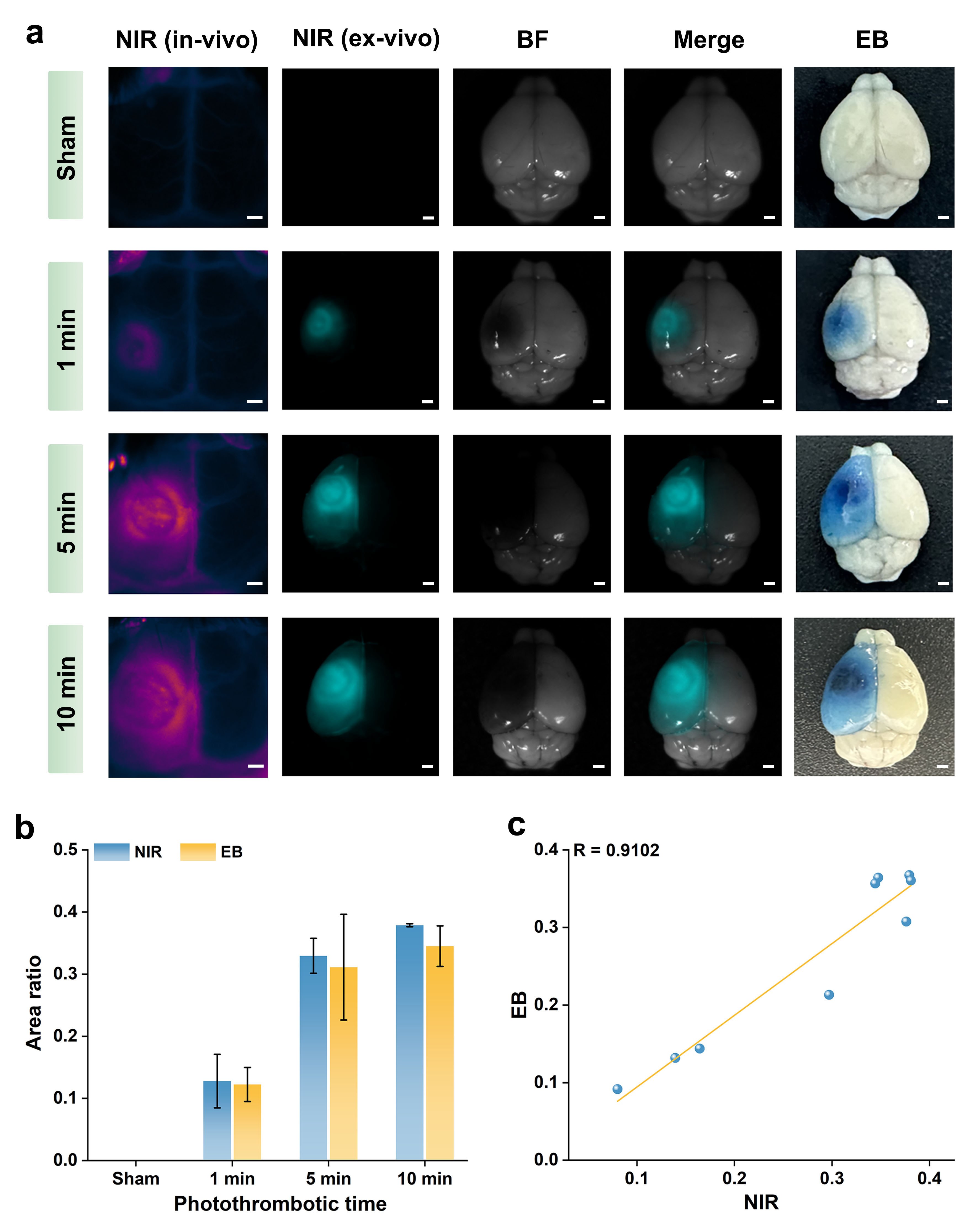


**Figure S37.** a) EB staining and in-vivo/vitro NIR imaging of mouse brain tissue with different degrees of stroke. b) EB staining area ratio and NIR fluorescence labeling area ratio of brain tissue of mice with different stroke degree. c) Correlation analysis of EB staining and NIR fluorescence imaging in brain tissue of mice with different degrees of stroke (BF: bright field imaging; EB: Evans blue). Scale bar = 1 mm.

**Note:** EB staining and in-vivo/vitro NIR imaging of mouse brain tissue with different degrees of stroke were performed after 3 h of tail vein injection of EB/C7-1080 dyes immediately after modeling. Specific EB staining procedures were detailed in the "Methods" section of the Supporting Information. Besides, the correlation between EB staining and NIR fluorescence labeling was statistically calculated based on the ratio of EB staining area to the whole brain area and the ratio of NIR fluorescence labeling to the whole brain area in mice with different degrees of stroke. For the EB staining area, the target contour was manually circled according to the naked eye observation, and then calculated using Image J software. For the NIR fluorescence labeling area, the cutoff value of ex-vivo NIR fluorescence labeling was 2000~66535, and then the target area was calculated using Image J software.

**
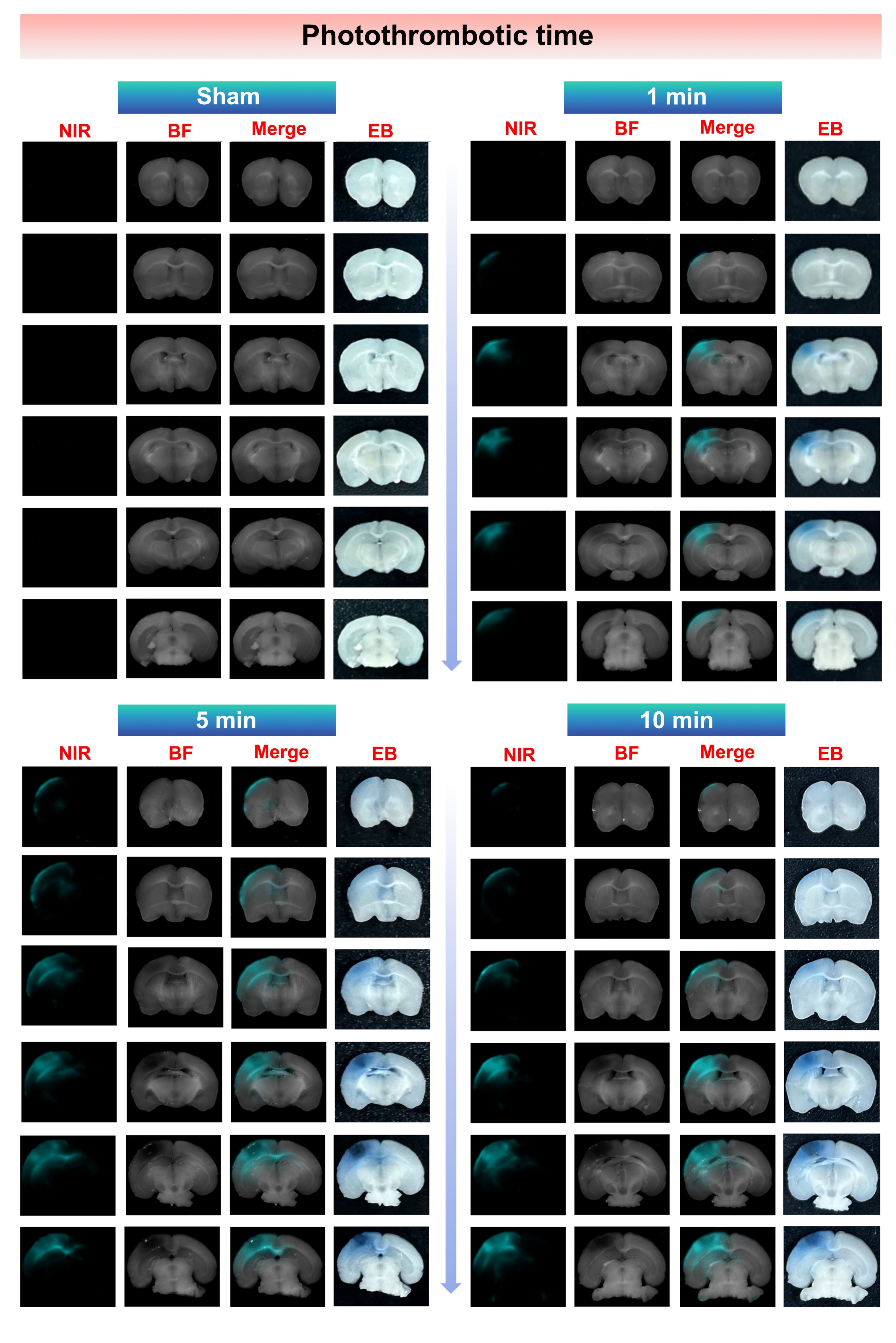
**

**Figure S38.** Brain tissue section imaging of sham/stroke mice with different photothrombotic times after tail vein injection of C7-1080/EB dyes (BF: bright field imaging; EB: Evans Blue).


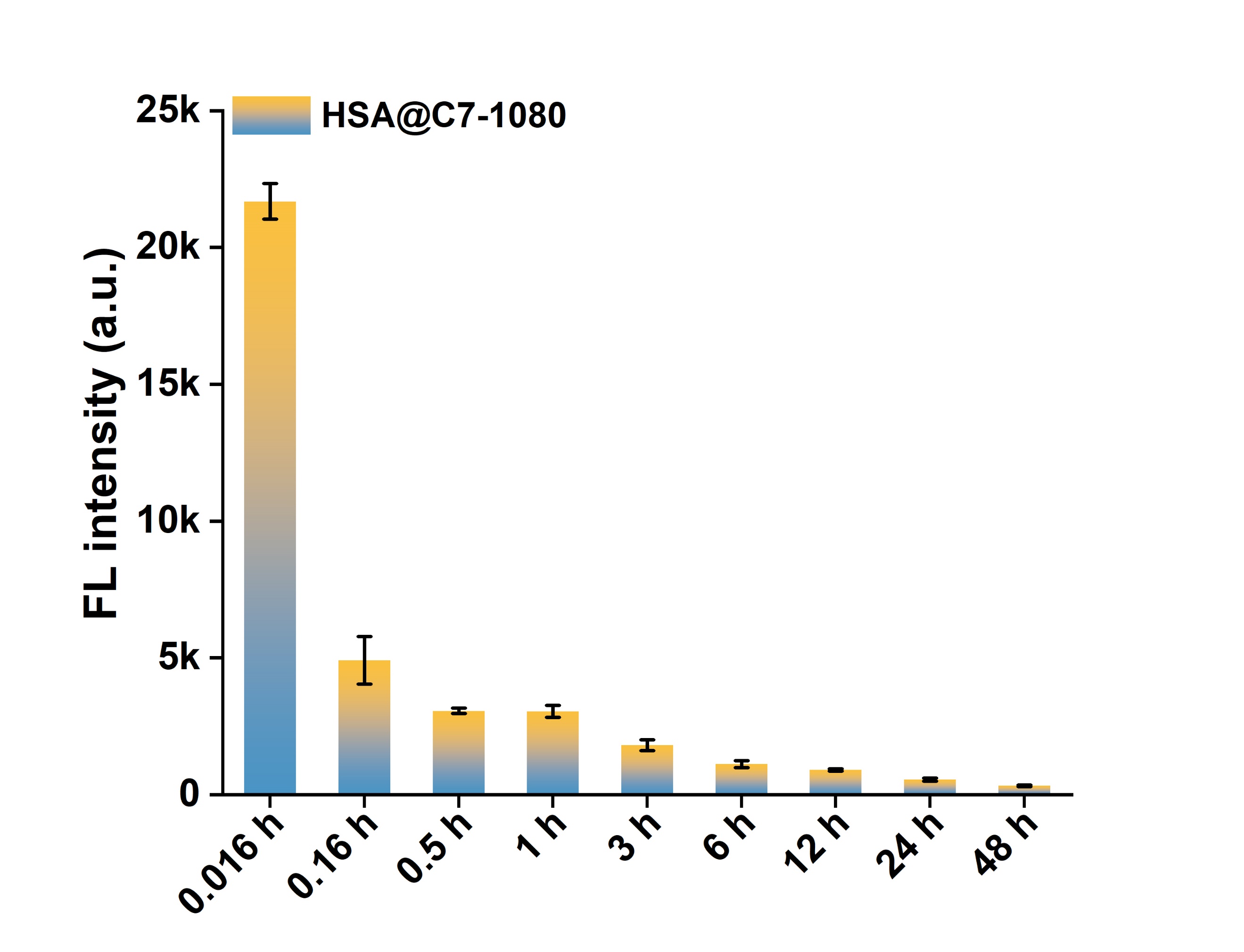


**Figure S39.** Blood brightness analysis at different time points after tail vein injection of HSA@C7-1080 dye.

**
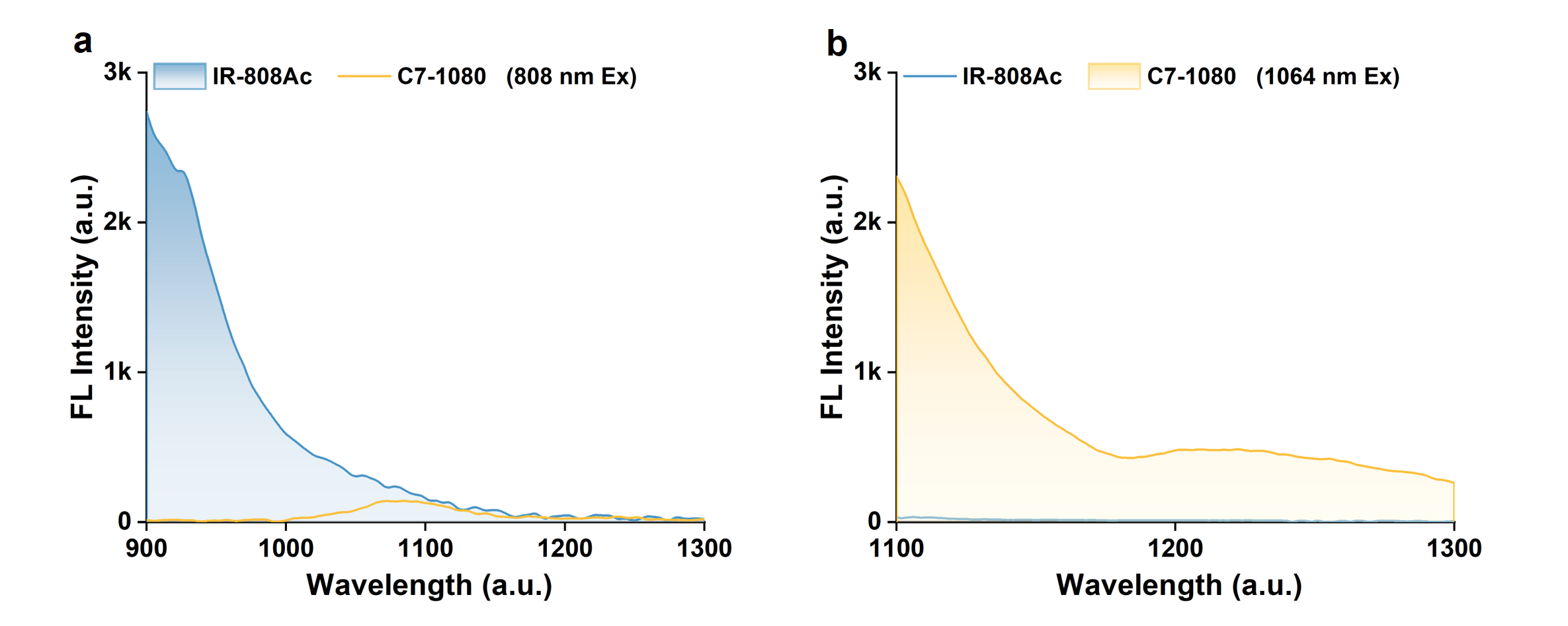
**

**Figure S40.** Fluorescence spectra of IR-808Ac and C7-1080 dyes under a) 808 nm b) 1064 nm excitation.

**
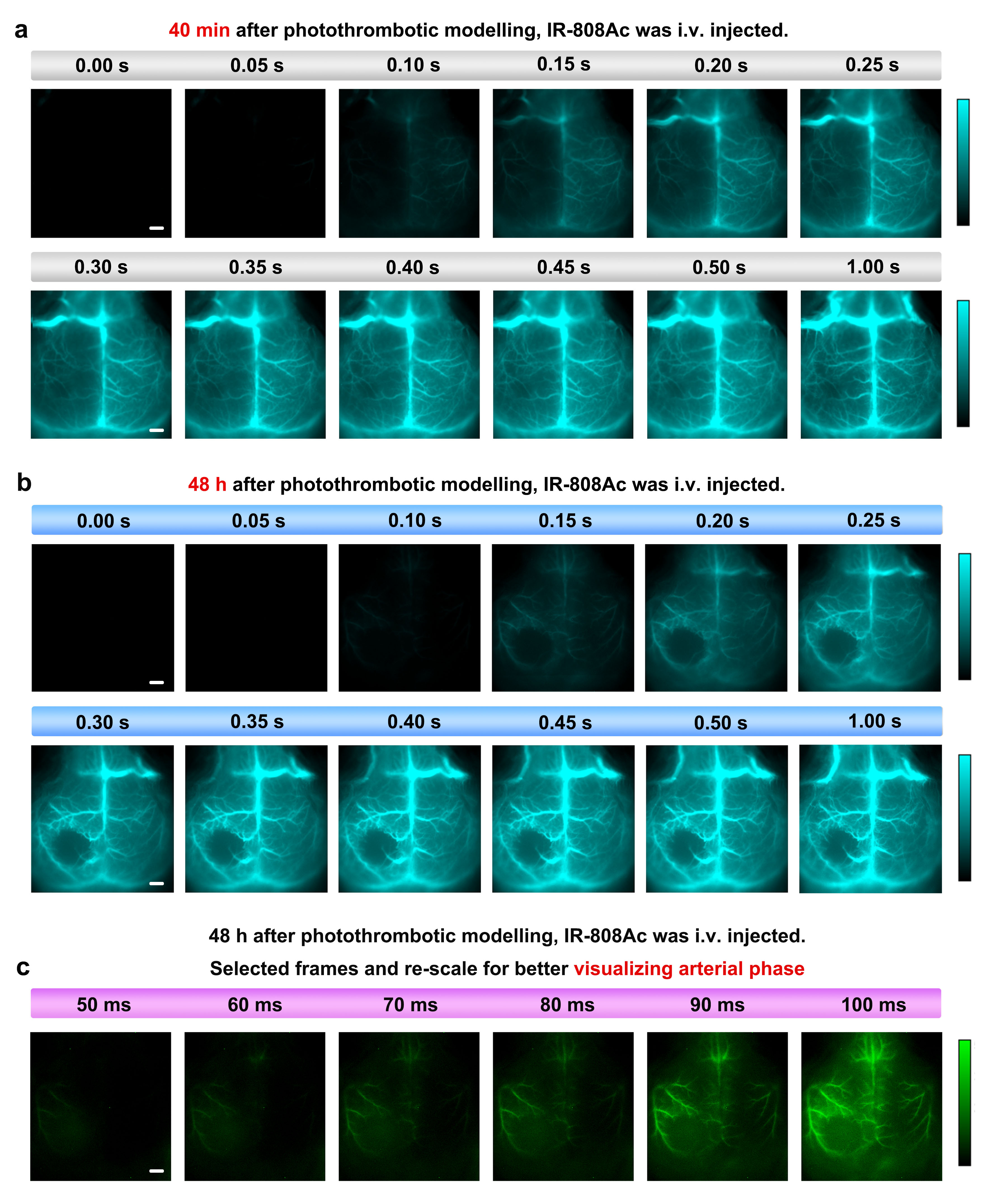
**

**Figure S41.** a) Dynamic cerebral vascular imaging of stroke mice after photothrombotic modeling for 40 min by the tail vein injection of the IR-808Ac dyes. b) Dynamic cerebral vascular imaging of stroke mice after photothrombotic modeling for 48 h by the tail vein injection of the IR-808Ac dyes. c) Dynamic arterial imaging of stroke mice after photothrombotic modeling for 48 h by the tail vein injection of the IR-808Ac dyes. Scale bar = 1 mm.


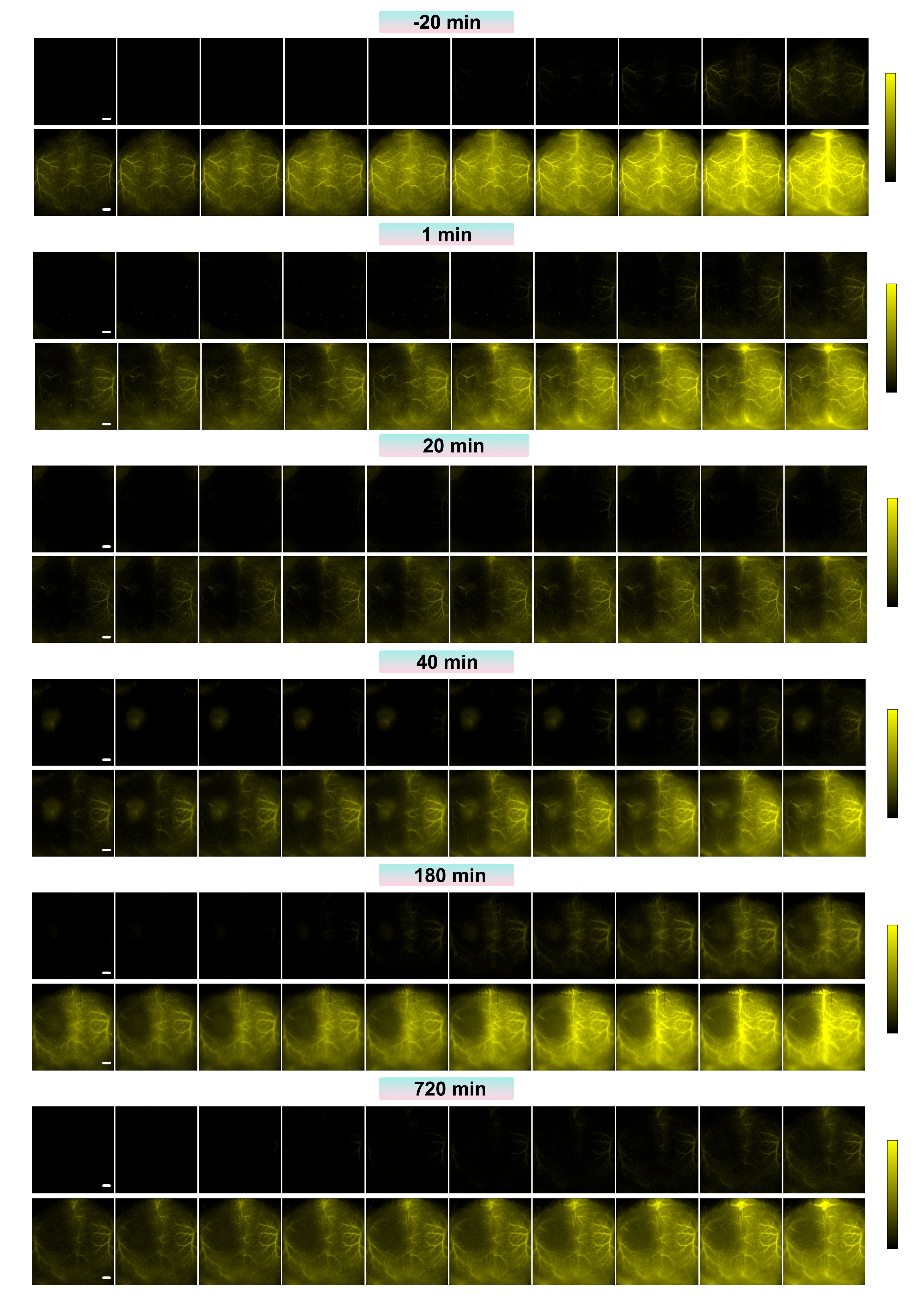


**Figure S42.** Cerebral arterial phase angiography of stroke mice after photothrombotic modeling at different time points by the tail vein injection of the IR-808Ac dyes. Scale bar = 1 mm.

**Note:** Due to multiple injections of the IR-808Ac probe for cerebral vascular imaging within a short time interval, leading to partial IR-808Ac-labeled BBB disruption imaging signal interference near the injured area.


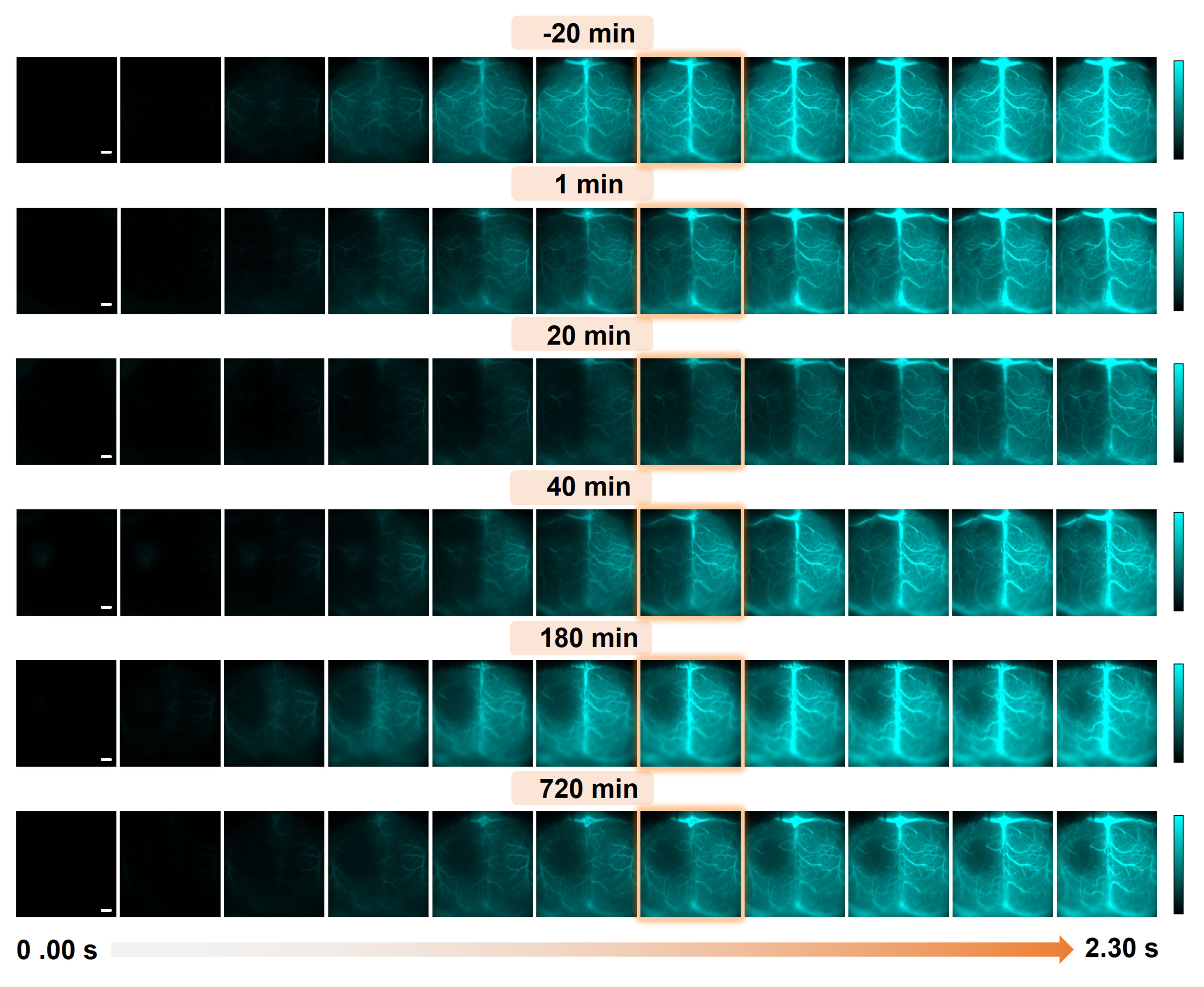


**Figure S43.** Cerebral venous phase angiography of stroke mice after photothrombotic modeling at different time points by the tail vein injection of the IR-808Ac dyes. Scale bar = 1 mm.


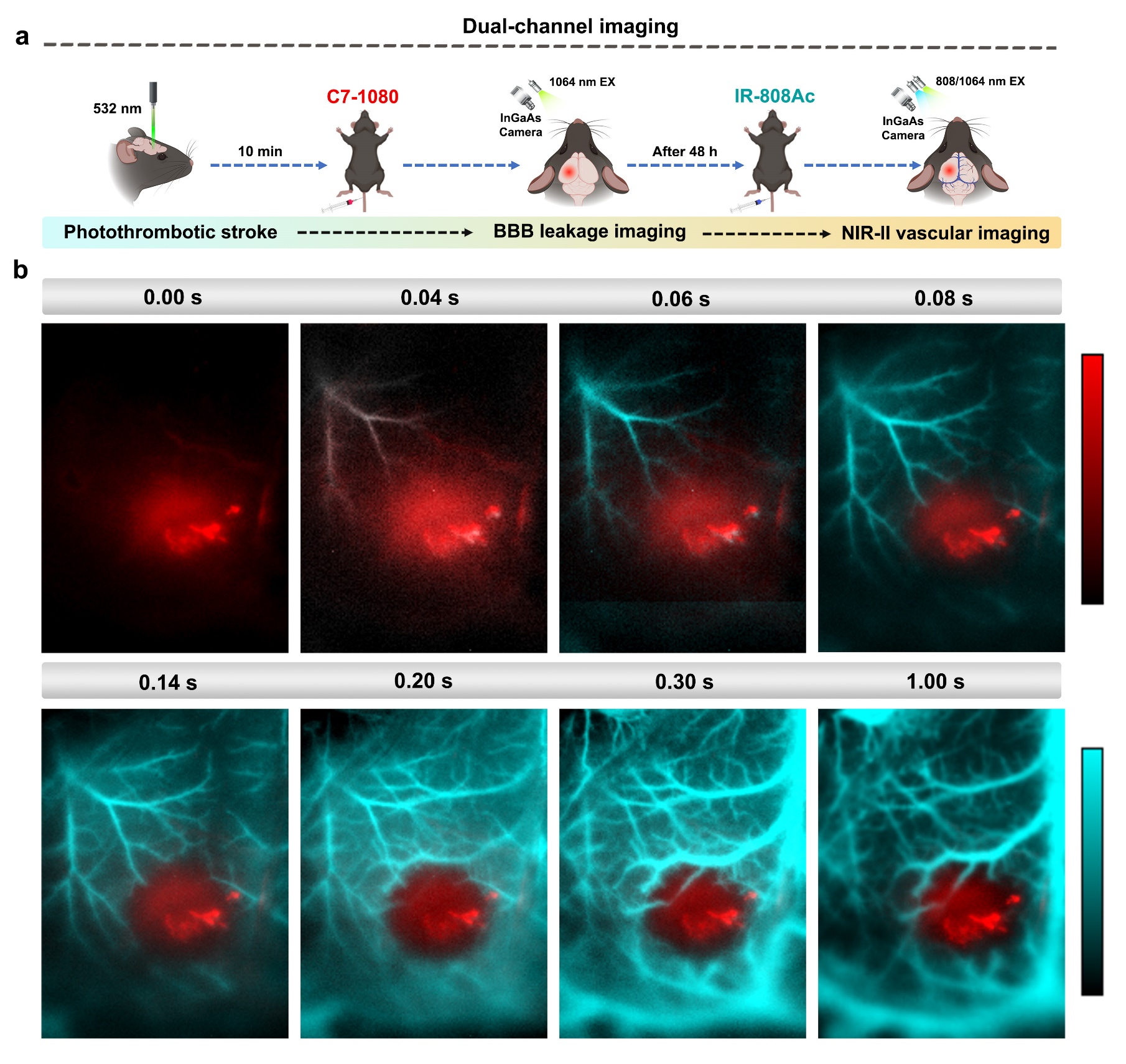


**Figure S44.** a) Dual-channel imaging schematic of the blood-brain barrier (BBB) and cerebral vascular based on the IR-808Ac and C7-1080 dyes. b) Dual-channel images of BBB and cerebral vessels at different time points based on IR-808Ac and C7-1080 dyes.

**NMR spectra and** **MS spectra of the synthesized compounds**

**
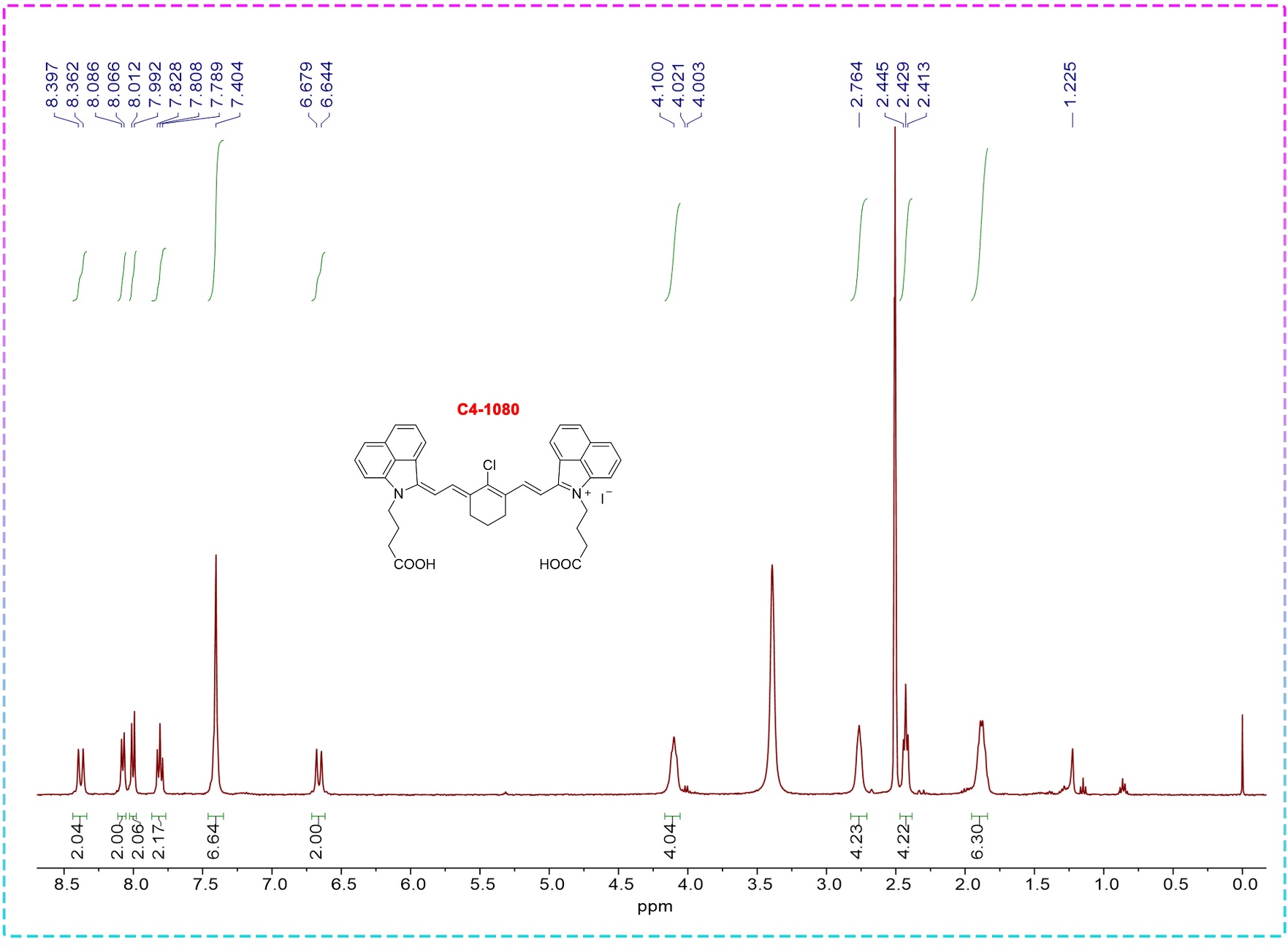
**

**^1^H-NMR spectrum of C4-1080 in DMSO-d6.**

**
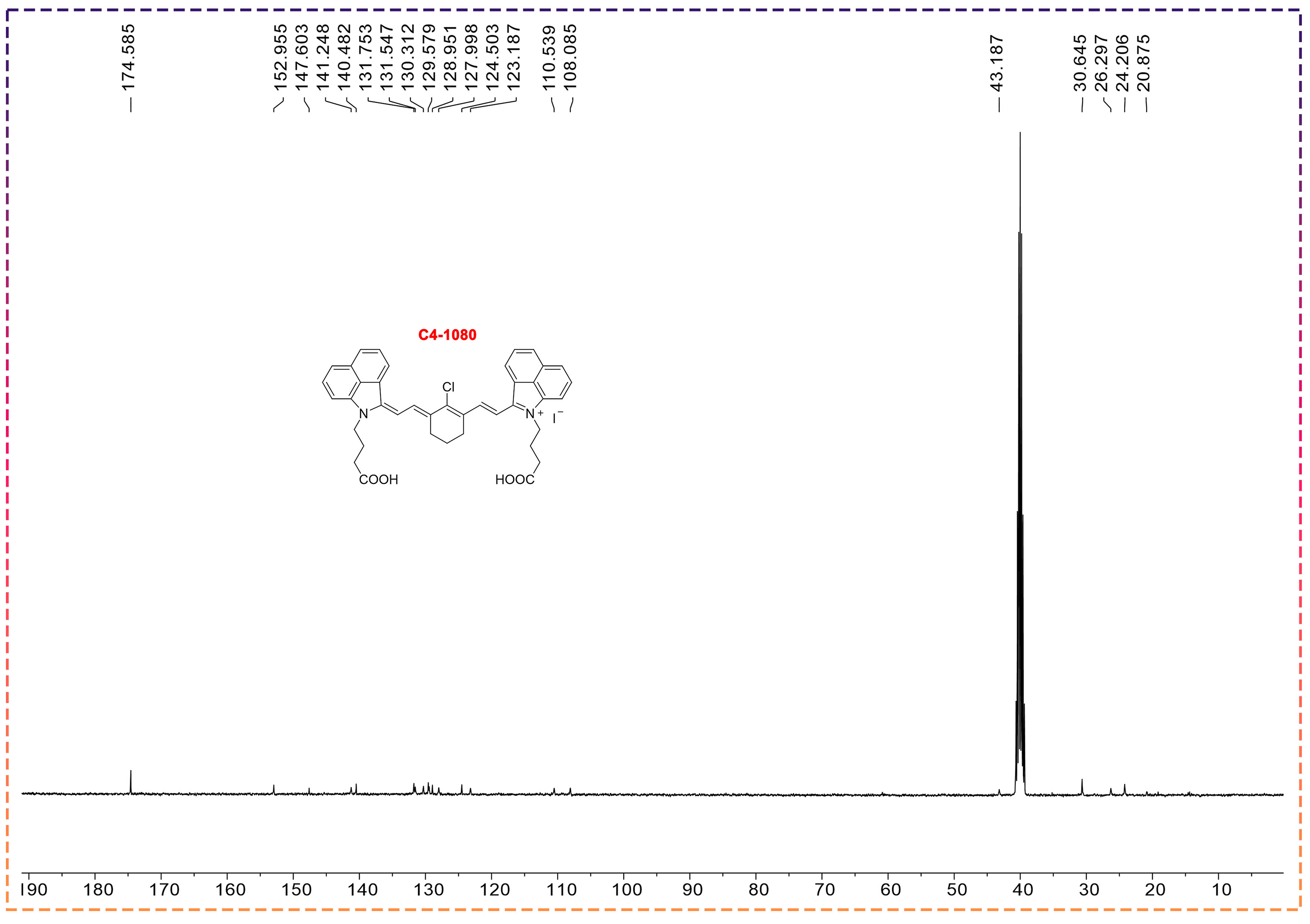
**

**^13^C NMR spectrum of C4-1080 in DMSO-d6.**


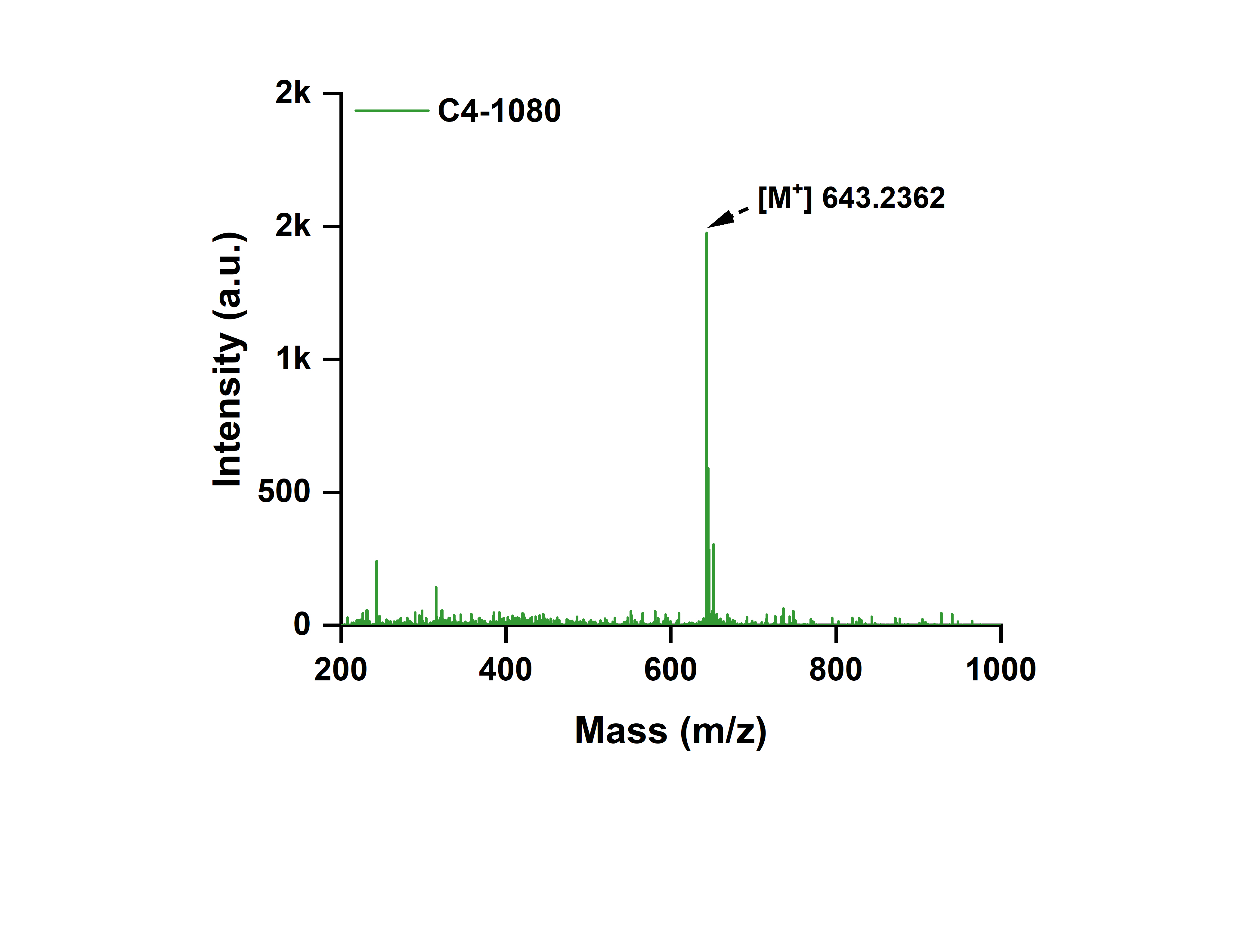


**LC-HRMS spectra of the C4-1080.**

**
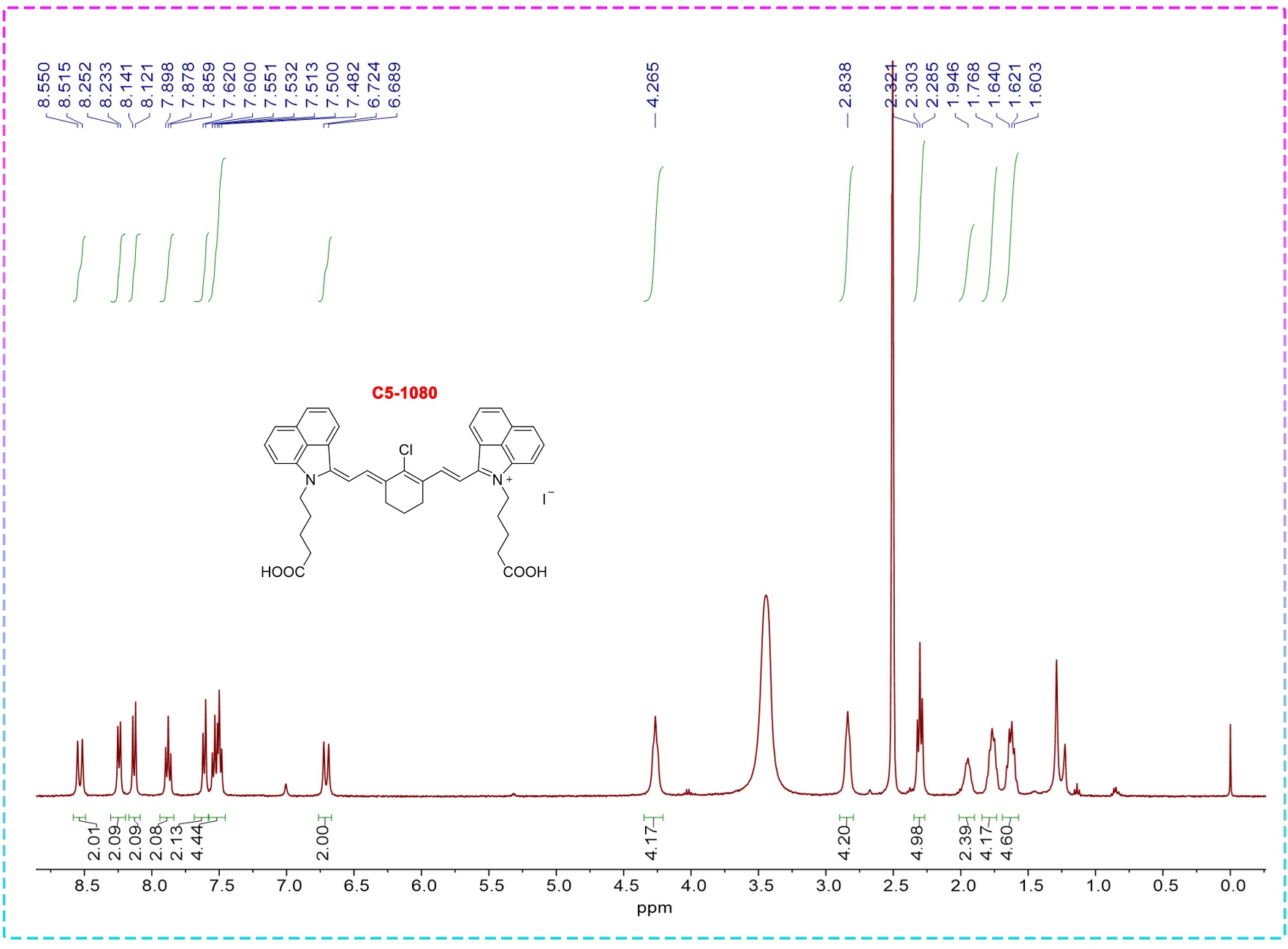
**

**^1^H-NMR spectrum of C5-1080 in DMSO-d6.**

**
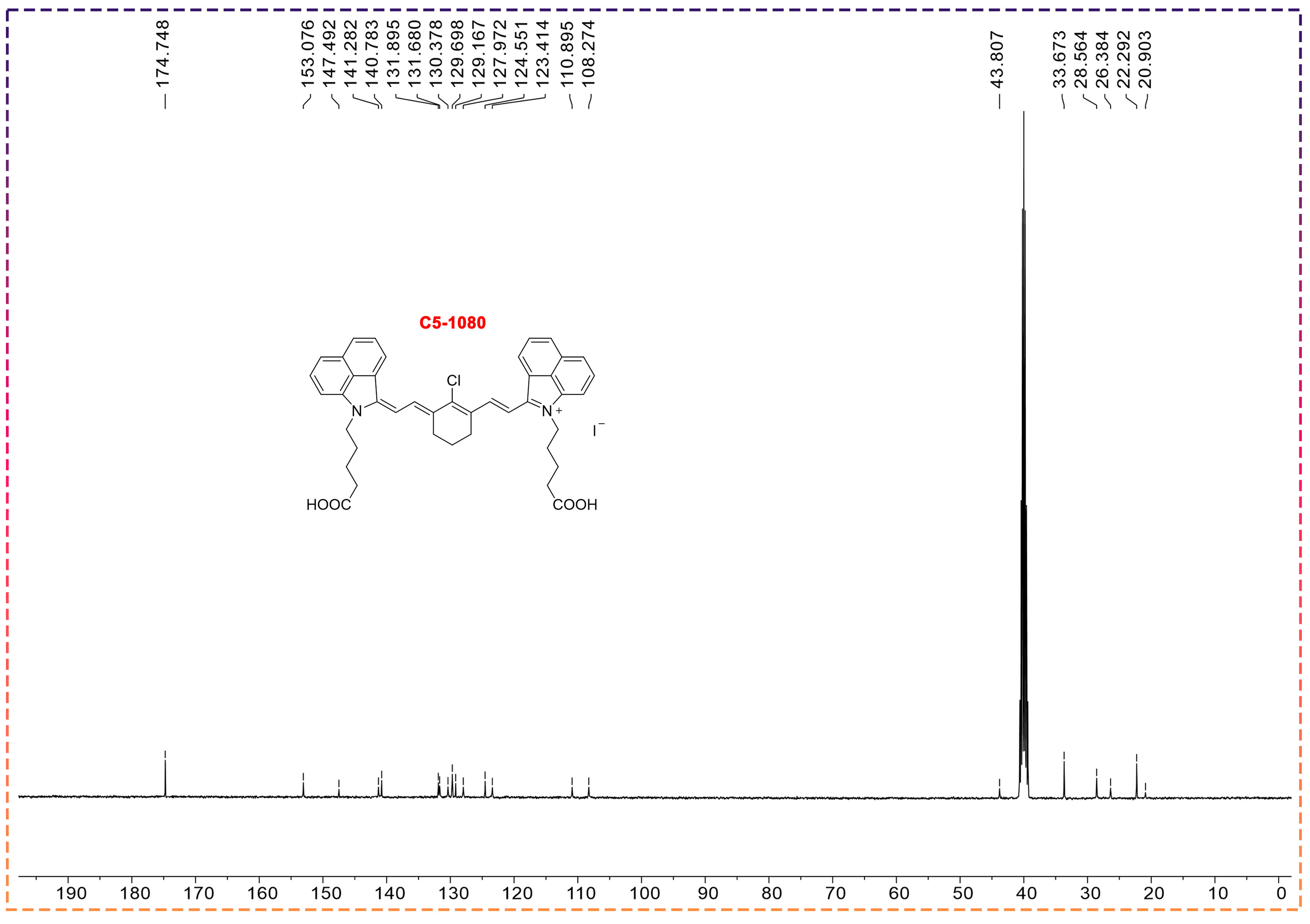
**

**^13^C NMR spectrum of C5-1080 in DMSO-d6.**

**
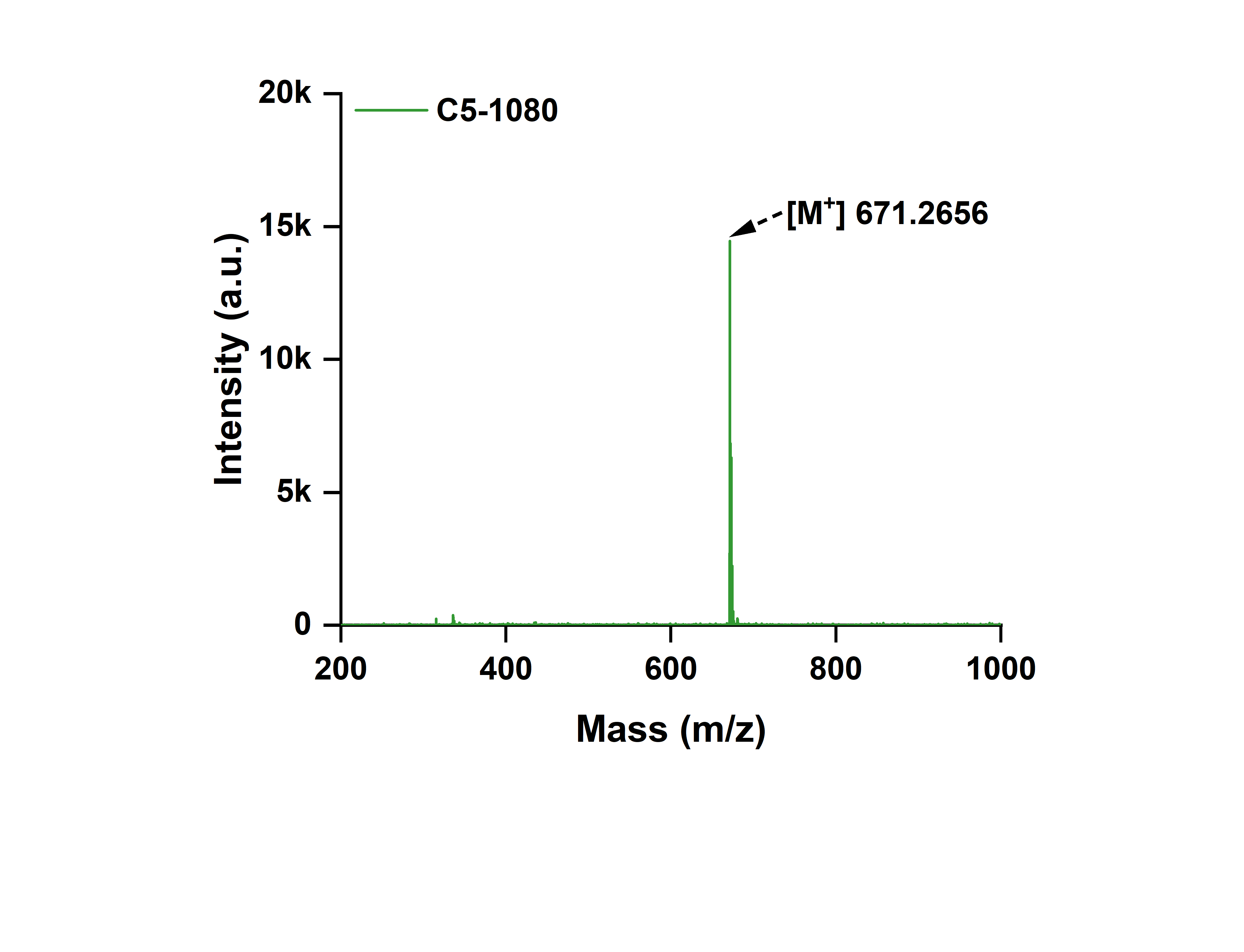
**

**LC-HRMS spectra of the C5-1080.**

**
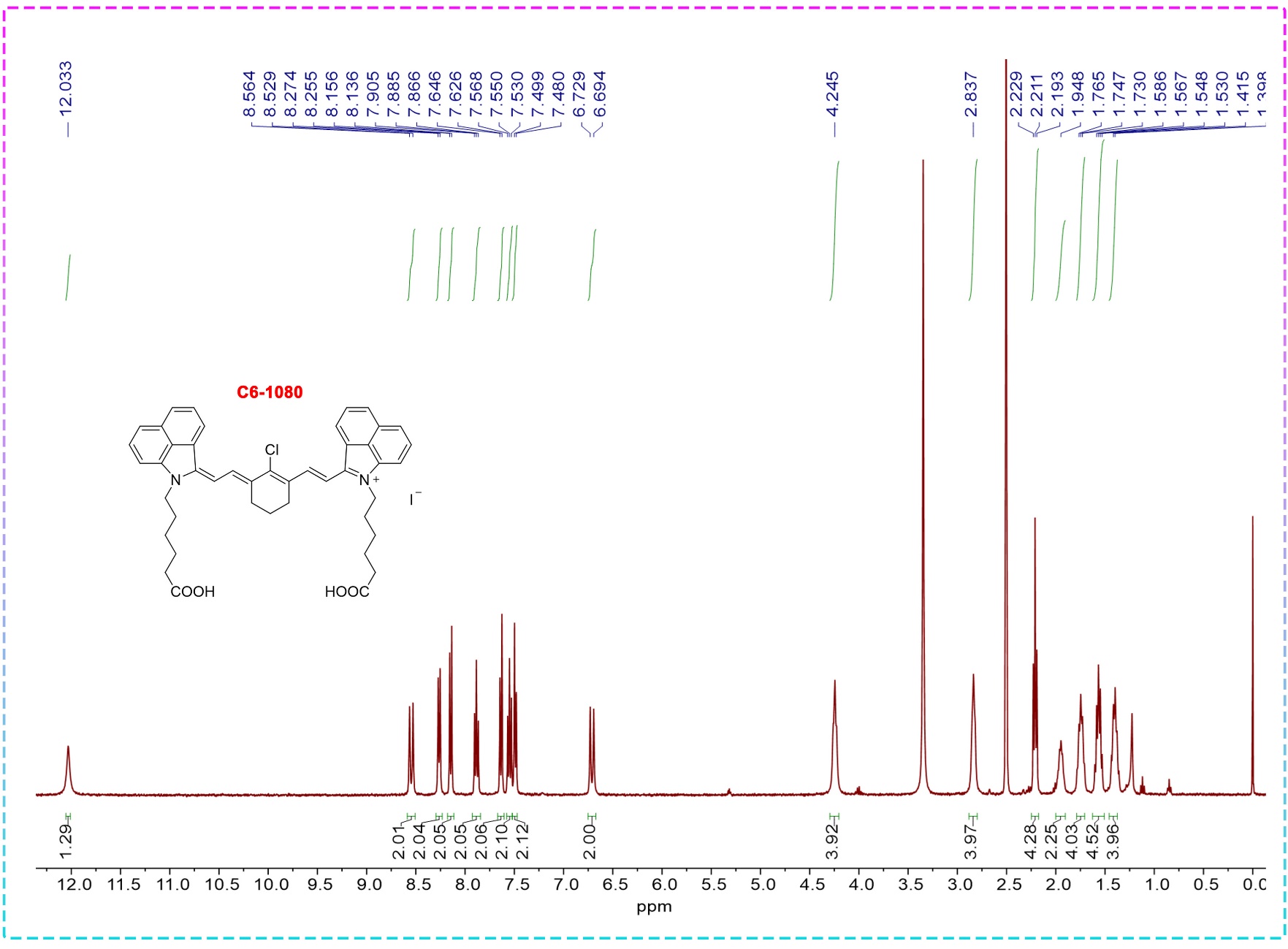
**

**^1^H-NMR spectrum of C6-1080 in DMSO-d6.**

**
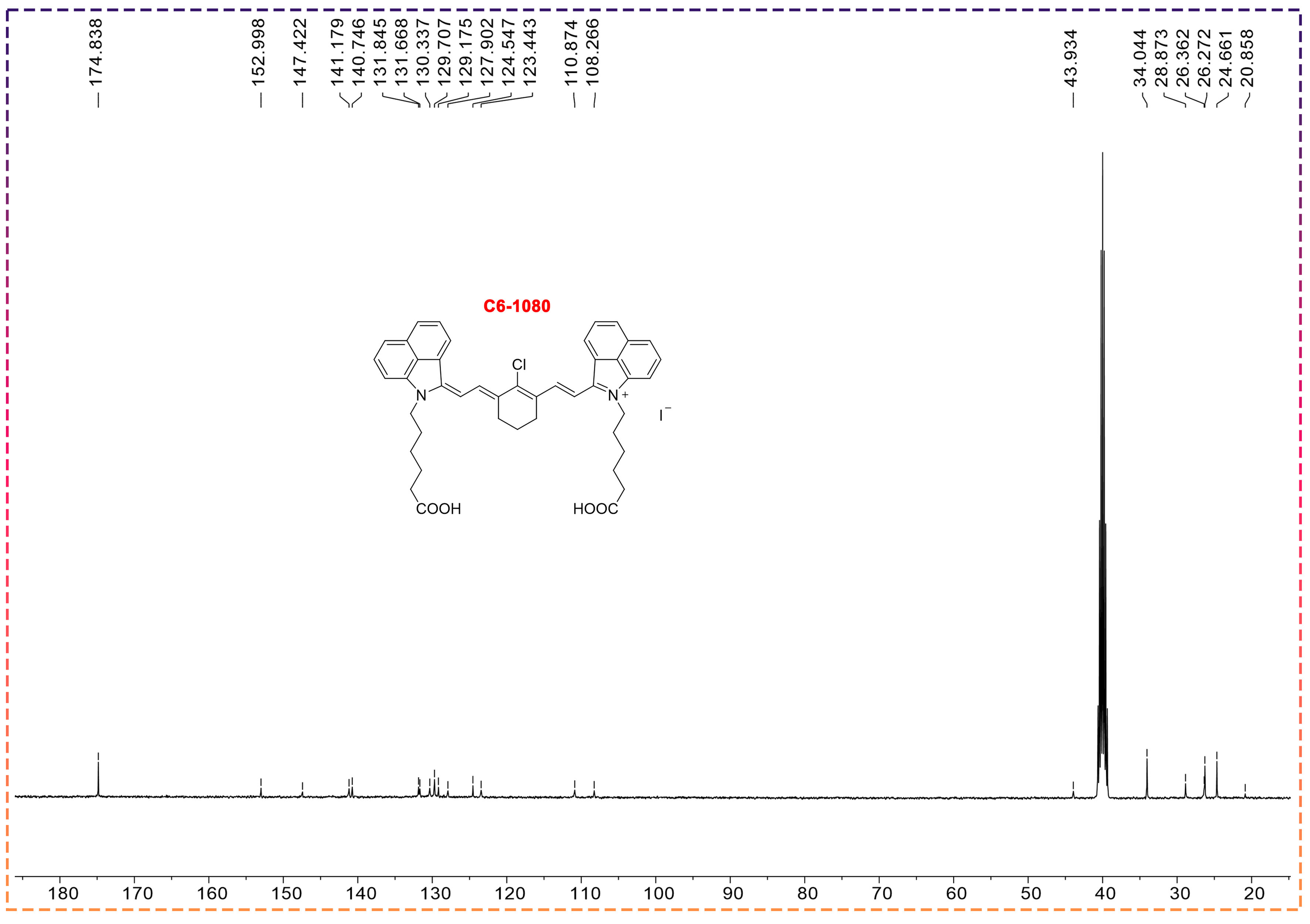
**

**^13^C NMR spectrum of C6-1080 in DMSO-d6.**

**
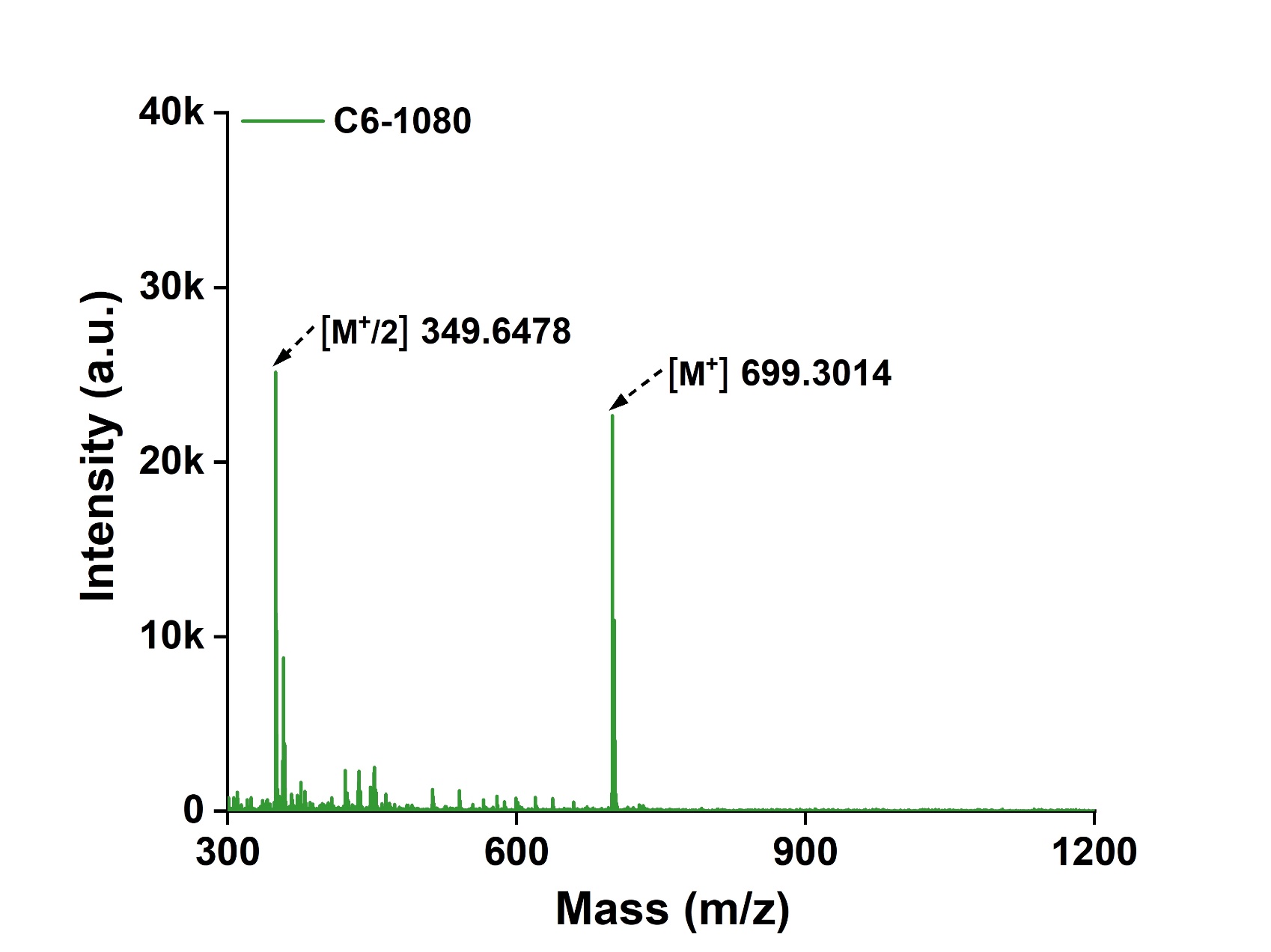
**

**LC-HRMS spectra of the C6-1080.**

**
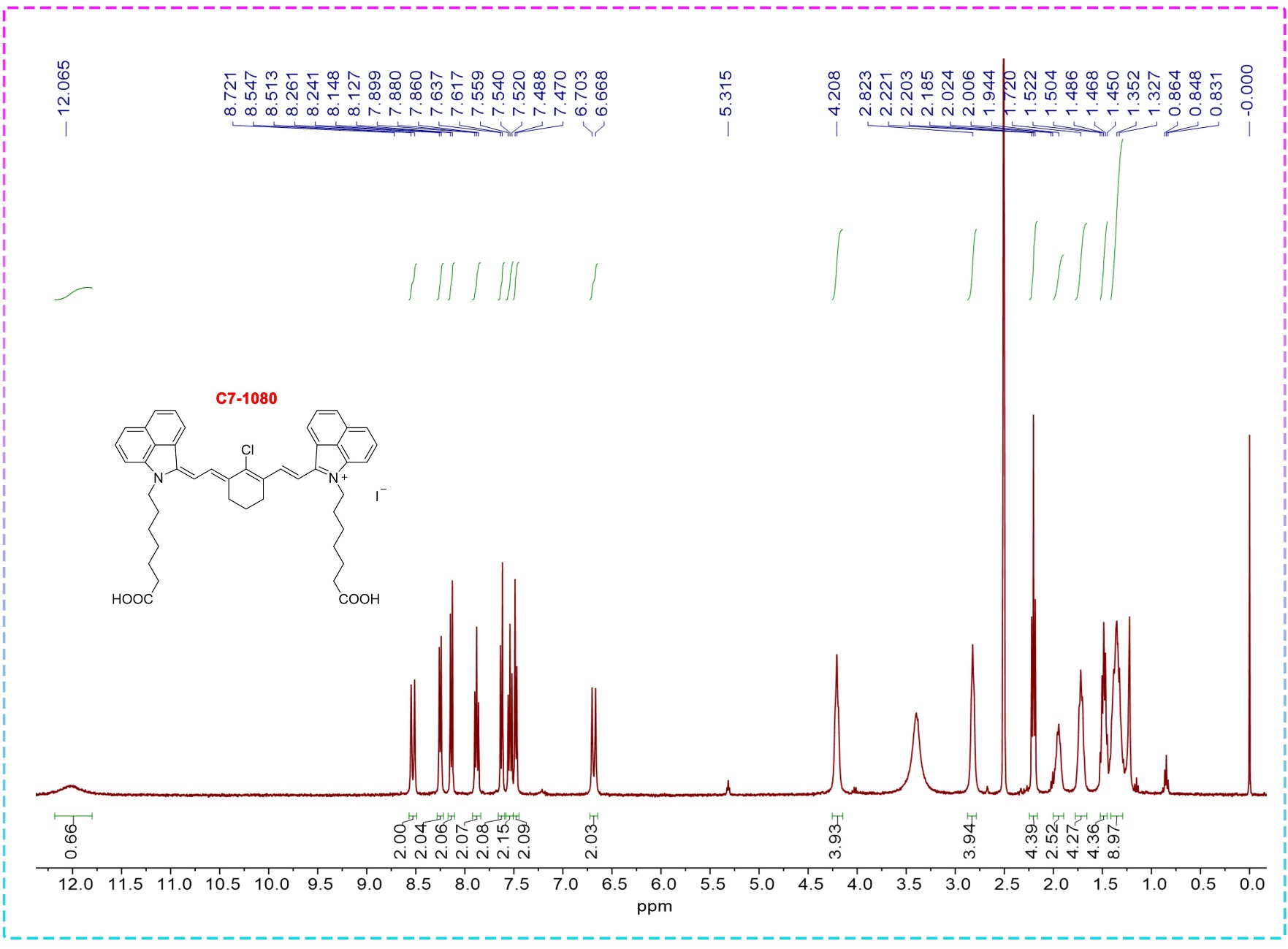
**

**^1^H-NMR spectrum of C7-1080 in DMSO-d6.**


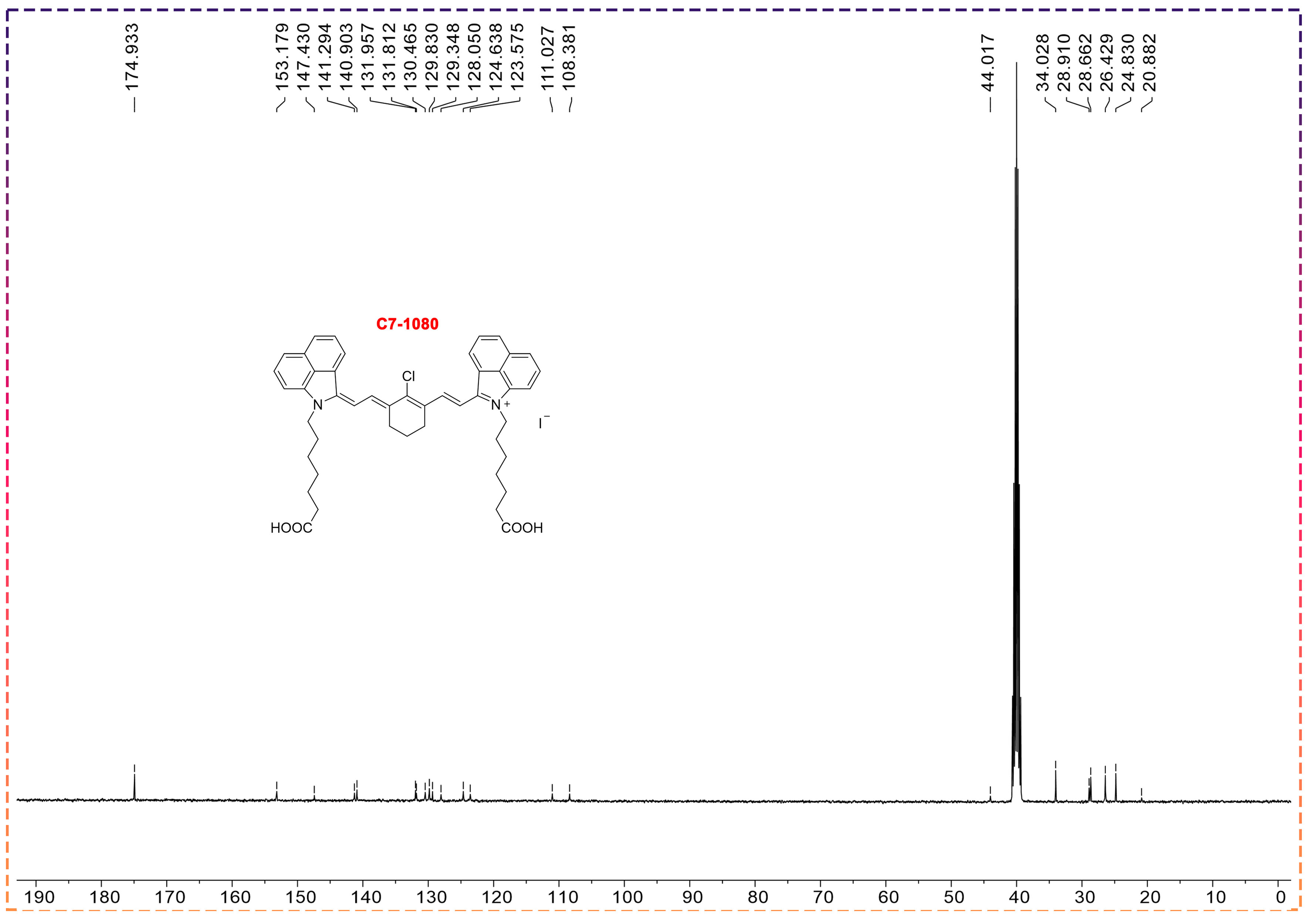


**^13^C NMR spectrum of C7-1080 in DMSO-d6.**

**
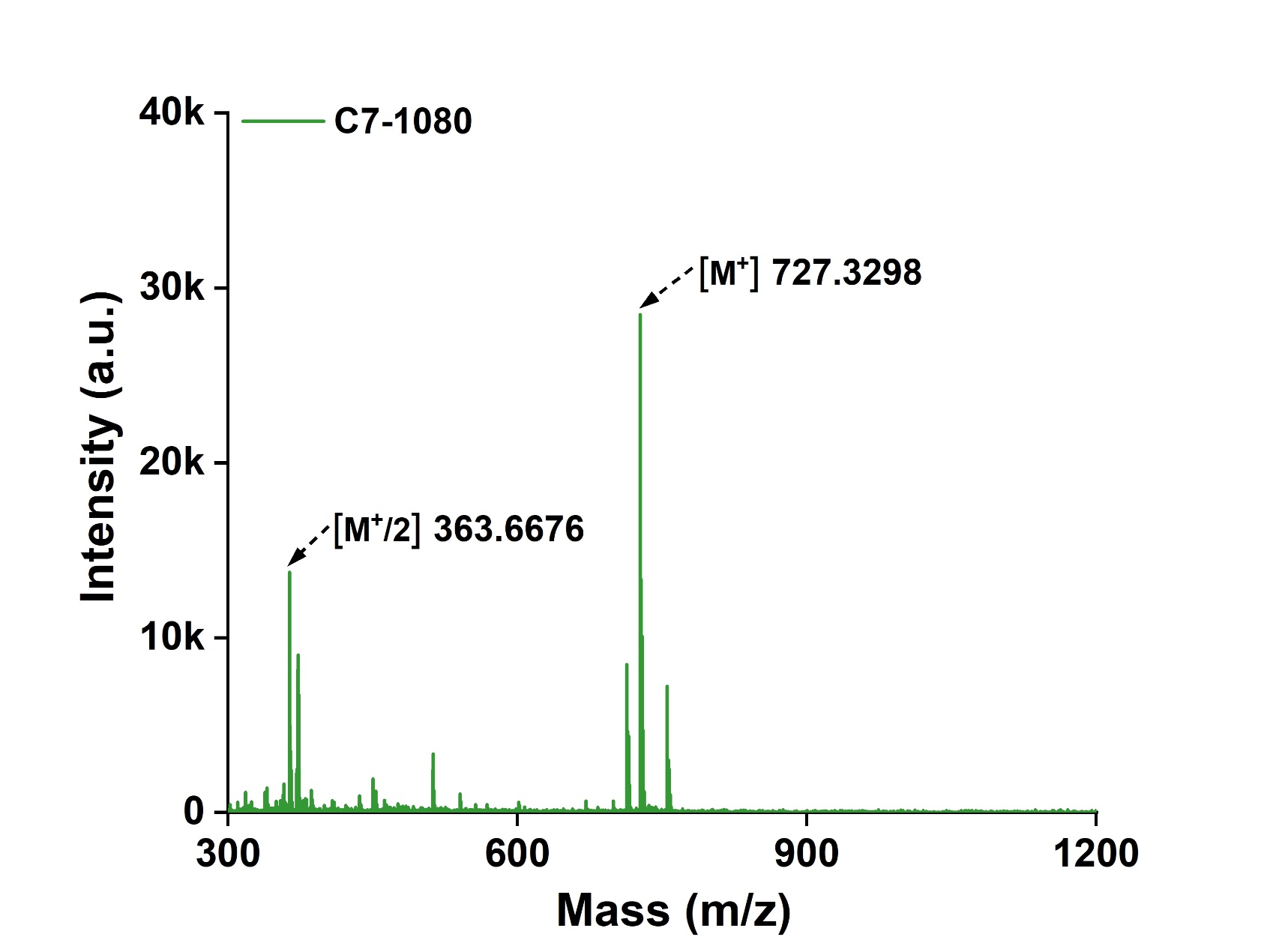
**

**LC-HRMS spectra of the C7-1080.**

**
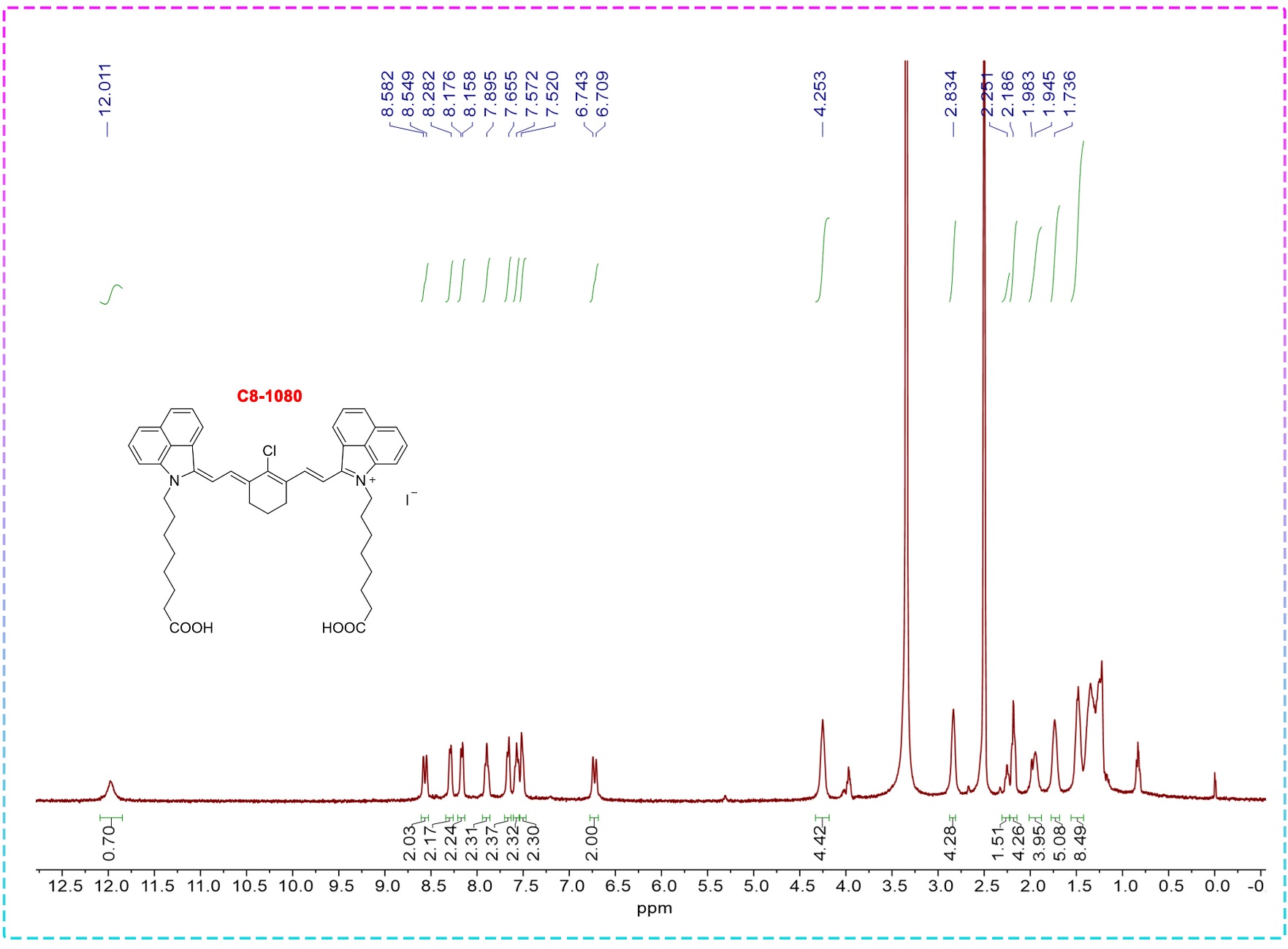
**

**^1^H-NMR spectrum of C8-1080 in DMSO-d6.**

**
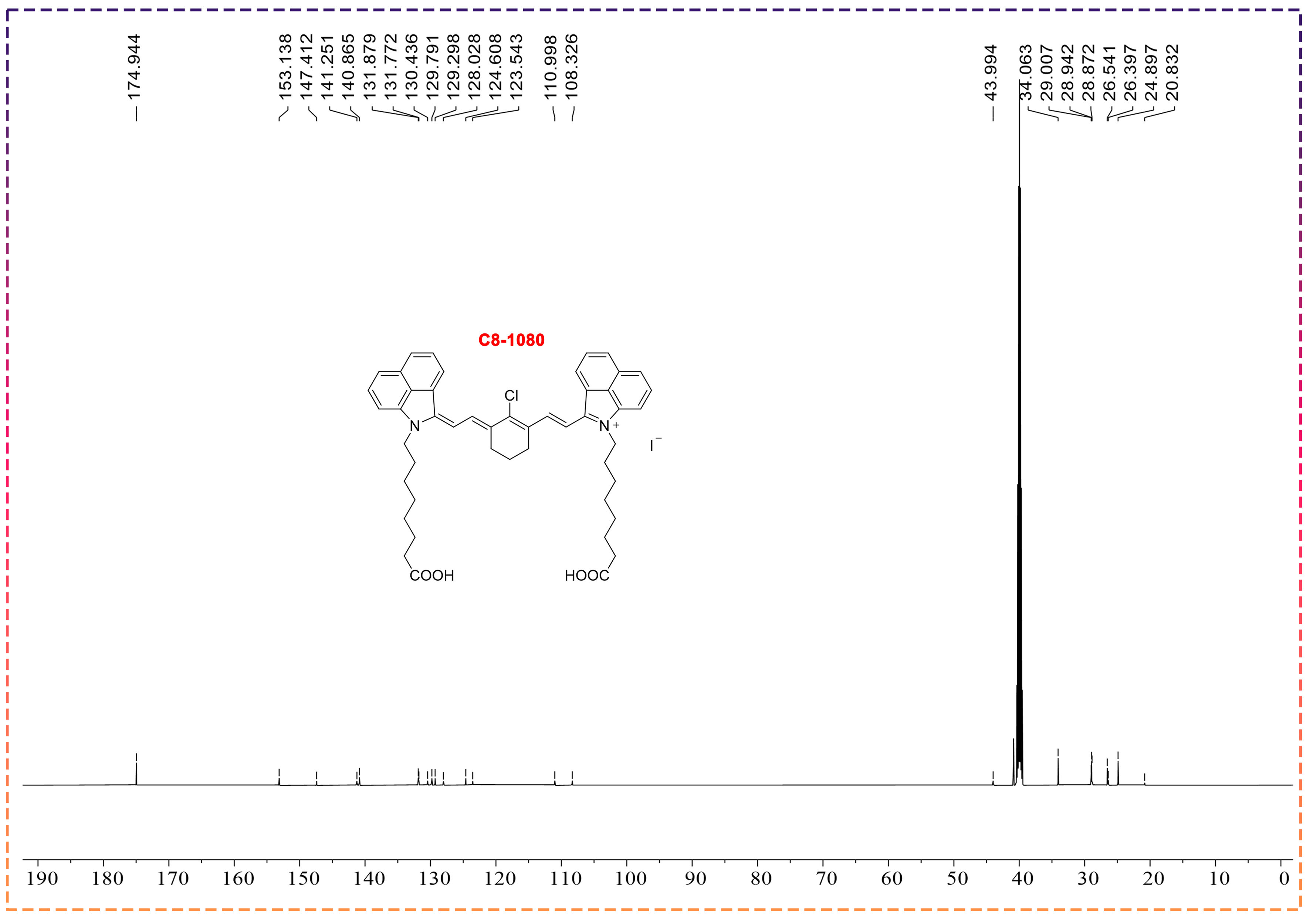
**

**^13^C NMR spectrum of C8-1080 in DMSO-d6.**

**
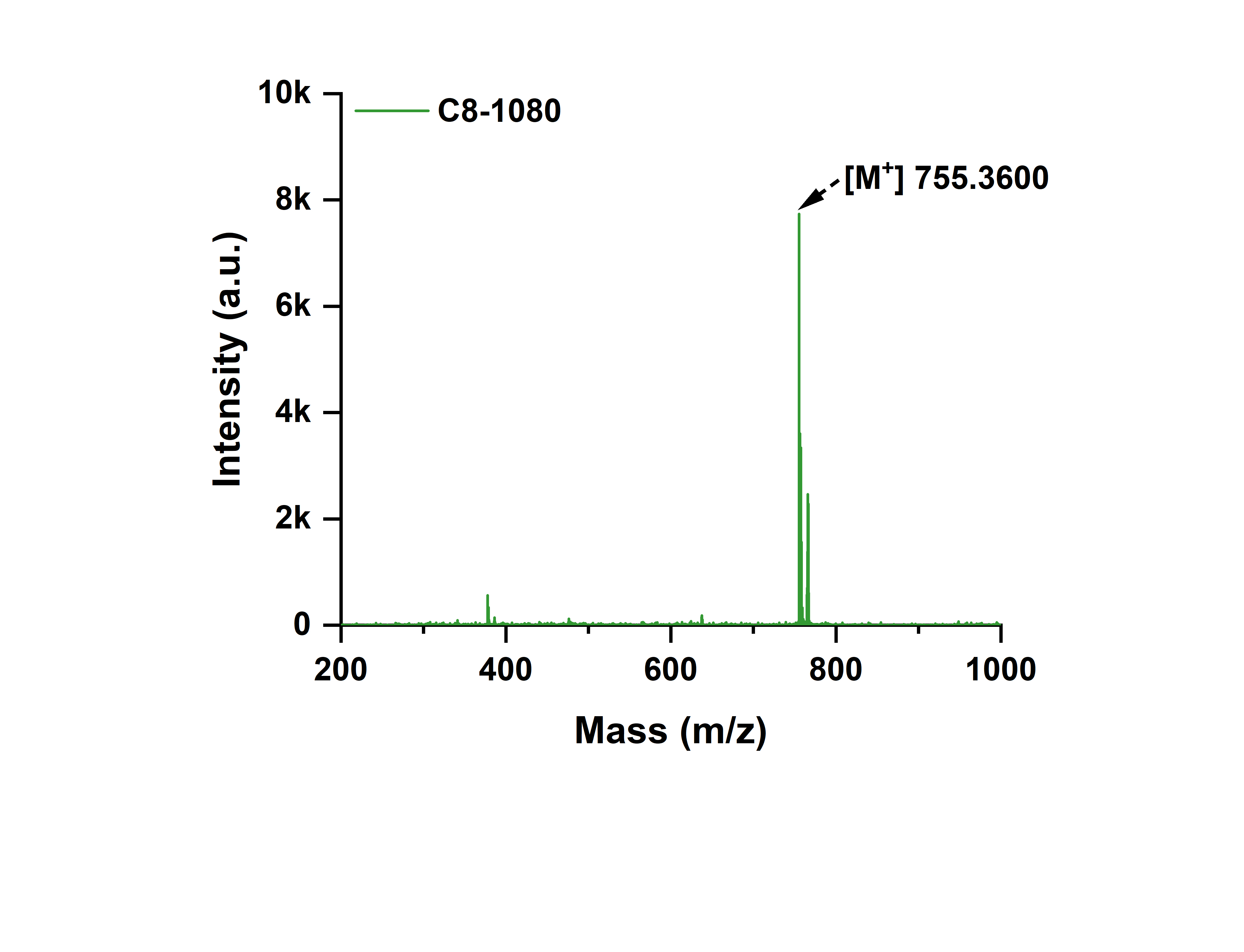
**

**LC-HRMS spectra of the C8-1080.**

**
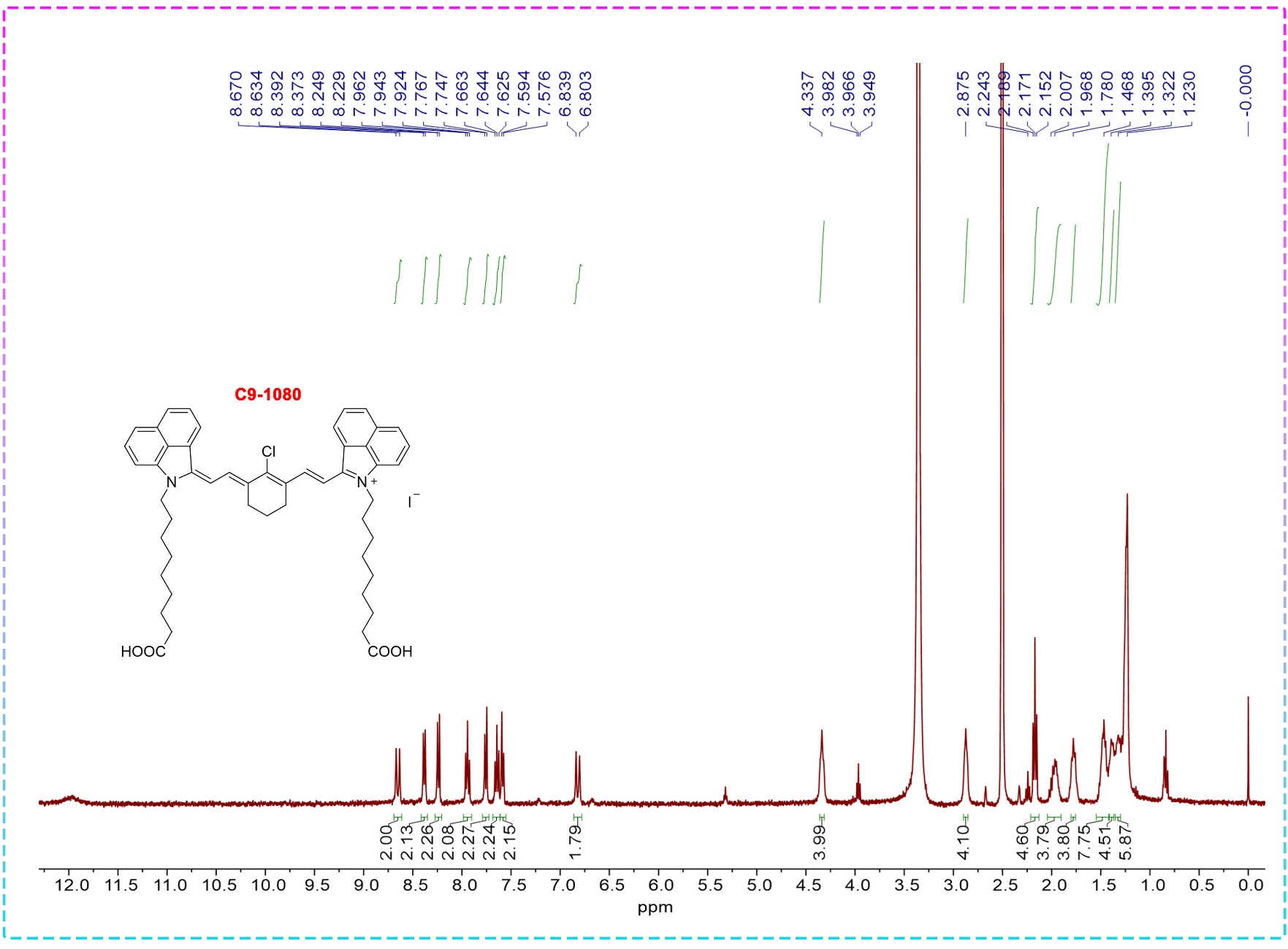
**

**^1^H-NMR spectrum of C9-1080 in DMSO-d6.**

**
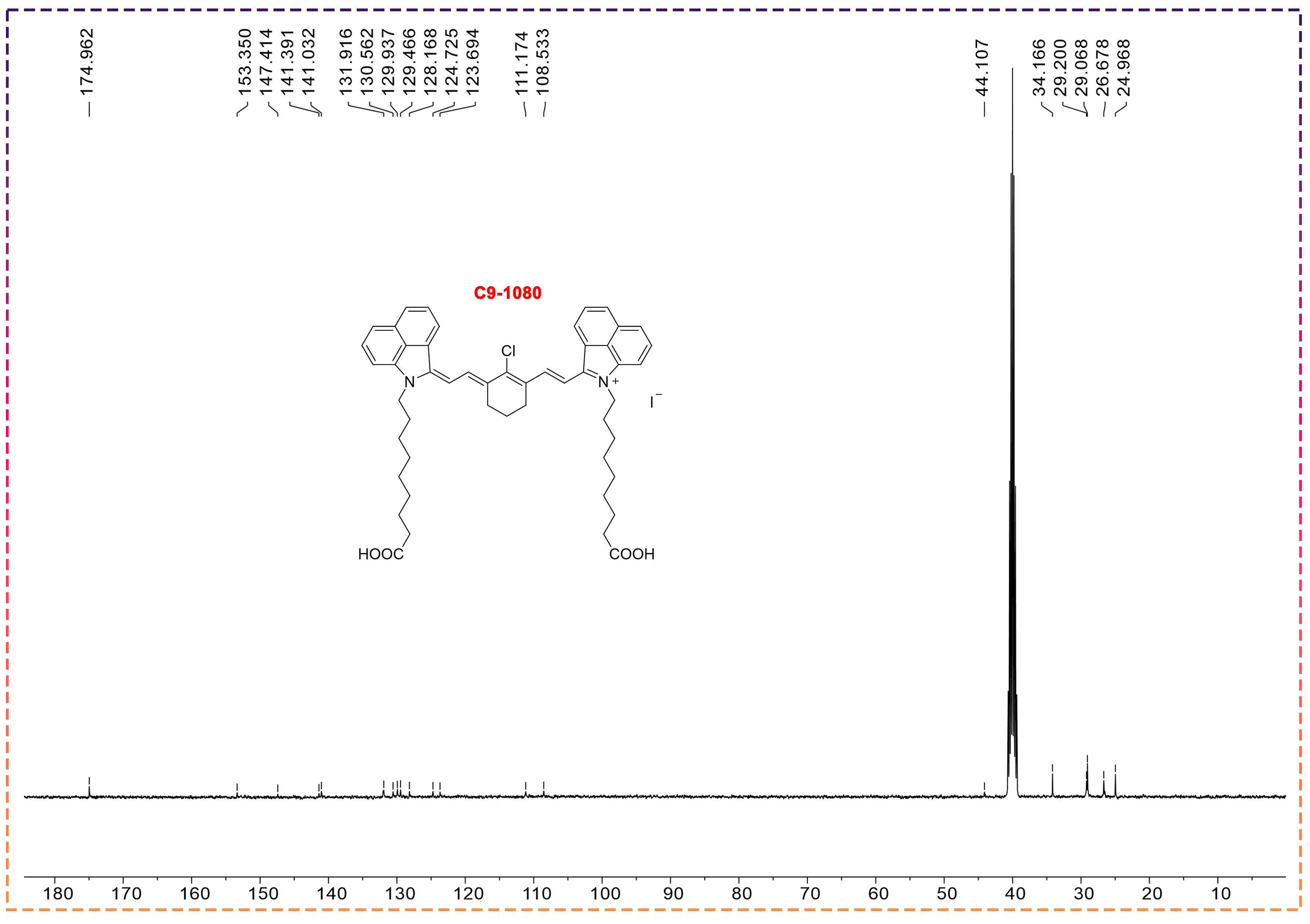
**

**^13^C NMR spectrum of C9-1080 in DMSO-d6.**


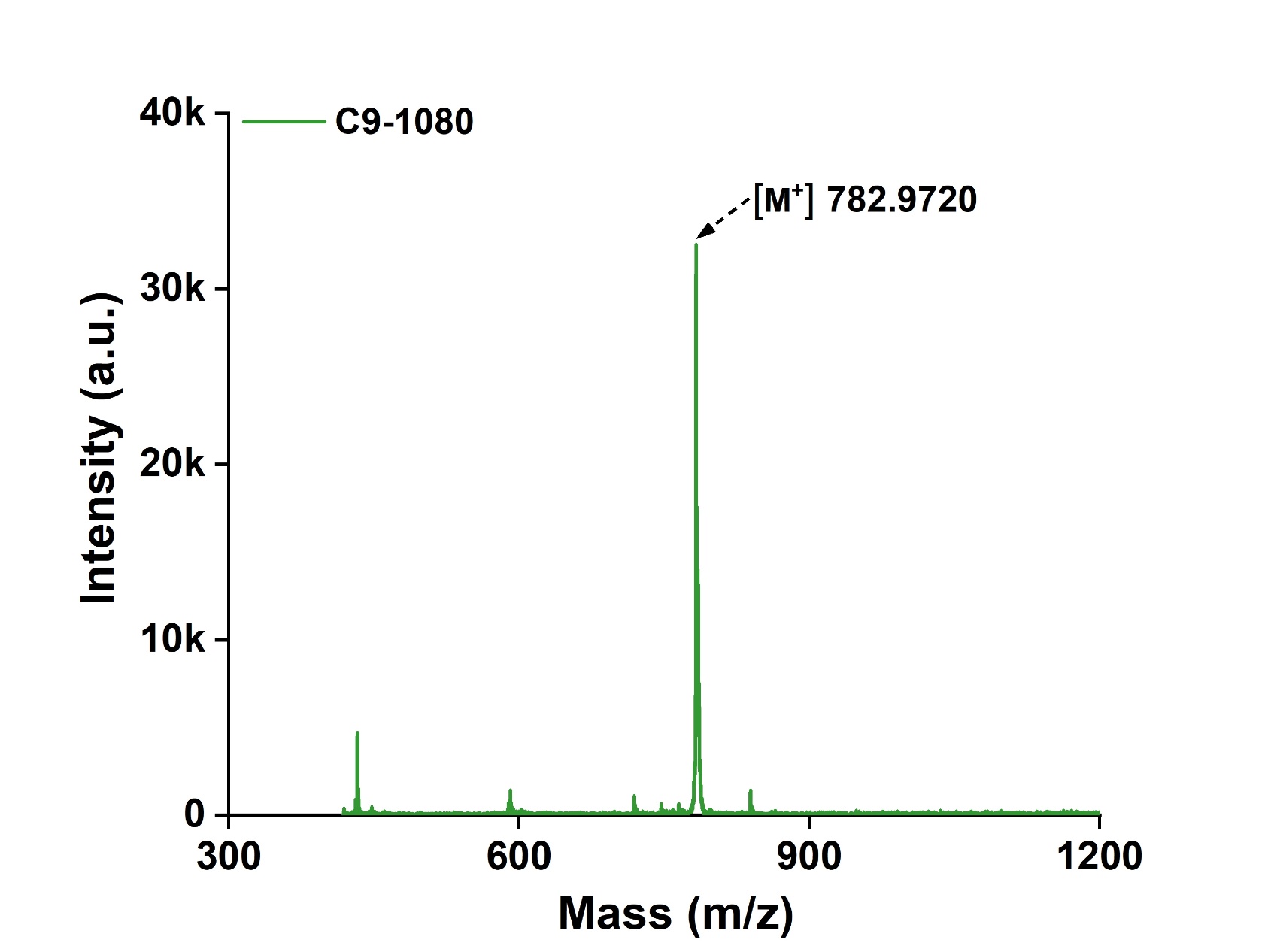


**LC-HRMS spectra of the C9-1080.**

**
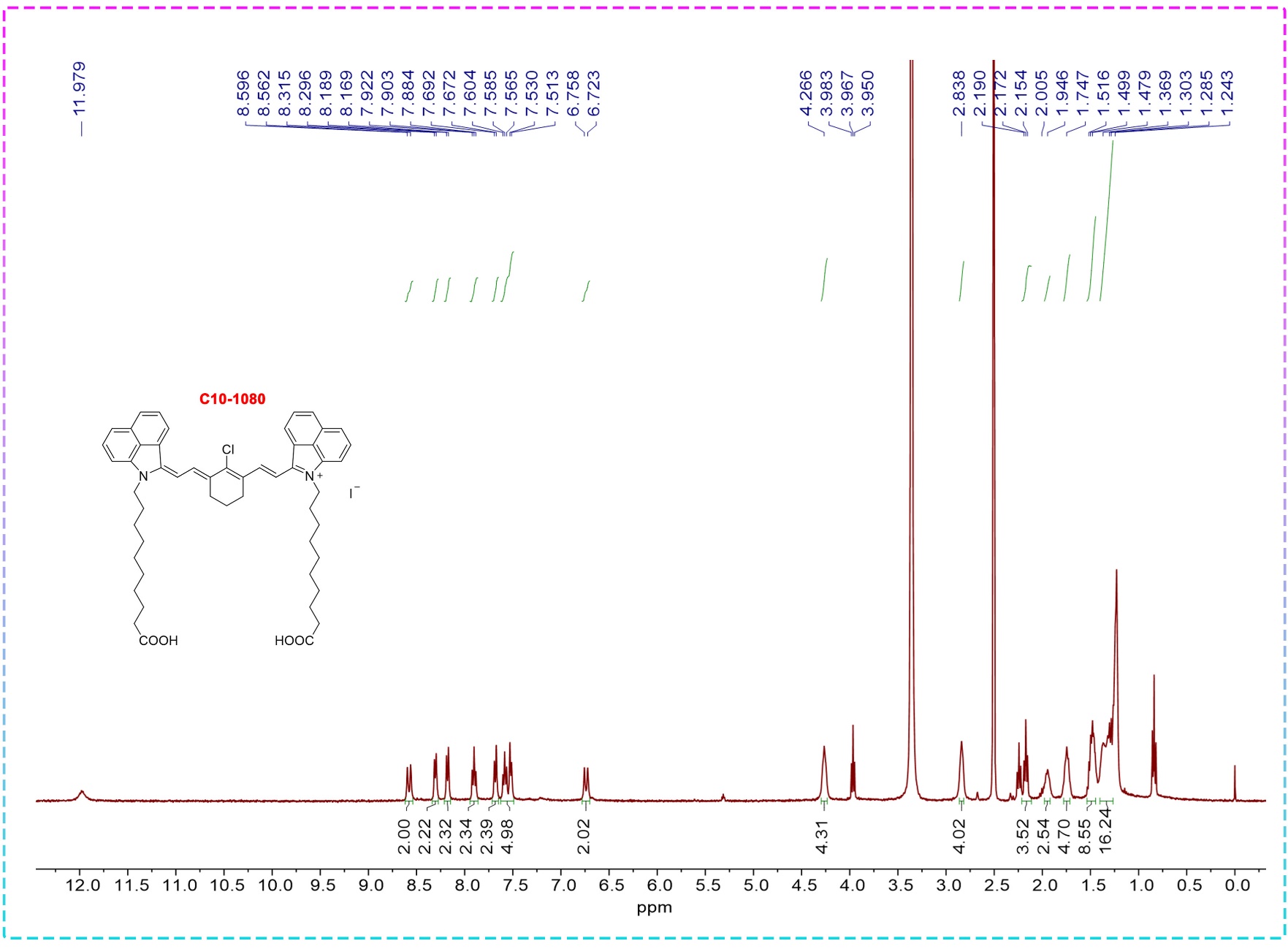
**

**^1^H-NMR spectrum of C10-1080 in DMSO-d6.**

**
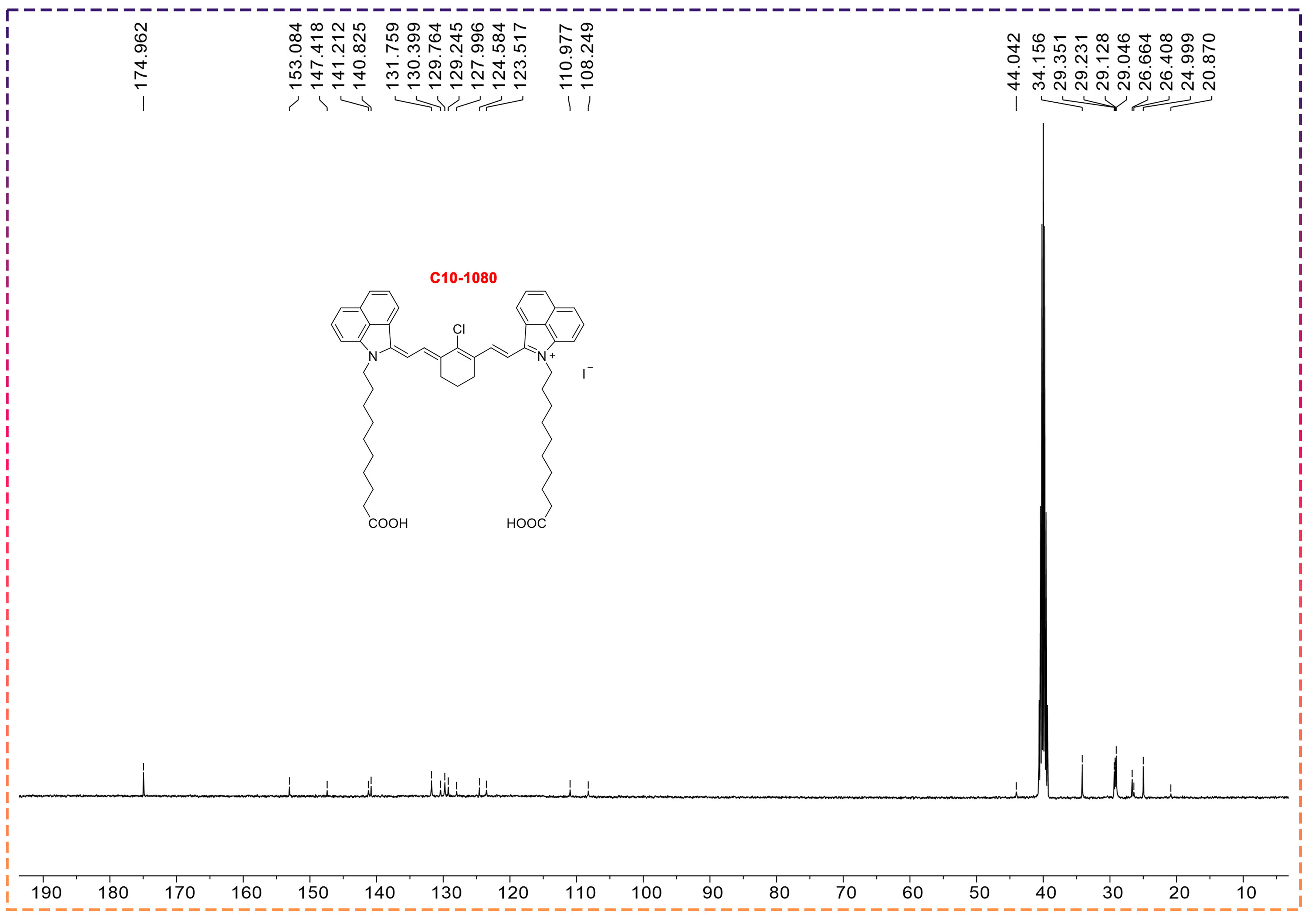
**

**^13^C NMR spectrum of C10-1080 in DMSO-d6.**

**
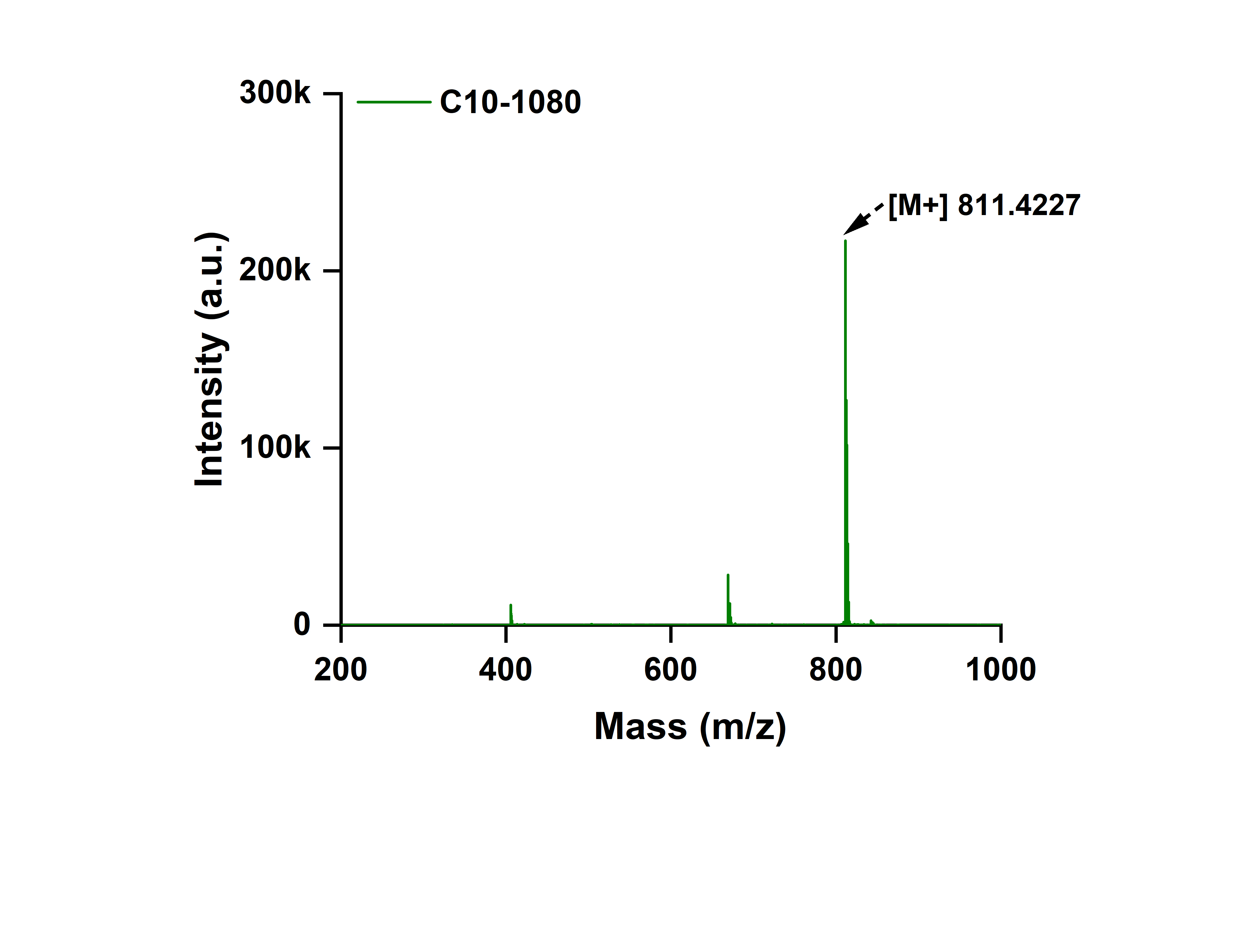
**

**LC-HRMS spectra of the C10-1080.**

**
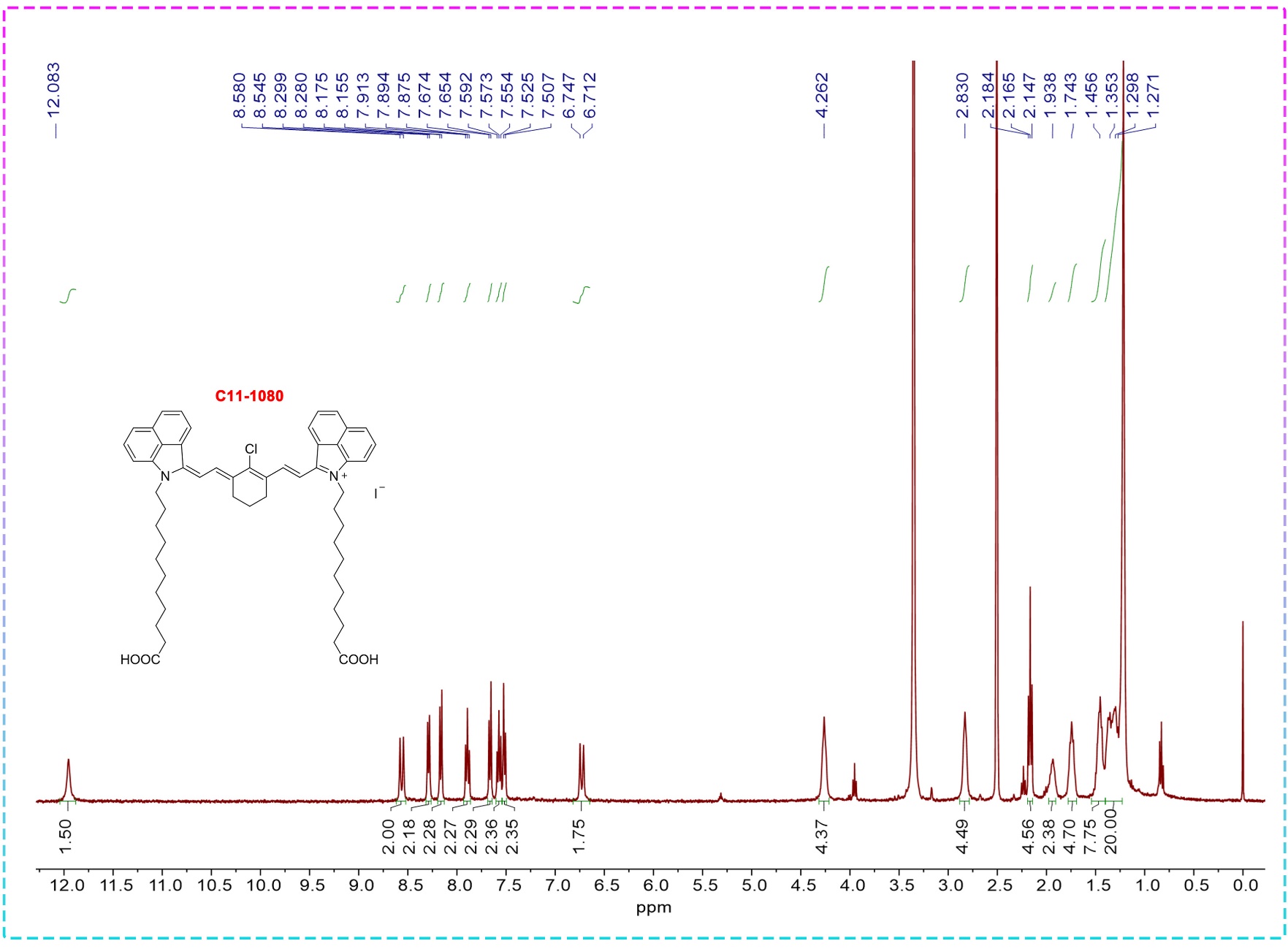
**

**^1^H-NMR spectrum of C11-1080 in DMSO-d6.**

**
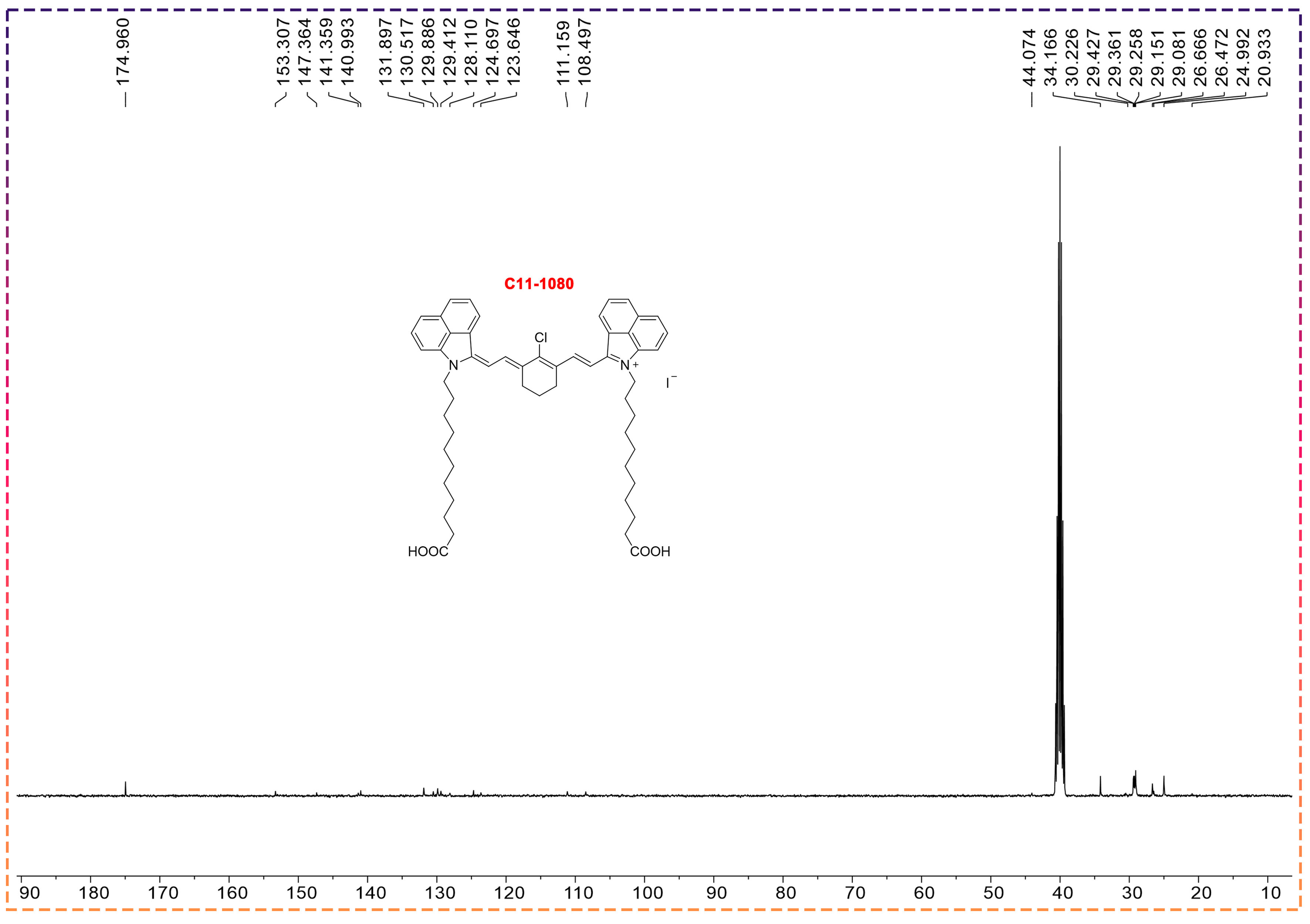
**

**^13^C NMR spectrum of C11-1080 in DMSO-d6.**

**
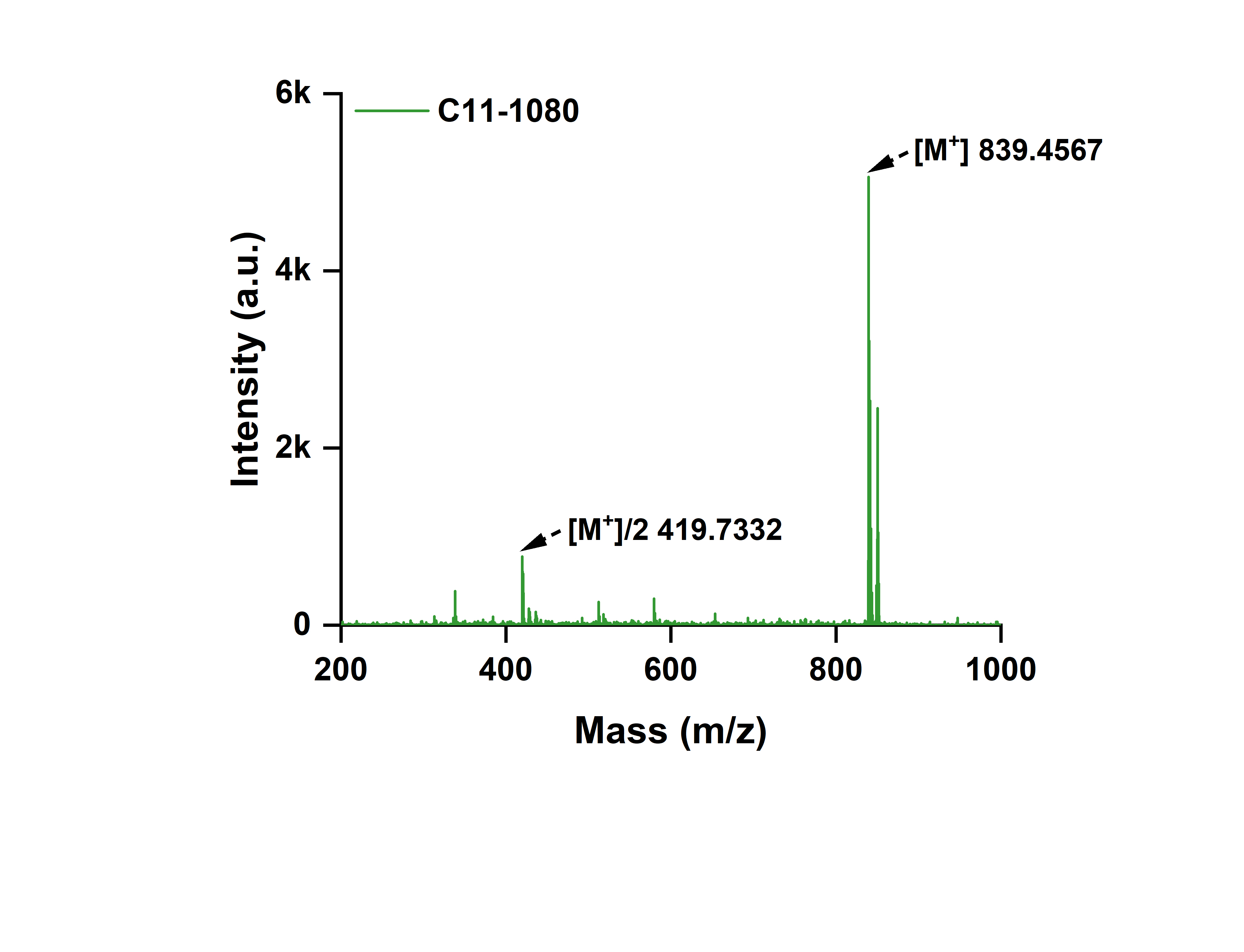
**

**LC-HRMS spectra of the C11-1080.**

**
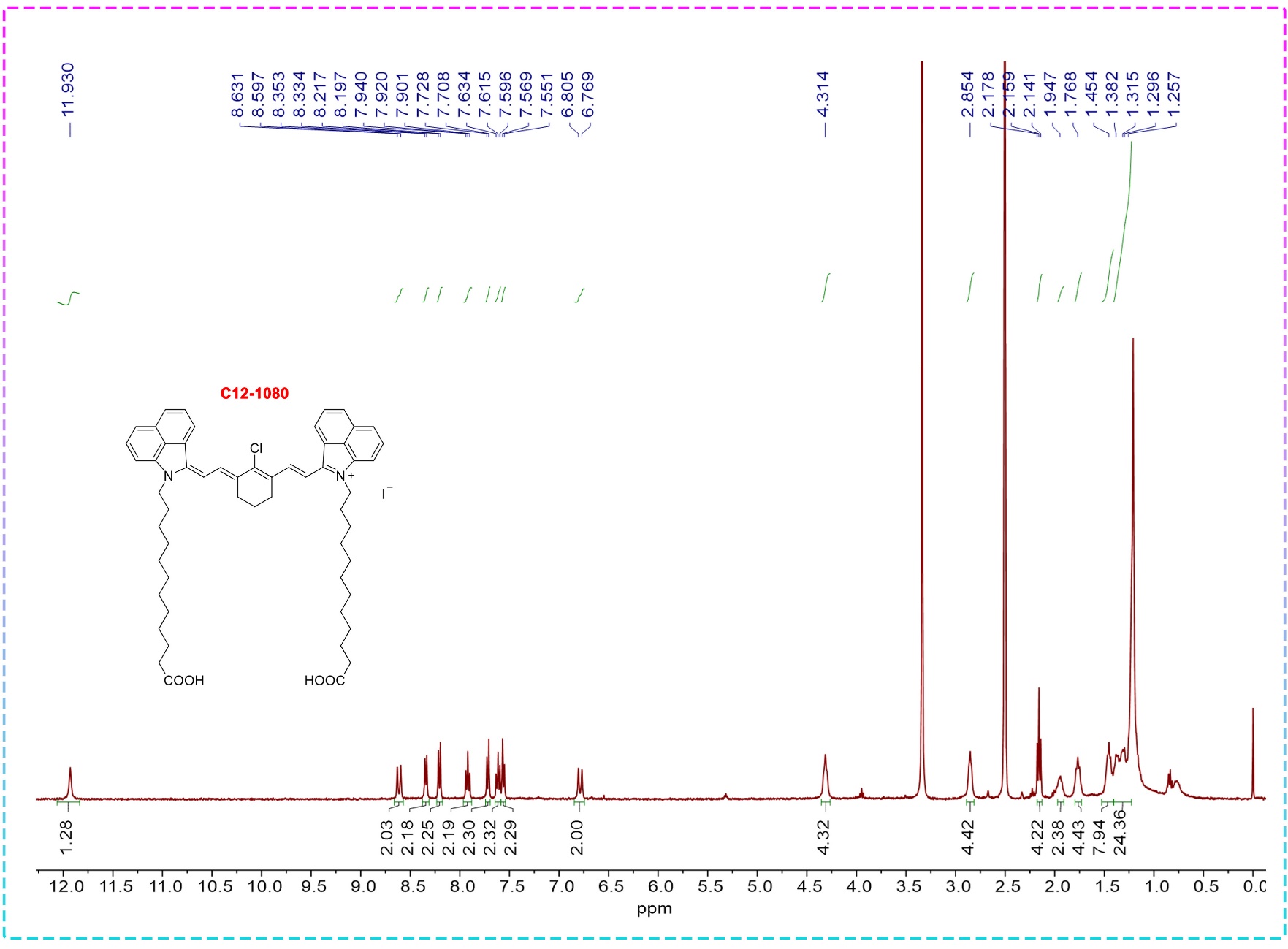
**

**^1^H-NMR spectrum of C12-1080 in DMSO-d6.**

**Note：**the effective ^13^C-NMR spectra for C12-1080 compounds were not provided due to solubility limitations.

**
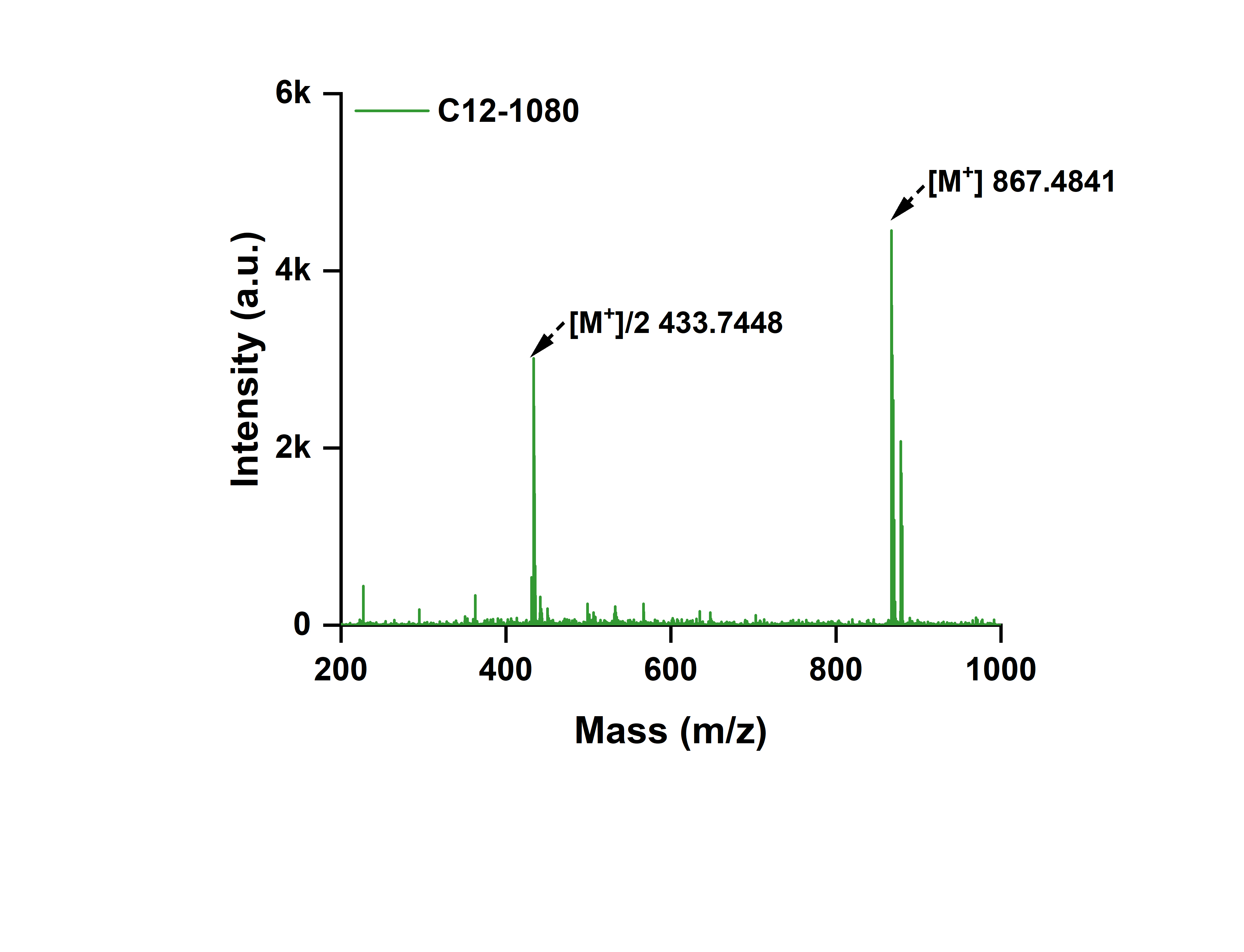
**

**LC-HRMS spectra of the C12-1080.**

**References**

[1] N. Zhu, J. Xu, Q. Su, T. Han, D. Zhou, Y. Zhang, S. Zhu, *Theranostics* **2024**, *14*, 1860.

[2] J.-K. Lee, M.-S. Park, Y.-S. Kim, K.-S. Moon, S.-P. Joo, T.-S. Kim, J.-H. Kim, S.-H. Kim, *Surg. Neurol.* **2007**, *67*, 620.

[3] A. B. Uzdensky, *Transl. Stroke Res.* **2018**, *9*, 437.

[4] Y. Kim, Y. B. Lee, S. K. Bae, S. S. Oh, J. Choi, *Sci. Rep.* **2021**, *11*, 5787.
